# Molecular interactions of FG nucleoporin repeats at high resolution

**DOI:** 10.1038/s41557-022-01035-7

**Published:** 2022-09-22

**Authors:** Alain Ibáñez de Opakua, James A. Geraets, Benedikt Frieg, Christian Dienemann, Adriana Savastano, Marija Rankovic, Maria-Sol Cima-Omori, Gunnar F. Schröder, Markus Zweckstetter

**Affiliations:** 1 German Center for Neurodegenerative Diseases, Göttingen, Germany; 2 Institute of Biological Information Processing (Structural Biochemistry), Forschungszentrum Jülich GmbH, Jülich, Germany; 3 Max Planck Institute for Multidisciplinary Sciences, Department of Molecular Biology, Göttingen, Germany; 4 Max Planck Institute for Multidisciplinary Sciences, Department of NMR-based Structural Biology, Göttingen, Germany; 5 Physics Department, Heinrich Heine University Düsseldorf, Düsseldorf, Germany

**Keywords:** Cryoelectron microscopy, Biophysical chemistry, Solution-state NMR

## Abstract

Proteins that contain repeat phenylalanine-glycine (FG) residues phase separate into oncogenic transcription factor condensates in malignant leukaemias, form the permeability barrier of the nuclear pore complex and mislocalize in neurodegenerative diseases. Insights into the molecular interactions of FG-repeat nucleoporins have, however, remained largely elusive. Using a combination of NMR spectroscopy and cryoelectron microscopy, we have identified uniformly spaced segments of transient β-structure and a stable preformed α-helix recognized by messenger RNA export factors in the FG-repeat domain of human nucleoporin 98 (Nup98). In addition, we have determined at high resolution the molecular organization of reversible FG–FG interactions in amyloid fibrils formed by a highly aggregation-prone segment in Nup98. We have further demonstrated that amyloid-like aggregates of the FG-repeat domain of Nup98 have low stability and are reversible. Our results provide critical insights into the molecular interactions underlying the self-association and phase separation of FG-repeat nucleoporins in physiological and pathological cell activities.

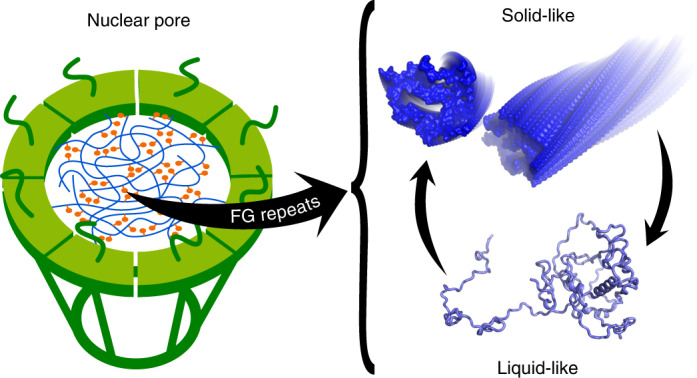

## Abstract (web summary)

Proteins rich in phenylalanine-glycine (FG) repeats can phase separate through FG–FG interactions. The molecular interactions of an important FG-repeat protein, nucleoporin 98, have now been studied in liquid-like transient and amyloid-like cohesive states. These interactions underlie the behaviour of FG-repeat proteins and their function in physiological and pathological cell activities.

## Main

Phenylalanine-glycine (FG) repeats are present in many intrinsically disordered proteins and have been linked to multiple cellular processes^1–4^. Sequence analysis has identified more than 600 proteins containing FG repeats^5^. Nucleoporins containing FG repeats form the permeability barrier of the nuclear pore complex^4,6–8^. In addition, they are involved in cancer-associated biomolecular condensates, the so-called oncogenic transcription factor condensates^9–12^. FG-repeat proteins are also present in several other membraneless organelles^3^. Increasing evidence further links cellular mislocalization of FG-repeat-containing nucleoporins to pathological protein misfolding and aggregation in neurodegenerative diseases, including Alzheimer’s disease, amyotrophic lateral sclerosis and frontotemporal dementia^13,14^. Mutagenesis in combination with functional assays has provided ample support for the critical role of FG repeats in these cellular processes. However, the nature of the underlying molecular interactions between FG repeats is largely unknown.

An important FG-repeat protein associated with multiple physiological and pathological processes is the human nucleoporin 98 (Nup98)^2^. In certain types of leukaemia, the FG-repeat domain of Nup98 is fused to a chromatin-binding domain as a result of recurrent chromosomal translocations^15^. The oncogenic properties of the Nup98 fusion proteins are related to their ability to concentrate into condensates^9–12^. Site-directed mutagenesis demonstrates that the ability to self-associate and form oncogenic transcription factor condensates critically depends on the FG repeats of Nup98 (refs. ^9,11,12^). Consistent with the formation of Nup98 condensates in cells, the FG repeats of several nucleoporins phase separate in vitro into liquid-like droplets and solid-like condensates above critical concentrations as low as 20 nM (refs. ^5,16^). The biophysical properties of particles and gels of FG-repeat Nups have been characterized previously in great detail^5,17–20^. The FG-repeat domain of Nup98 also facilitates the aggregation of the protein tau associated with Alzheimer’s disease in vitro and accumulates in the cell bodies of neurons that contain tau aggregates^14^.

Through a combination of NMR spectroscopy and cryoelectron microscopy (cryo-EM), here we provide insights into the dynamic structure of the FG-repeat domain of Nup98 at the single residue level, reveal a stable preformed structure and define the molecular organization of cohesive FG–FG interactions in reversible FG clusters at high resolution.

## Results

### Molecular organization of the FG-repeat domain of human Nup98

**Fig. 1:**
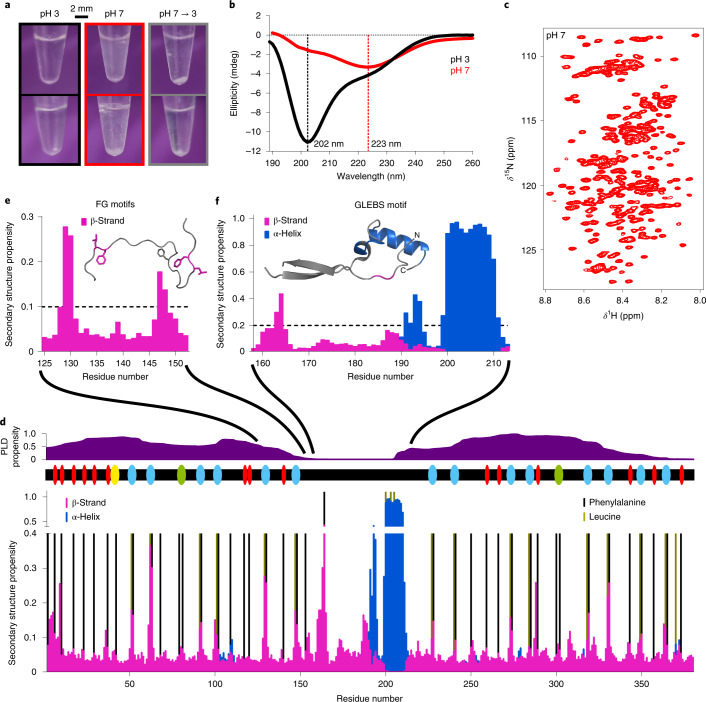
Dynamic structure of Nup98^FG^. **a**, Macroscopic changes in samples of the FG-repeat domain of Nup98^FG^ at pH 3, after adjusting to pH 7 and then back to pH 3 before incubation (top row) and after incubation at 65 °C for 30 min (bottom row). **b**, CD spectra of Nup98^FG^ in the soluble phase (pH 3) and in the condensed/aggregated phase (pH 7). **c**, Two-dimensional ^1^H–^15^N heteronuclear single quantum coherence spectrum of Nup98^FG^ at pH 7 (started ~5 min after adjusting the pH from 3 to 7). **d**, The conformational properties of soluble Nup98^FG^ at pH 6.8. The likelihood of residue-specific backbone torsion was determined from the experimental NMR chemical shifts using TALOS-N. The propensity for prion-like domain (PLD) structure and the location of FG motifs are shown above (red, FG; yellow, SAFG; cyan, GLFG; green, FXFG). **e**, The β-strand motifs in the N-terminal prion-like domain (taken from **d**). The conformation derived from TALOS-N is shown as the inset; the colouring is based on the threshold propensity shown in the graph. Phenylalanine and leucine side chains are displayed. **f**, Preformed secondary structure of the GLEBS-binding motif of monomeric unbound Nup98^FG^ (taken from **d**). The inset shows the crystal structure (Protein Data Bank identification (PDB ID): 3MMY) of the GLEBS-binding motif in the complex with the mRNA export factor Rae1. Regions of the crystal structure that are preformed (>0.2) prior to binding to Rae1 are coloured (the α-helical structure is shown in blue and the β-structure in magenta). Source data

The amino-terminal 384 residues of human Nup98 (named Nup98^FG^) have a high density of FG repeats comprising in total 41 phenylalanine residues. We recombinantly expressed and purified Nup98^FG^ (see Methods and Supplementary Materials). Nup98^FG^ is soluble and predominantly disordered at pH 3 (Fig. 1a,b and Supplementary Fig. 1). This is in agreement with previous reports that at acidic pH the net charge of proteins changes and the hydrophobic interactions of aromatic rings are attenuated, promoting the solubility of proteins that are highly insoluble at native pH (refs. ^21,22^).

To gain insight into the soluble structure of Nup98^FG^, we performed extensive NMR measurements on both Nup98^FG^ and a large number of shorter Nup98^FG^ segments (Supplementary Figs. 2 and 3). In addition, NMR spectra were recorded at both native pH and pH 3 (Fig. 1c and Supplementary Fig. 2). Through this integrative approach, we overcame the challenges of a highly repetitive sequence and strong aggregation tendency^5^ and determined the sequence-specific resonance assignment of Nup98^FG^ at native pH (Supplementary Table 1). The sequence-specific assignment showed that uniformly spaced, short segments of extended β-structure (Fig. 1d,e), which coincide with phenylalanine residues, are abundant in the FG-repeat domain of Nup98.

### Preformed structure for messenger RNA export factor binding

The nuclear membrane spatially separates transcription and translation. The mRNA synthesized in the nucleus has to cross the nuclear membrane by passing through the permeability barrier of the nuclear pore complex. This passage is achieved with the help of mRNA export factors. The mRNA export factor Rae1 binds to the conserved Gle2-binding sequence (GLEBS; residues 157–213)^23^, which is located between the two prion-like FG-rich regions of Nup98 (Fig. 1d,f). When complexed with Rae1, the GLEBS domain of Nup98 folds into two short β-strands, a short α-helix and a longer α-helix comprising residues 200–210 (the structure is displayed in the inset of Fig. 1f)^23^. Residue-specific analysis of the NMR chemical shifts (Fig. 1d) revealed that residues 200–210 of Nup98^FG^, which contain the core GLEBS motif^24^ and form the long α-helix in the complex with Rae1 (ref. ^23^), are folded into a stable α-helix prior to binding to Rae1 (Fig. 1f). The prefolded α-helical structure of the GLEBS motif may decrease the entropic costs of binding to Rae1, thereby promoting the interaction of the GLEBS motif of Nup98 with Rae1.

### Amyloid-like interactions of Nup98 FG repeats

To gain insight into the structure of cohesive FG–FG interactions, we studied the molecular properties of aggregated Nup98^FG^. When we changed the pH from 3 to 7, solutions of Nup98^FG^ rapidly turned turbid (Fig. 1a) and the circular dichroism (CD) spectrum changed markedly (Fig. 1b). For a ‘regular’ β-structure, a minimum at 218 nm and a maximum at 200 nm are expected. The CD spectrum of Nup98^FG^ at pH 7, however, does not exhibit a maximum at 200 nm and the minimum is located at 223 nm. A minimum at ~223 nm was previously observed for fibrillar amyloid-β(1–40)^25^, while the lack of the maximum at 200 nm is probably due to a substantial amount of remaining disordered structure in Nup98^FG^ at pH 7.

**Fig. 2:**
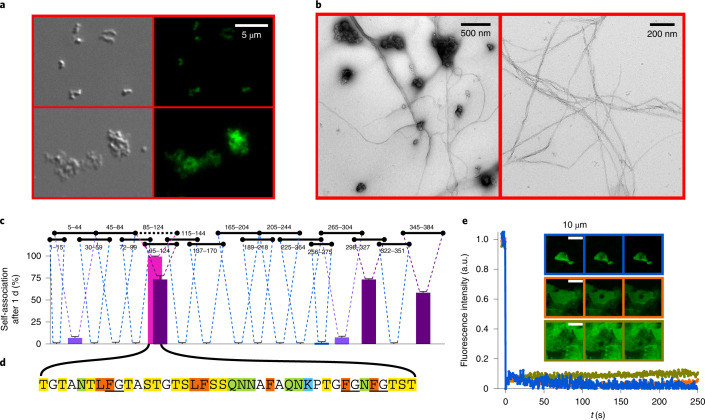
Nup98^FG^ forms an amyloid-like structure. **a**, Differential interference contrast (left) and fluorescence (right) microscopy images of two examples of aggregates/condensates formed by Nup98^FG^. The fluorescence originates from staining with ThT. **b**, Negative-stain EM images of Nup98^FG^ aggregates. **c**, Self-association propensities of 18 Nup98^FG^ fragments. The residue numbers of each fragment in the Nup98^FG^ sequence are indicated above the plot. The percentage propensity to self-association (condensation/aggregation) corresponds to the difference between the NMR signal expected for that peptide concentration and that observed after 1 day of incubation at 5 °C. High, intermediate and low self-association propensities are coloured magenta, purple and violet, respectively. For the fragments showing no propensity data, the NMR signal remained unchanged and the connecting dashed lines are shown in blue. The error bars represent the s.d. based on the NMR signal-to-noise ratio. **d**, Residues 85–124 of Nup98 (Nup98^FG85^), which are the most prone to self-association, as indicated in **c** (black horizontal dotted line). Phenylalanine and leucine are highlighted in orange, asparagine and glutamine in green, lysine in blue, and serine and threonine in yellow. The canonical FG motifs are underlined (notably, the glycine in FG motifs is sometimes replaced by other small amino acids, for example, serine^5^). **e**, Nup98^FG85^ forms solid-like particles that can be stained with ThT. The time plot shows that the bleached ThT fluorescence of three different particles did not recover within 250 s (the plot colour corresponds to the micrograph with the same colour outline). Source data

Further support for the pH-induced changes in the molecular properties of Nup98^FG^ was obtained by microscopy. At pH 7, clusters of particles were observed by differential interference contrast microscopy (Fig. 2a, left). Notably, the particles were fluorescent when we exposed the sample to the amyloid-specific dye thioflavin T (ThT) (Fig. 2a and Supplementary Fig. 4). In addition to these ThT-positive particles/particle clusters, amyloid fibrils were detected by electron microscopy (EM; Fig. 2b). Taken together, these experiments showed that the FG-repeat domain of Nup98, similar to the FG-repeat domains of other nucleoporins^26,27^, readily forms ThT-positive particles and amyloid-like structures at native pH.

To investigate whether specific regions in Nup98^FG^ favour the formation of amyloid-like aggregates, we studied the aggregation propensity of 18 shorter segments, which together cover the FG-repeat domain of Nup98 (Fig. 2c). We quantified the aggregation propensity of each segment by the time-dependent changes in NMR signal intensity. The analysis showed that the aggregation propensity varies markedly along the Nup98^FG^ sequence (Fig. 2c). The most aggregation-prone segment comprises residues 85–124 (named Nup98^FG85^; Fig. 2c). Nup98^FG85^ contains five phenylalanine residues, three in FG motifs (Fig. 2d). Two of the phenylalanines are preceded by leucine. Similar to the 384-residue Nup98^FG^ domain (Fig. 2a), Nup98^FG85^ forms ThT-positive particles/clusters (Fig. 2e and Supplementary Fig. 4). Fluorescence recovery after photobleaching (FRAP) experiments showed that bleached fluorescence did not recover with time (Fig. 2e), consistent with the solid-like nature of the Nup98^FG85^ particles/clusters. We have thus identified a highly aggregation-prone FG-rich segment in Nup98.

We also quantified the time-dependent decrease in the NMR signal of a slightly shorter peptide (residues 95–124 of Nup98; Fig. 2c) and compared this with the decrease in signal of two mutant peptides in which either two phenylalanine residues (F117 and F120) or two glutamine residues (Q105 and Q111) were replaced by serine (Supplementary Fig. 5). The glutamine-to-serine mutations reduced slightly the aggregation kinetics and resulted in a higher residual NMR-observable peptide concentration at the end of the incubation period. The impact of the phenylalanine-to-serine mutations was more pronounced, leading to a nearly twofold increase in the time for 50% of the peptide to become unobservable. This analysis suggests that both phenylalanine and glutamine are important contributors to the aggregation of Nup98^FG^.

### Cryo-EM structures of Nup98(85–124) fibrils

**Fig. 3:**
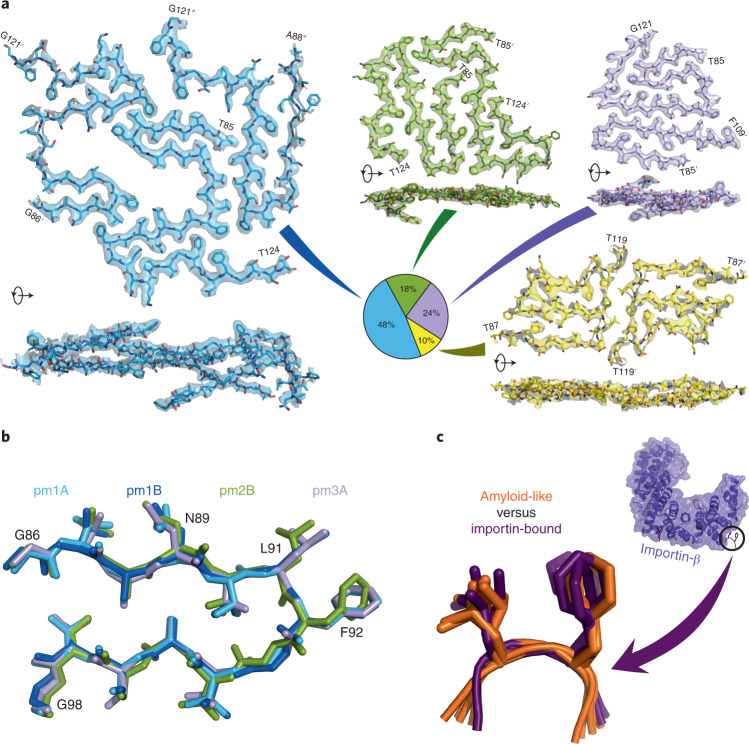
Cryo-EM structures of nucleoporin FG fibrils. **a**, Single cross-sections of the fibril structures of the Nup98^FG85^ polymorphs 1, 2, 3 and 4, shown in blue, green, violet and yellow, respectively, together with the cryo-EM density maps (from the top and side of the fibril axis). Zoned density maps are shown for clarity; unzoned maps are presented in Supplementary Figs. 6–9. Initial and final residues of each peptide are indicated, using ' and '' for the second and third chains, respectively. Polymorph populations, as a percentage of fibrillar segments, are displayed in the pie chart. The percentage of peptides forming each polymorph is 58, 15, 19 and 8% for pm1–pm4, respectively. **b**, The most common structural motif in Nup98^FG85^ fibrils: residues 86–98 of Nup98^FG85^ pm1 chain A (pm1A) and chain B (pm1B), pm2 chain B (pm2B) and pm3 chain A (pm3A) are aligned with a maximum root-mean-squared deviation of 0.492 Å. **c**, Superposition of the amyloid-like structures of six XLFX motifs, present in the Nup98^FG85^ pm1 fibrils (shown in **b**), on five GLFG motifs bound to importin-β (PDB ID: 1O6P).

To gain insight into the molecular organization of cohesive FG-repeat interactions, Nup98^FG85^ was allowed to aggregate in pure water. EM revealed the formation of well-defined and mostly separated amyloid fibrils (Supplementary Fig. 6). Using cryo-EM, we determined the structures of four different fibril polymorphs (pm1–pm4; Fig. 3a and Supplementary Tables 2 and 3).

The most abundant polymorph fibril, pm1, was resolved at a resolution of 2.8 Å (Supplementary Fig. 7a) and is formed by three asymmetrically arranged protofilaments (Supplementary Fig. 8). Polymorphs pm2 and pm3, which are almost equally populated, were solved at a resolution of 3.3 and 2.8 Å, respectively, and are formed by two protofilaments (Supplementary Figs. 7b,c, 9 and 10). The least populated polymorph, pm4, was reconstructed at a resolution of 3.4 Å (Supplementary Fig. 7d) and contains two protofilaments related by an approximate 2_1_ screw symmetry (Supplementary Fig. 11).

All four fibril types display a parallel in-register β-structure comprising short and kinked β-strands (Fig. 3a). Kinked aromatic-rich structures were previously named low-complexity aromatic-rich kinked segments^28^. The most common motif shared by the four polymorphs is a 13-residue β-turn/β-arch structure (Fig. 3b). A leucine-phenylalanine pair (L91-F92) is located at the tip of the β-turn, with both side chains pointing away from the turn (Fig. 3b). The other LF motifs in the four fibril structures show a very similar structure (Supplementary Fig. 12). Notably, this specific amyloid structure of the LF motif closely overlaps the structure of the GLFG motifs bound to importin-β (Fig. 3c).

### Cohesive FG–FG interactions at high resolution

**Fig. 4:**
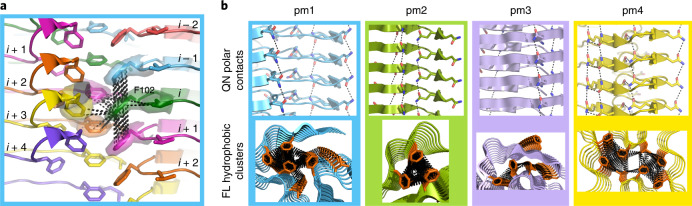
Cohesive FG–FG interactions at high resolution. **a**, Interactions of F102 with other phenylalanine residues from different chains and layers (shown in different colours) in pm1. The parameter *i* refers to the layer of the reference residue F102 (shown in green), *i* – 1 and *i* – 2 refer to the layers above the reference, and *i* + 1, *i* + 2, *i* + 3 and *i* + 4 to the layers below it. Associated cryo-EM densities are displayed. The dashed lines show F102 contacts below 5 Å. **b**, Selected views of glutamine-asparagine polar contacts (dashed lines, 4 Å cut-off; top) and major phenylalanine-rich clusters (5 Å cut-off; bottom) in pm1–4.

The structure of the most abundant polymorph, pm1, deviates considerably from the flat two-dimensional (2D) arrangement of cross-β-structure sheets (Fig. 4a). This leads to one chain forming contacts with several layers of the opposing protofilaments and the formation of a cluster of phenylalanine side chains (Fig. 4a,b, bottom). For example, the aromatic ring of F102 contacts three other phenylalanine rings located up to three layers away (Fig. 4a). Similar interactions can be found in the other polymorphs, where clusters of up to six phenylalanine rings, in some cases including leucine side chains, establish a tight network of molecular contacts (Fig. 4b, bottom). In all four Nup98^FG85^ fibril structures, side chains stacked along the fibril axes build glutamine and asparagine ladders (Fig. 4b, top). Previous studies showed that gels formed by Nup98 FG domains, which are rich in glutamine and asparagine, exhibit a strong amyloid-like character^5,27^.

**Fig. 5:**
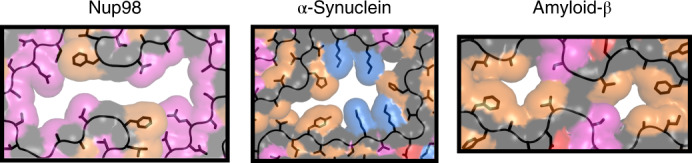
Polar residues line the cavity in nucleoporin FG fibrils. The cavity of Nup98^FG85^ fibril pm1 is compared with the cavities in the disease-associated fibrils of α-synuclein (PDB ID: 6XYO) and amyloid-β (PDB ID: 6SHS). Hydrophobic/aromatic residues (LIVWFYHMA) are shown in orange, polar residues (STQN) in magenta and charged residues in blue (KR) and red (ED), respectively.

The pm1 structure contains a large cavity (Fig. 3a) that is predominantly lined by polar residues (Fig. 5). Because the Nup98^FG85^ fibrils were prepared in pure water, it is likely that the cavity is filled with water molecules. In contrast, the cavity observed in the structure of α-synuclein fibrils purified from the brain of a patient with multiple system atrophy is lined by hydrophobic and charged residues (Fig. 5). Finally, mostly hydrophobic residues are observed at the rim of the cavity in amyloid-β fibrils purified from the brain of a patient with Alzheimer’s disease (Fig. 5). Hydrophobic and/or charged cofactors are therefore likely to fill the cavities of these disease-associated amyloid fibrils^29^.

### FG–FG interactions have low stability

**Fig. 6:**
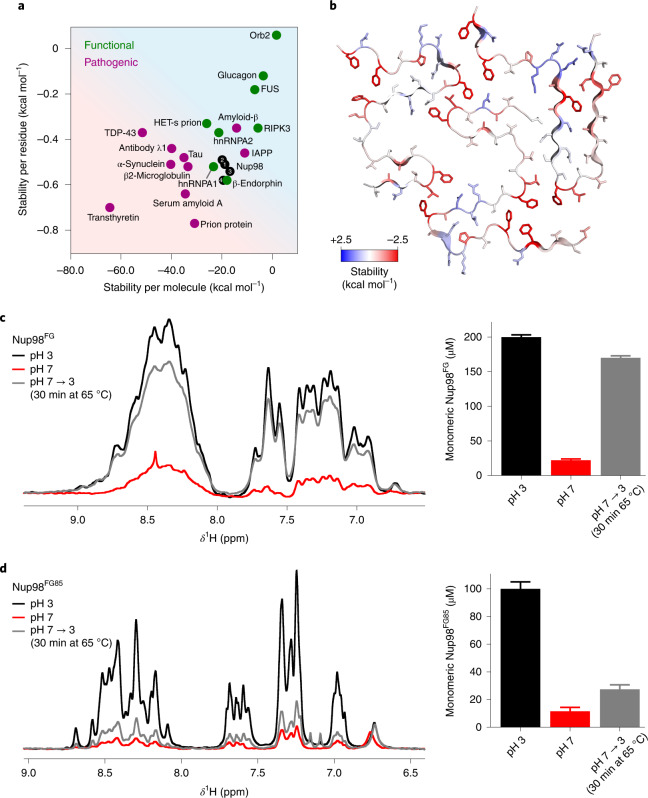
Cohesive FG clusters have low stability. **a**, Stability of Nup98^FG85^ fibrils (black) per residue and per molecule compared with the stability of previously determined functional and pathogenic fibril structures. The energies were calculated on the basis of cryo-EM structures. **b**, Residue-specific stabilities based on solvation energies mapped onto the structure of Nup98^FG85^ fibril pm1. Red and blue represent high and low stability, respectively. **c**, One-dimensional ^1^H NMR spectra of Nup98^FG^ at pH 3, after adjusting to pH 7 and after adjusting back to pH 3 and incubating the sample for 30 min at 65 °C (left). The corresponding concentrations of monomeric Nup98^FG^ calculated from the integral of the NMR signals (right). **d**, One-dimensional ^1^H NMR spectra of Nup98^FG85^ at pH 3, after adjusting to pH 7 and after adjusting back to pH 3 and incubating the sample for 30 min at 65 °C (left). The corresponding concentrations of monomeric Nup98^FG85^ calculated from the integral of the peaks (right). The error bars in **c** and **d** represent the s.d. based on NMR signal-to-noise ratios. Source data

Next, we estimated the atomic solvation energies^30^ as a measure of the stability of the four Nup98^FG85^ fibril structures and compared them with those of previously resolved fibril structures (Fig. 6a). We obtained values of approximately −20 kcal mol^–1^ per chain for the four Nup98^FG85^ fibril structures. This value is comparable to the stability of amyloid fibrils formed by the RNA-binding proteins hnRNPA1 and hnRNPA2 (Fig. 6a). By contrast, most disease-associated fibrils are predicted to be more stable (Fig. 6a). Considering that Nup98^FG85^ has the strongest condensation propensity in the FG-repeat domain of Nup98 (Fig. 2c), −20 kcal mol^–1^ per chain is likely to be the maximum value that is reached for cohesive conformations within the FG-repeat domain of Nup98.

Mapping of the calculated solvation energies onto the structures shows that the stabilizing interactions are distributed non-uniformly (Fig 6b and Supplementary Fig. 13). Most of the stabilizing interactions arise where there are clusters of two to four phenylalanine rings and one or more leucine side chains. Other regions contribute little to the stability of the FG nucleoporin fibrils (Fig. 6b and Supplementary Fig. 13).

The Nup98^FG85^ fibril structures are predicted to have higher stability than the amyloid fibrils formed by the low-complexity region of the stress granule-associated protein fused in sarcoma (FUS; Fig. 6a). The low-complexity region of FUS (FUS-LC) contains many polar residues (Supplementary Fig. 14). In addition, the predominant aromatic residue in FUS-LC is tyrosine, which has the ability to form polar contacts through its hydroxy group and does not form as many hydrophobic clusters as the phenylalanine residues in Nup98^FG85^ (compare Supplementary Fig. 14 with Fig. 6b). The high content of polar residues might be responsible for the predicted lower stability of FUS-LC fibrils (Fig. 6a).

### FG-based interactions are reversible

Pathogenic amyloid fibrils do not resolubilize when exposed to very high temperature (>100 °C) over several hours^31–33^. In contrast, functional fibrils/fibres associated with gels and membraneless organelles are less stable (Fig. 6a) and can dissociate upon changes in pH/ionic strength and elevated temperature (~65 °C)^31–33^. To experimentally study the stability of Nup98^FG^ aggregates, we freshly prepared the 384-residue FG-repeat domain of Nup98 at pH 3. In agreement with the CD and turbidity experiments (Fig. 1a,b), Nup98^FG^ at pH 3 displays an NMR spectrum that is typical of a flexible protein (Fig. 6c). When we then raised the pH to 7, we observed an immediate and strong signal loss. The signal loss was partially reversed on lowering the pH back to 3 and raising the temperature to 65 °C (Fig. 6c, left). In addition, the sample became less turbid (Fig. 1a). Quantification of the NMR signal intensities showed that ~85% of the original protein signal was regained (Fig. 6c, right).

We then performed the same experiments with the highly aggregation-prone Nup98^FG85^ segment (Fig. 6d). Again, strong signal loss occurred at native pH, but the NMR signal could be regained on returning to pH 3 and raising the temperature to 65 °C (Fig. 6d, left). Notably, however, only 22% of the original NMR signal was regained (Fig. 6d, right), indicating that the more aggregation-prone Nup98^FG85^ segment forms more stable aggregates. This combination of experiments (Fig. 6c,d) and stability estimations (Fig. 6a,b) demonstrates that amyloid-like aggregates of the FG-repeat domain of Nup98 have low stability.

## Discussion

The human nucleoporin Nup98 forms fusion proteins in certain types of malignant leukaemia, is a critical component of the permeability barrier of the nuclear pore complex and is mislocalized in cytosolic deposits in Alzheimer’s disease^2,9–12,14,15^. Key to these Nup98 activities is its large FG-repeat domain with a high number of FG motifs. In leukaemia-associated transcription factor condensates, the Nup98 FG-repeat domain is fused to DNA-binding domains, whereas it is attached to scaffold proteins inside the nuclear pore complex. In both cases, the FG-repeat domain of Nup98 critically influences the molecular properties of these cellular compartments. High-resolution information about the interactions between FG motifs either within a single Nup98^FG^ chain or between multiple Nup98^FG^ chains has, however, been largely elusive. In this study we combined NMR spectroscopy with four cryo-EM structures to gain detailed insights into the structural organization of both the more transient liquid-like and the more stable cohesive interactions of the FG-repeat domain of Nup98. The data reveal a preformed structure recognized by mRNA export factors and the molecular basis of FG cluster stabilization, as well as establishing a structural mimicry of FG motifs inside cohesive FG clusters and when bound to nuclear transport receptors.

The condensates formed by the FG-repeat domains of Nup98 from different species display different amounts of amyloid-like structure, that is, different levels of liquid- and solid-like properties^5^. Our data show that the ability of Nup98 to self-associate varies strongly along its long FG-repeat domain. The strongest self-association propensity is observed in 3 segments comprising ~30–40 residues (Fig. 2c). This suggests that Nup98 phase separation and condensate formation may be explained by a well-balanced network of interactions involving less cohesive and transient interactions as well as cohesive and stable interactions. Depending on the precise sequence composition, the relative contribution of liquid- and solid-like interactions is thus likely to vary in Nup98 FG-repeat domains from different species. The combination of NMR spectroscopy and cryo-EM provides insights into the least and most stable interactions from the spectrum of possible FG-repeat interactions. When more liquid-like interactions dominate, the FG-repeat domain is very dynamic, the FG motifs transiently populate β-structure and the GLEBS motif folds into a stable α-helix. Cryo-EM determines with high resolution the most stable interactions from the spectrum of possible FG-repeat interactions. The combined data thus advance our structural knowledge of both the local and long-range structure of cohesive FG interactions to a new level compared with previous studies (for example, ref. ^34^). Because the amyloid-like interactions between FG motifs are reversible (Fig. 6), it is likely that liquid-like transient and amyloid-like cohesive interactions are not completely distinct/disconnected molecular properties but can interchange even within a single region of an FG-repeat protein.

The central channel of the nuclear pore complex is filled with FG-repeat-containing nucleoporins that form the permeability barrier^6,7^. Several different (sometimes competing) models have been suggested for the molecular organization of the FG permeability barrier of the nuclear pore complex (reviewed, for example, in refs. ^7,16,35,36^). In some of these models, cohesive interactions between FG motifs do not play a role in the transport selectivity of the FG-filled channel, while in other models cohesive FG–FG interactions are critical. In particular, in a model that considers the FG-filled channel as a phase-separated biomolecular condensate, cohesive FG–FG interactions are essential^17,19^. In addition, it has been shown that self-assembled Nup98^FG^ particles, which display key features of transport selectivity, can contain cross-β-structure, as evidenced by ThT staining^5^. Indeed, we confirmed here the presence of cross-β-structure in the Nup98^FG^ particles by ThT staining (Fig. 1 and Supplementary Fig. 4). Solid-state NMR analysis of nucleoporin FG gels further indicated that regions containing asparagine and glutamine residues form the cross-β-structure^27^. Consistent with these results, we found that the most aggregation-prone segment of the FG-repeat domain of Nup98, identified in this study and structurally characterized at high resolution using cryo-EM, contains five asparagine and two glutamine residues.

Self-association and cohesive FG–FG interactions underlie the phase separation and formation of leukaemia-associated Nup98 fusion protein condensates^9,11,12^ and might influence the mislocalization and accumulation of Nup98 in Alzheimer’s disease^14^. However, high-resolution structural information has not been available for any of these FG-repeat assemblies. Furthermore, it has often been unclear whether they are more liquid- or solid-like, or whether they can be described at all by the physicochemical process of phase separation^7,9,14,16,35,36^. A recent study, for example, suggested that an artificial nucleoporin sequence based on Nup98 and comprising 52 repeats of 12 residues remains disordered in the phase-separated state^34^, while another study based on a solid-state NMR analysis identified an amyloid-like β-structure inside gels formed by the yeast nucleoporin Nsp1p (ref. ^27^). Importantly, changes in the material properties of condensates, from liquid- to solid-like, have been linked to human diseases^37^, suggesting that cohesive FG-repeat interactions could be most relevant for the pathological states of FG-repeat-based compartments and the mislocalization of FG-repeat-containing nucleoporins in neurodegenerative diseases.

In summary, our study represents a critical advance in the understanding of the molecular interactions determining the self-association of Nup98 and thus provides an important reference for future studies of FG-repeat proteins.

## Methods

### Protein preparation

The FG-repeat domain of human Nup98 (Nup98^FG^, residues 1–384) was cloned into bacterial expression vector pET28a (Novagen) between the restriction sites NheI and XhoI, keeping the N-terminal His-Tag for its purification. The recombinant Nup98^FG^ was expressed in the *Escherichia coli* expression strain BL21(DE3). For the unlabelled protein, the bacteria were grown in Luria-Bertani (LB) medium supplemented with kanamycin to an optical density at 600 nm (OD_600_) of ~0.8 and induced with 0.5 mM isopropyl-β-d-thiogalactoside (IPTG) overnight at 37 °C. For the uniform ^13^C,^15^N labelling of Nup98^FG^, 5 ml of an overnight LB culture was inoculated into 1 l of M9 medium containing 4 g l^–1^ d-[^13^C_6_]glucose and 2 g l^–1^ ^15^NH_4_Cl, supplemented with kanamycin and ^13^C,^15^N-labelled ISOGRO powder growth medium. At an OD_600_ of ~0.8, the culture was induced with 0.5 mM IPTG overnight at 37 °C. For purification, cell pellets were sonicated in lysis buffer containing 100 mM Na_2_HPO_4_, 10 mM Tris and 10 mM 2-mercaptoethanol at pH 8.5, and the lysate was clarified by centrifugation. After centrifugation, the supernatant was discarded and the pellet containing Nup98^FG^ was resuspended in denaturing buffer containing 8 M urea, 100 mM Na_2_HPO_4_, 10 mM Tris and 10 mM 2-mercaptoethanol at pH 8.5. After a second centrifugation step, the supernatant was loaded onto a self-packed nickel-nitrilotriacetic acid (Ni-NTA) column (Qiagen) equilibrated with denaturing buffer and bound protein was eluted with a buffer solution of 6 M urea, 100 mM Na_2_HPO_4_, 10 mM Tris and 10 mM 2-mercaptoethanol at pH 4.0. The same elution buffer was used to perform size exclusion chromatography on a Superdex75 26/600 column (GE Healthcare). A second size exclusion chromatography (using the same column equilibrated with 50 mM sodium phosphate buffer containing 1 mM tris(2-carboxyethyl)phosphin (TCEP) at pH 3) was conducted to remove any remaining impurities. The pure protein was concentrated by ultracentrifugation using a 5 kDa molecular weight cut-off membrane.

Nup98 peptides were prepared by solid-phase synthesis (Genscript). For EM and optical microscopy measurements, powdered lyophilized Nup98^FG85^ was dissolved in pure water to reach a concentration of 1.5 mM, followed by incubation at 25 °C for 1 h.

### Circular dichroism

Nup98^FG^ samples were prepared at a concentration of 5 μM in 50 mM sodium phosphate buffer containing 1 mM TCEP at pH 3. The experiments at pH 7 were performed with the same samples, adjusting the pH to 7 just before measurement. CD data were collected in the range 185–280 nm using a Chirascan-plus qCD spectrometer (Applied Photophysics) at 25 °C with 1.5 s per point in 1 nm steps. The datasets are averages of ten repeat experiments. All spectra were baseline-corrected against buffer and smoothed (window size: 4) using GraphPad Prism.

### Dynamic light scattering

Nup98^FG^ samples were prepared at a concentration of 2.5 μM in 50 mM sodium phosphate buffer containing 1 mM TCEP at pH 3. Samples at pH 7 were prepared in the same buffer by adjusting the pH to 7 just before measurement and reducing the protein concentration to 100 nM. Both concentrations were optimized to avoid saturation of the detector. Measurements were conducted at 25 °C using a DynaPro NanoStar instrument (Wyatt Technologies) and NanoStar disposable microcuvettes. The samples were illuminated with a 120 mW air-launched laser at a wavelength of 662 nm and the intensity of light scattered at an angle of 90° was detected with an actively quenched, solid-state single-photon counting module. Data were acquired with an acquisition time of 5 s with a total of five acquisitions per measurement. The hydrodynamic radii were determined using the Dynamics (version 7.10.0.23) software package. The final values are given as the average and standard error of 12 measurements.

### Light microscopy

For optical microscopy, Nup98^FG^ samples were prepared at a concentration of 50 μM in 50 mM sodium phosphate buffer containing 1 mM TCEP at pH 3. The samples measured at pH 7 were prepared by adjusting the pH to 7 just before measurement. Nup98^FG85^ samples were prepared by dissolving the powder in water to a concentration of 1.5 mM, incubating them for 1 h at 25 °C and then diluting to a concentration of 200 μM. Where indicated, ThT was added to reach a concentration of 50 μM. A total 5 μl of sample was loaded onto a slide and covered with a 18 mm coverslip. Differential interference contrast micrographs as well as fluorescent micrographs were acquired on a Leica DM6B microscope with a ×63 objective (water immersion) and processed using ImageJ. The exposure time of the fluorescent micrographs in the absence of ThT was 100 times longer.

FRAP measurements of Nup98^FG85^ hydrogels were conducted with a Leica TCS SP8 confocal microscope using a ×63 objective (oil immersion) and a 488 nm argon laser line. Samples were loaded onto a slide and covered with a 18 mm coverslip. FRAP curves were acquired in 64 × 64 format at a scan speed of 1,000 Hz. Ten frames were collected for pre-bleaching and bleaching, and 500 frames for post-bleaching. Each frame corresponded to 523 ms. Regions of interest (ROIs) were bleached with 80% laser power, while a low laser intensity (5%) was used during recovery. Data were processed using FIJI software (NIH).

FRAP recovery curves were obtained by the standard protocols. Briefly, for each FRAP measurement, the intensities of the pre-bleaching, bleaching and post-bleaching ROIs were measured. A pre-bleaching ROI corresponds to a selected region in the droplet before bleaching, a bleaching ROI corresponds to the bleached area, and a reference ROI corresponds to an area that did not experience bleaching. The fluorescence intensity measured for each of the described ROIs was corrected for background by subtraction; a region where no fluorescence was detected was used to calculate the background.

### NMR spectroscopy

NMR spectra were recorded at 5 °C on Bruker 700, 800, 900 and 950 MHz spectrometers equipped with triple-resonance cryogenic probes. For the full-length Nup98^FG^ protein (200 μM in 50 mM sodium phosphate buffer, 1 mM TCEP, 0.01% NaN_3_, 5% D_2_O, pH 3), one-dimensional ^1^H NMR and 2D ^1^H–^1^H TOCSY spectra were acquired at 800 MHz. In addition, ^1^H–^15^N and ^1^H–^13^C heteronuclear single quantum coherence (HSQC) and three-dimensional (3D) HNCO, HNcaCO, HNCA, HNcoCA, HNCACB and HNcoCACB spectra were recorded at 900 MHz. Samples were incubated each day for 30 min at 65 °C to resolubilize the protein.

The ^1^H–^15^N HSQC spectrum at pH 7 was acquired after adjusting the pH of the same sample from 3 to 7 (the dead time from changing the pH, mixing, transferring to the NMR spectrometer to starting the ^1^H–^15^N HSQC acquisition was ~5 min). The acquisition time of the^1^H–^15^N HSQC spectrum at pH 7 was 15 min.

To assign the backbone resonances of the FG-repeat domain of Nup98 at pH 6.8, 17 overlapping Nup98 peptides (N-terminal acetylated, except residues 1–15, and carboxy-terminal amidated, except residues 5–44) were used covering the sequence of residues 1–384: 1–15, 5–44, 30–59, 45–84, 72–99, 95–124, 115–144, 137–170, 165–204, 189–218, 205–244, 225–264, 256–275, 265–304, 298–327, 322–351 and 345–384. Peptide concentrations of 2 mM were used for resonance assignment. The peptides were dissolved in 50 mM sodium phosphate buffer containing 0.01% NaN_3_ and 5% D_2_O at pH 6.8. Samples of Nup98(165–204) also included 1 mM TCEP. Several samples of Nup98(95–124), Nup98(298–327) and Nup98(345–384) were used due to their rapid aggregation. One-dimensional ^1^H NMR, 2D ^1^H–^1^H TOCSY, NOESY, and ^1^H–^15^N and ^1^H–^13^C HSQC spectra of the 17 overlapped Nup98 peptides were acquired. All the NMR data were processed using TopSpin 3.6.1 (Bruker) and analysed with Sparky^38^. The ^1^H NMR chemical shifts were referenced to 2,2-dimethyl-2-silapentane-5-sulfonate (DSS, 0 ppm), and the ^13^C and ^15^N NMR chemical shifts were indirectly referenced to DSS. All HA, HN, N, CA and CB resonances were assigned except for the N of prolines and the CA of the three asparagine residues before prolines. The overlapping peptide assignments were used to negate the influence of peptide ends on the final chemical shift assignments (Supplementary Fig. 15). The secondary structure and *φ*/*ψ* angles were calculated from the experimental HA, HN, N, CA and CB chemical shifts using TALOS-N (ref. ^39^).

To study the reversibility of the amyloid-like structure in the FG-repeat domain of Nup98, samples of Nup98^FG^ (200 μM) and Nup98^FG85^ (100 μM) were prepared in 50 mM sodium phosphate buffer containing 1 mM TCEP, 0.01% NaN_3_ and 5% D_2_O at pH 3. Subsequently, the pH was adjusted to 7 and then back to 3, followed by incubation of the samples for 30 min at 65 °C in a water bath.

### Transmission electron microscopy

Nup98^FG^ samples at a concentration of 20 μM were dialysed in 50 mM HEPES buffer containing 1 mM TCEP at pH 3, and just before preparation of the grid, the pH was adjusted to pH 7 by adding NaOH. Samples were adsorbed onto 400-mesh carbon-coated copper grids and the buffer removed using filter paper. Subsequently, samples were stained by the addition of 1% uranyl acetate solution and dried with filter paper. The grids were imaged using a Talos L120C G2 electron microscope.

### Cryoelectron microscopy

Nup98^FG85^ fibrils were prepared by dissolving the peptide in water to reach a concentration of 1.5 mM, followed by incubation at 25 °C for 1 h. Subsequently, 3 μl of sample was applied to freshly glow-discharged R2/1 holey carbon grids (Quantifoil) and vitrified in liquid ethane using a Mark IV Vitrobot (Thermo Fisher) operated at 100% relative humidity and 4 °C. Cryoelectron microscopy was conducted with a Titan Krios transmission electron microscope (Thermo Fisher) operated at an accelerating voltage of 300 keV. Images were recorded at a nominal magnification of ×81,000 using a Quantum LS energy filter (Gatan) with the slit width set to 20 eV and a K3 direct electron detector (Gatan) in non-super-resolution counting mode, corresponding to a calibrated pixel size of 1.05 Å at the specimen level. In total, 4,180 images with defocus values in the range of –0.7 to –2.0 μm were acquired in movie mode with an acquisition time of 2.5 s. Each movie contained 40 frames with an accumulated dose of approximately 41 electrons per Å^2^. The resulting dose-fractionated image stacks, containing all 40 frames, were subjected to beam-induced motion correction on the fly using Warp^40^.

Nup98^FG85^ fibrils were reconstructed using RELION-3.1 (ref. ^41^) following the helical reconstruction scheme^42^. First, contrast transfer function (CTF) parameters were estimated for each motion-corrected micrograph using CTFFIND4 (ref. ^43^), and only micrographs with an estimated resolution of ≤4.0 Å were considered for manual fibril picking. For 2D classification, we extracted particle segments using a box size of 600 pixels downscaled to 200 pixels and an interbox distance of 14 Å; pm1 and pm3 fibrils were successfully separated by this classification procedure, but pm2 and pm4 could not be separated owing to their close similarity. For 3D classification, the segments after 2D classification were re-extracted without downscaling using a box size of 250 pixels. The initial helical rise was estimated from the cross-over distances (180° helical turn), measured from the 2D class averages, and the helical rise was initially set to 4.75 Å. We performed the 3D classification several times, starting from a 60 Å low-pass-filtered featureless cylinder, until the separated β-strands along the helical axis became visible, and then optimized the helical parameters (the final parameters are reported in Supplementary Table 2). We were able to separate the pm2 and pm4 fibrils during the 3D classification. For 3D auto-refinement, all fibril polymorphs were reconstructed individually. Next, standard RELION post-processing with a soft-edged solvent mask that included the central 10% of the box height yielded post-processed maps (sharpening *B* factors are reported in Supplementary Table 2). The resolution was estimated from the value of the Fourier shell correlation (FSC) curve for two independently refined half maps at 0.143 (refs. ^44,45^; Supplementary Fig. 7). Finally, the optimized helical symmetry was applied to the post-processed maps to yield the final maps.

The atomic models of the Nup98^FG85^ fibrils were constructed de novo in Coot^46^. The high resolution of the cryo-EM maps allowed reliable modelling of the protein backbone and side chain rotamers. Refinement in real space was conducted using PHENIX^47,48^ and Coot^46^ in an iterative manner. The resulting models were validated with MolProbity^49^ and their construction data are presented in Supplementary Table 3.

### Solvation energy calculation

Nup98^FG85^ fibril stability was calculated on the basis of the solvation energy using the software accessiblesurfacearea_v07.2d (ref. ^30^). The number of layers for each fibril was set to five, and the energy of the middle layer was used. For pm1, nine layers were used because of the interactions between distant layers.

### Reporting summary

Further information on research design is available in the Nature Research Reporting Summary linked to this article.

## Online content

Any methods, additional references, Nature Research reporting summaries, source data, extended data, supplementary information, acknowledgements, peer review information; details of author contributions and competing interests; and statements of data and code availability are available at 10.1038/s41557-022-01035-7.

## Supplementary information

Supplementary Information Supplementary Figs. 1–15 and Tables 1–3.

Reporting Summary

Supplementary Data Numerical source data file for Supplementary Figs. 1–15.

## Data Availability

Data supporting the main findings of this study are available in the main text and the Supplementary Information. NMR chemical shifts are included in the Supplementary Information. Cryo-EM maps have been deposited in the Electron Microscopy Data Bank (EMDB) under the accession numbers EMD-13851 (pm1), EMD-13852 (pm2), EMD-13853 (pm3) and EMD-13854 (pm4). The corresponding atomic models have been deposited in the PDB under the accession numbers: 7Q64 (pm1), 7Q65 (pm2), 7Q66 (pm3) and 7Q67 (pm4). PDB accession codes 6XYO, 6IC3, 6SHS, 6NZN, 6VPS, 6SDZ, 6ZRF, 6XFM, 6MST, 6GK3, 7KWZ, 5O3O, 6TUB, 2RNM, 7BX7, 6WQK, 6UUR and 5V7Z cited in the text are publicly available in the PDB. Source data are provided with this paper.

## Source data

Source Data Fig. 1 CD data and secondary structure propensity data.
Source Data Fig. 2 Peptide aggregation data and FRAP data.
Source Data Fig. 6 Stability calculations and soluble protein concentration data.
