# Supplementary material for: Molecular interactions of FG nucleoporin repeats at high resolution
Source: Nat Chem. 2022 Sep 22;14(11):1278–85. doi: 10.1038/s41557-022-01035-7 (PMC9630130; doi:10.1038/s41557-022-01035-7)
Supplement: Supplementary file 1 — Supplementary Figs. 1–15 and Tables 1–3. [file 41557_2022_1035_MOESM1_ESM.pdf]

nature portfolio

https://doi.org/10.1038/s41557-022-01035-7

Supplementary information

Molecular interactions of FG nucleoporin repeats at high resolution

In the format provided by the
authors and unedited

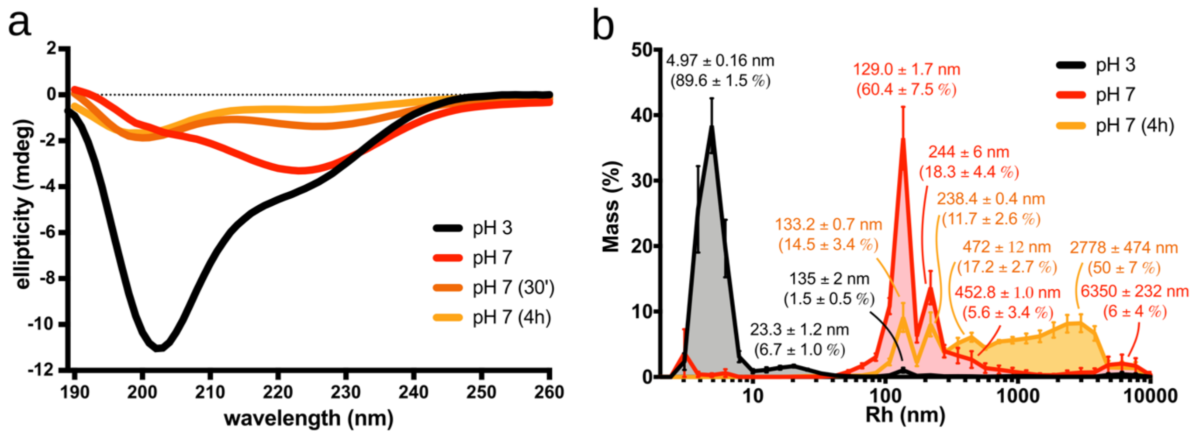

Fig. S1 |

Biophysical properties of the FG-repeat domain of human Nup98. a, Circular dichroism spectra at pH 3 (black) and 7 (red), and the evolution over time (orange, 30' and light orange, 4 h). **b,** Hydrodynamic radii of Nup98^{FG} measured by dynamic light scattering at pH 3 (black) and 7 (red), and the evolution over time (light orange, 4 h). The sizes, together with the population percentages in mass and the respective standard errors from 12 measurements are displayed next to each peak.

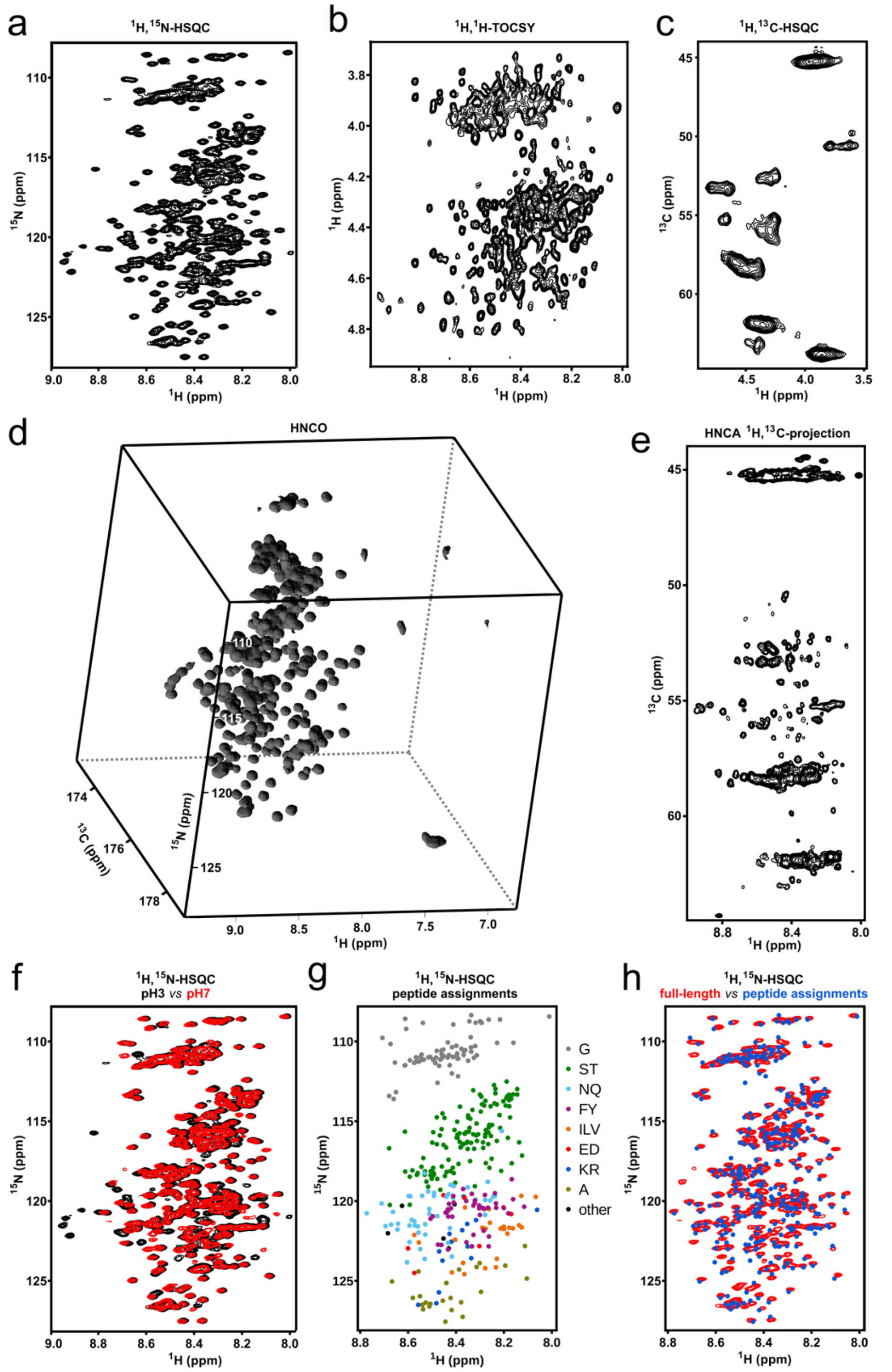

Fig. S2 |

NMR spectroscopy of the FG-repeat domain of human Nup98. a-c, ^{1}H,^{15}N-HSQC (a), ^{1}H,^{1}H-TOCSY (b) and ^{1}H,^{13}C-HSQC (c) spectrum of Nup98^{FG} at pH 3. **d,** 3D HNCO spectrum of Nup98^{FG} at pH 3. **e,** Projection of the ^{1}H,^{13}C planes of the 3D HNCA spectrum of Nup98^{FG} at pH 3. **f,** Comparison of the ^{1}H,^{15}N-HSQC spectra of Nup98^{FG} at pH 3 (black; same as in (a)) and pH 7 (red). **g,** ^{1}H,^{15}N-correlation spectrum that was generated from the chemical shifts, which were obtained through the assignment of the 17 peptides at pH 6.8. Each type of residue is colored as indicated. **h,** Same correlation spectrum as in (g; now in blue) over the ^{1}H,^{15}N-HSQC spectrum of Nup98^{FG} measured at pH 7 before signal decay due to gel/aggregate formation.

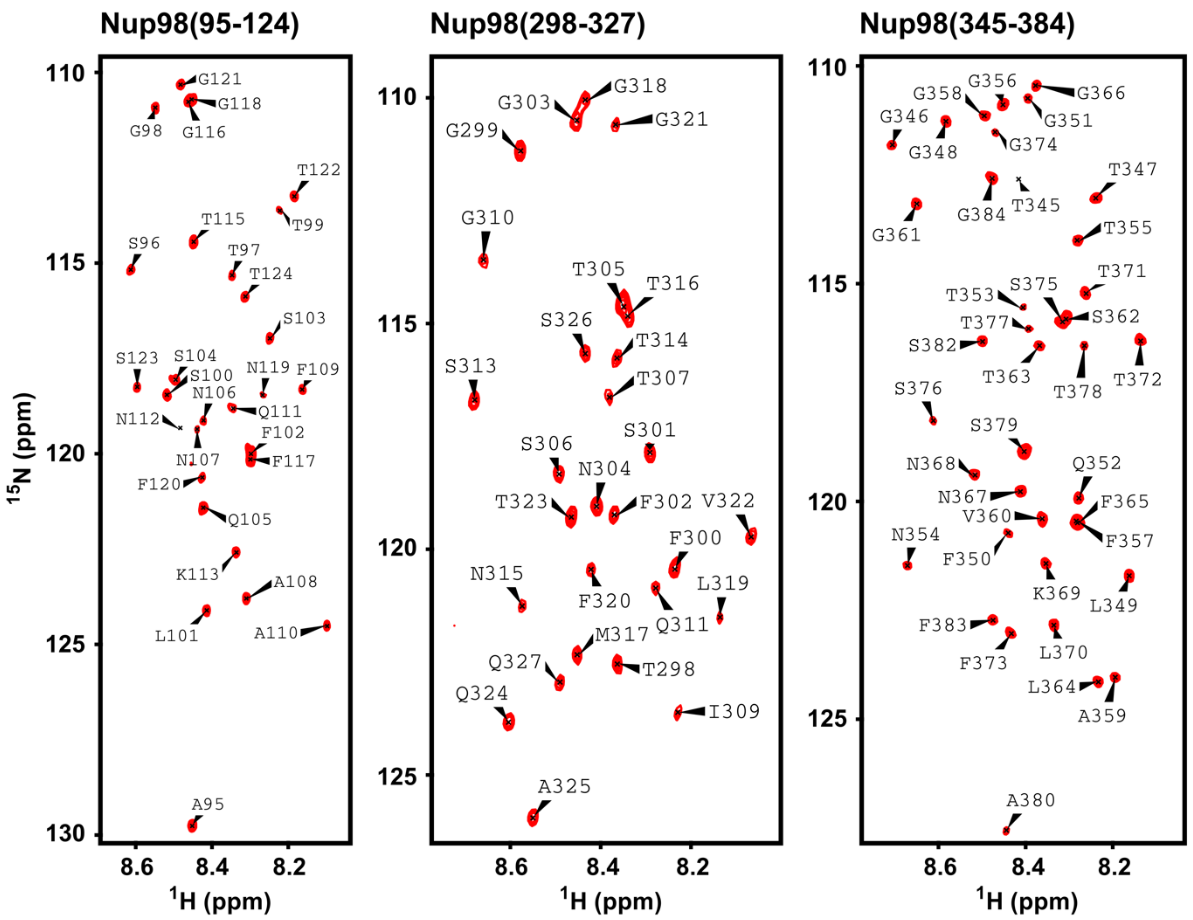

Fig. S3 |

NMR spectroscopy of peptides from the FG-repeat domain of Nup98. Two-dimensional ^{1}H-^{15}N HSQC spectra of three of the Nup98 peptides used for the assignment of the 384-residue Nup98^{FG} protein. Samples were prepared at 2 mM peptide concentration in sodium phosphate buffer, pH 6.8 and measured at 278 K.

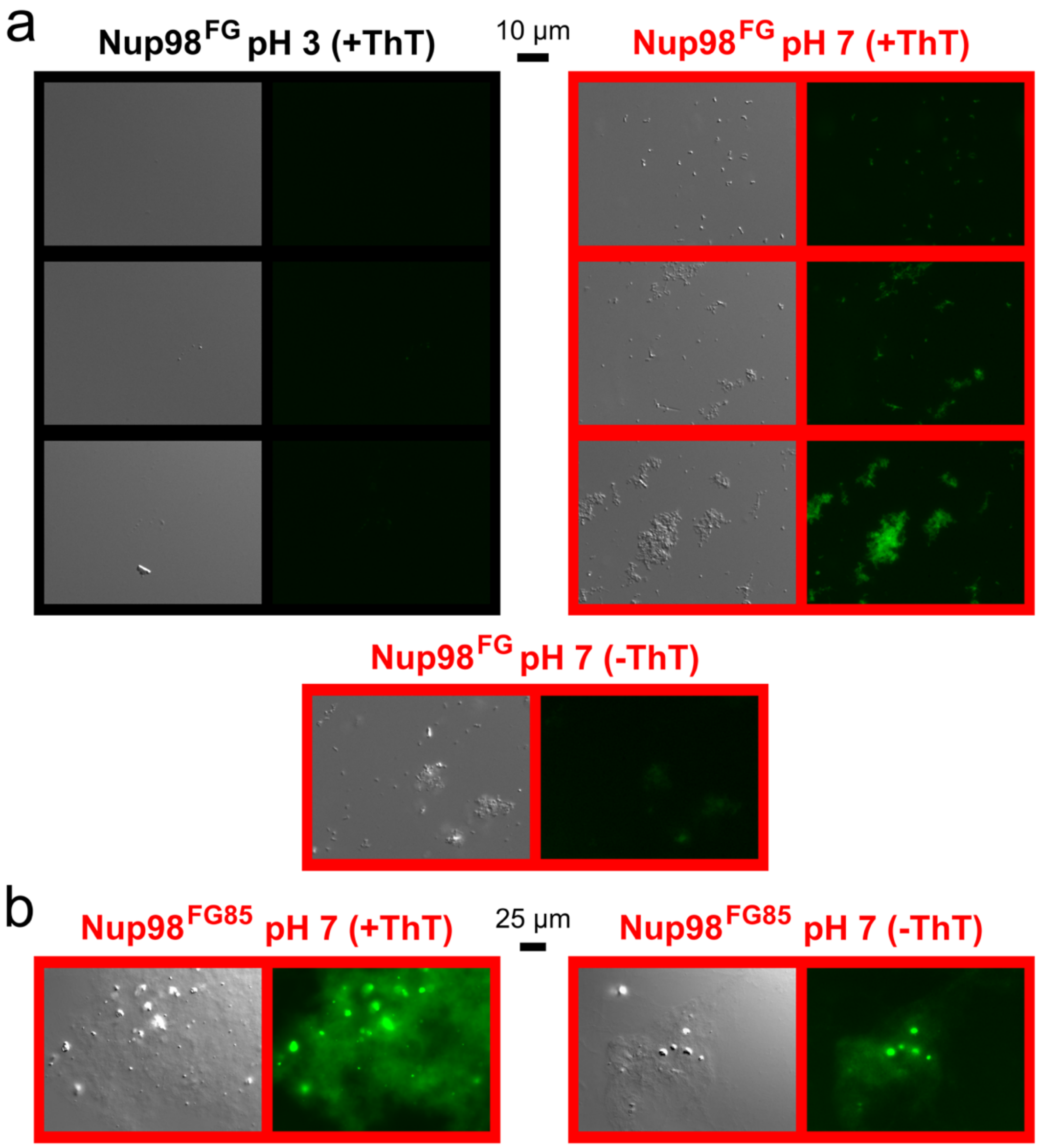

Fig. S4 |

The FG-repeat domain of human Nup98 forms cross-β structure. a, Differential interference contrast (left of each panel) and fluorescence (right of each panel) microscopy of Nup98^{FG} at pH 3 (top left, black) and pH 7 (top right, red) in the presence of ThT, and at pH 7 in the absence of ThT (bottom, red) displaying autofluorescence typical of amyloid-like structures [1]. Zooms of these micrographs are used in Fig. 1G. **b,** Nup98^{FG85} differential interference contrast (left of each panel) and fluorescence (right of each panel) microscopy at pH 7 in the presence (left) and absence (right) of ThT. For autofluorescence micrographs, 100 times-longer exposure times were used.

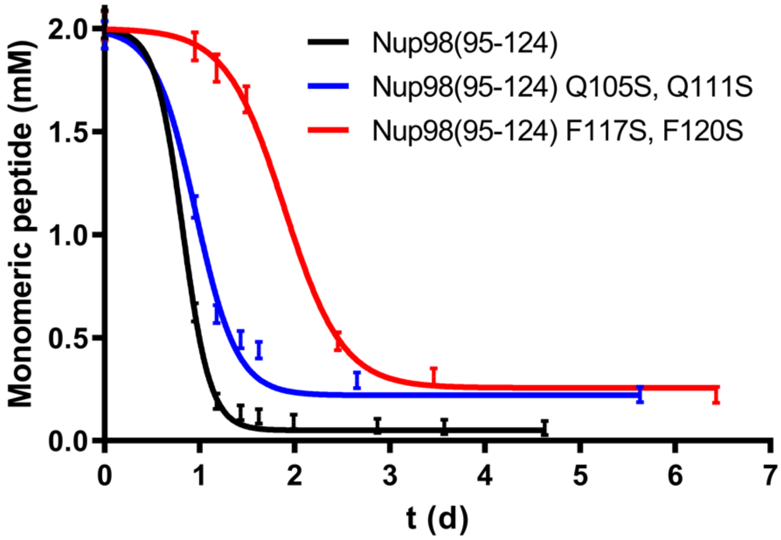

Fig. S5 |

Impact of site-directed mutagenesis on self-association kinetics of Nup98(95-124). Time-dependent decrease in the concentration of NMR-observable Nup98(95-124) (black) and its two variants “Nup98(95-124) Q105S, Q111S” (blue) and “Nup98(95-124) F117S, F120S” (red). The concentrations are calculated from the integral of the 1D ^{1}H NMR peaks and the error bars represent std based on NMR signal-to-noise ratios. The experiments were acquired at 278 K with 2 mM peptide concentration, in sodium phosphate buffer, pH 6.8.

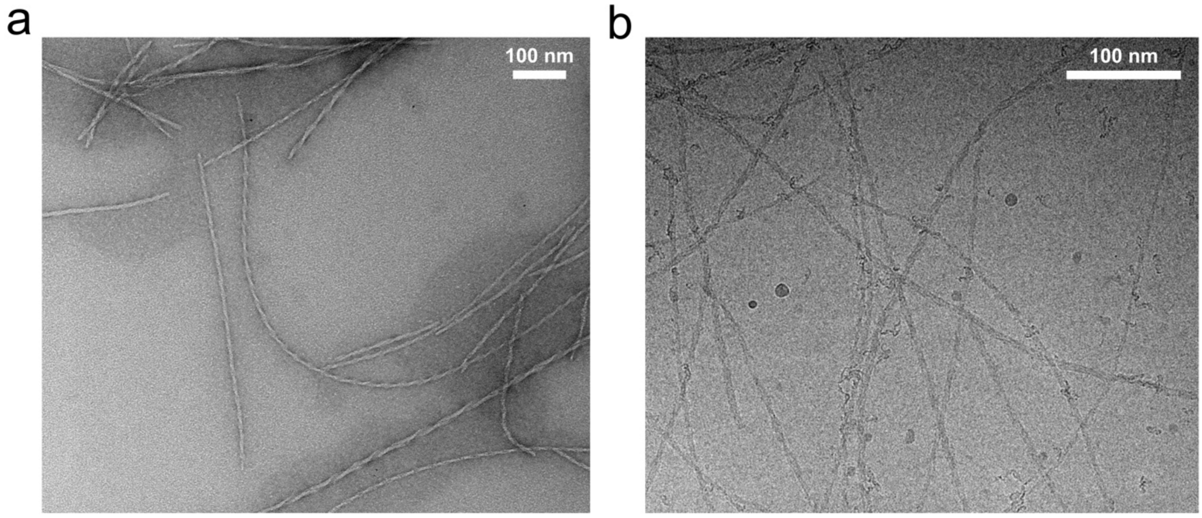

Fig. S6 |

Electron microscopy of Nup98^{FG85} fibrils. a, Negative-stain electron micrograph. **b**, Cryo-electron micrograph.

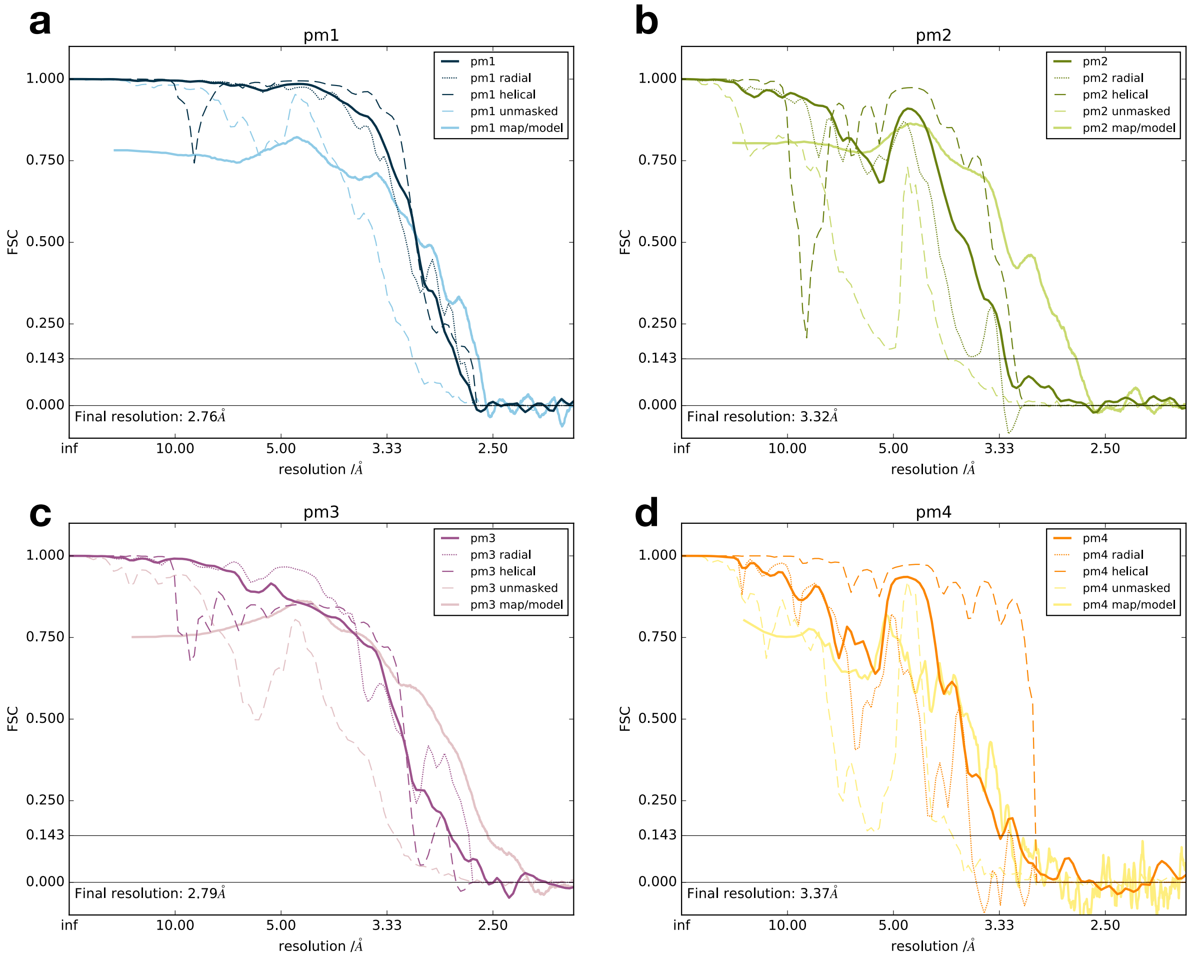

Fig. S7 |

Fourier shell correlation curves. Fourier shell correlation curves (FSC) for pm1 (a), pm2 (b), pm3 (c), and pm4 (d). The FSC curves shown between two independently refined masked (dark solid lines) and unmasked (light dashed lines) half-maps. For the masked FSC, a further calculation was made as to the contribution of the radial (dotted) and helical (dashed) directions, using 3DFSC [2]. Also shown is the map/model FSC calculated with Phenix Mtriage (light solid line) [3], which yield model resolution values (for FSC=0.5) of 3.02 Å, 3.27 Å, 2.88 Å, 3.71 Å for pm1, pm2, pm3, and pm4, respectively. The z-percentage is 0.1 in all cases. The final resolution is shown in the plot and was estimated from the value of the FSC curve for two independently refined masked half-maps at 0.143.

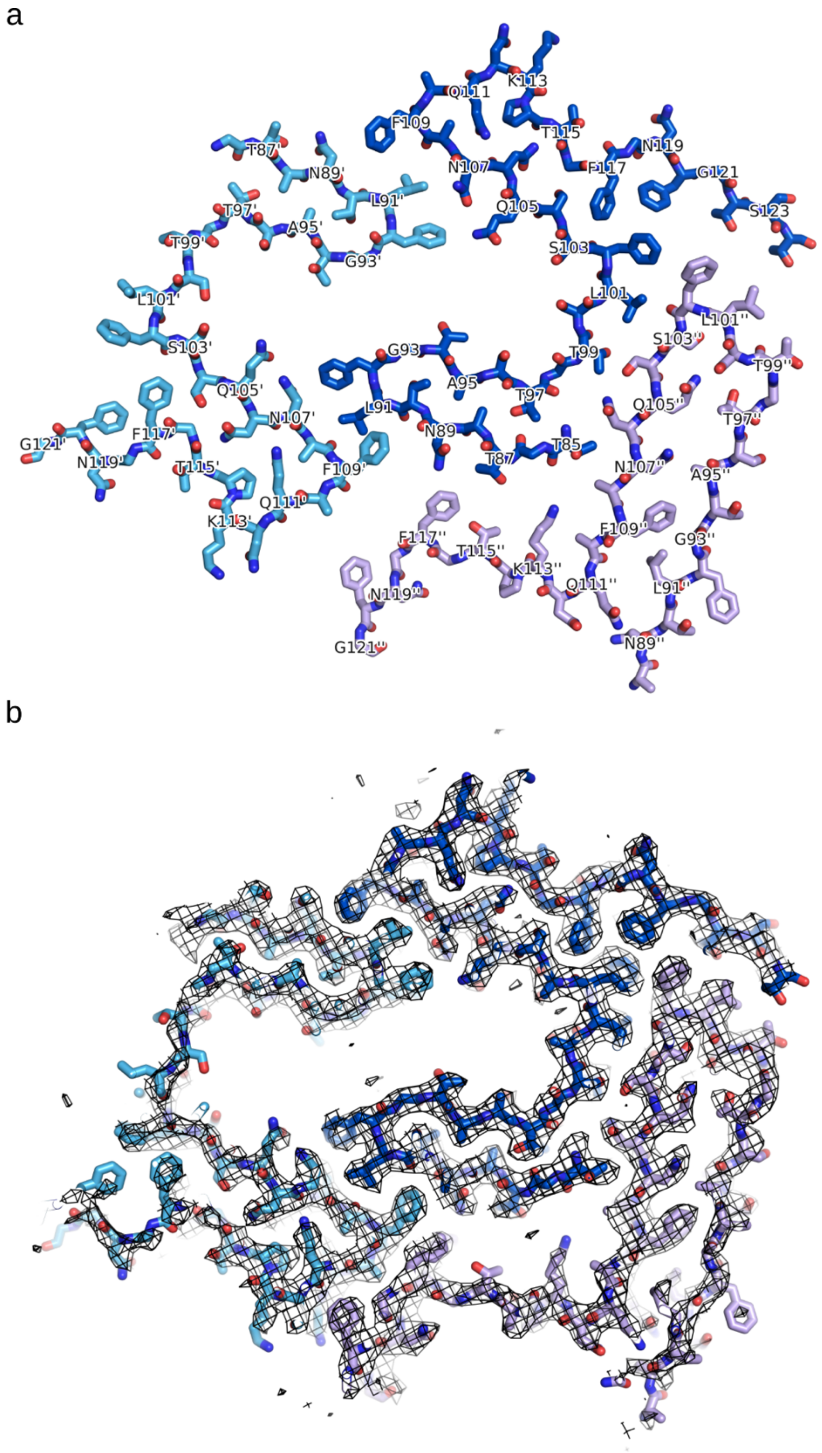

Fig. S8 |

Cryo-EM map and atomic model of the Nup98^{FG85} fibril polymorph 1 (pm1). a, Single cross-section of the Nup98^{FG85} fibril polymorph 1 in stick representation. The protofilaments are colored in different shades of blue. Residue identifiers are shown for every second amino acid. Amino acids from the light-colored protofilaments are labeled with an additional prime or double prime. **b,** Superposition of the atomic model and the final cryo-EM map (black isomesh; contour level of 0.05).

a

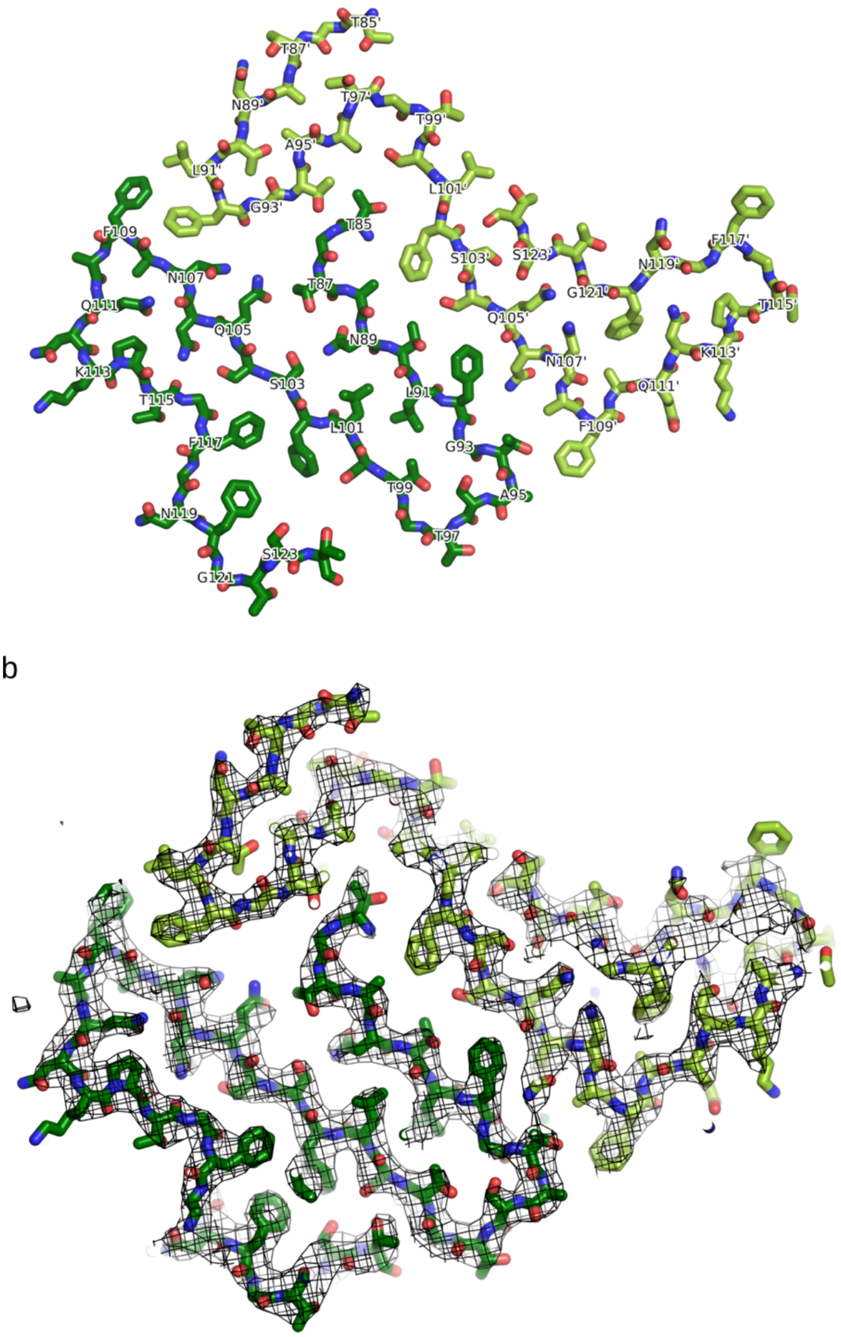

Fig. S9 |

Cryo-EM map and atomic model of the Nup98^{FG85} fibril polymorph 2 (pm2). a, Single cross-section of the Nup98^{FG85} fibril polymorph 2 in stick representation. The protofilaments are colored in different shades of green. Residue identifiers are shown for every second amino acid. Amino acids from the light-colored protofilaments are labeled with an additional prime or double prime. **b,** Superposition of the atomic model and the final cryo-EM map (black isomesh; contour level of 0.05).

a

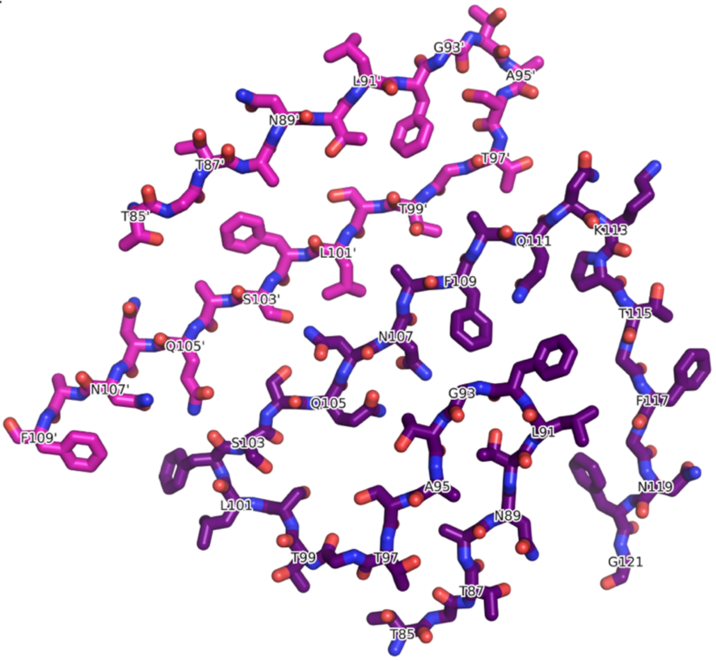

b

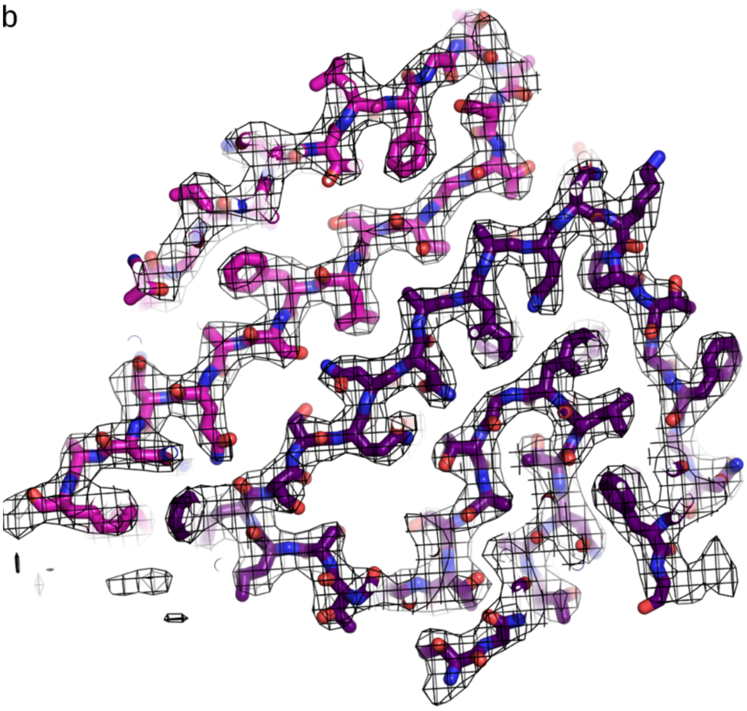

Fig. S10 |

Cryo-EM map and atomic model of the Nup98^{FG85} fibril polymorph 3 (pm3). a, Single cross-section of the Nup98^{FG85} fibril polymorph 3 in stick representation. The protofilaments are colored in different shades of violet. Residue identifiers are shown for every second amino acid. Amino acids from the light-colored protofilaments are labeled with an additional prime or double prime. **b,** Superposition of the atomic model and the final cryo-EM map (black isomesh; contour level of 0.05).

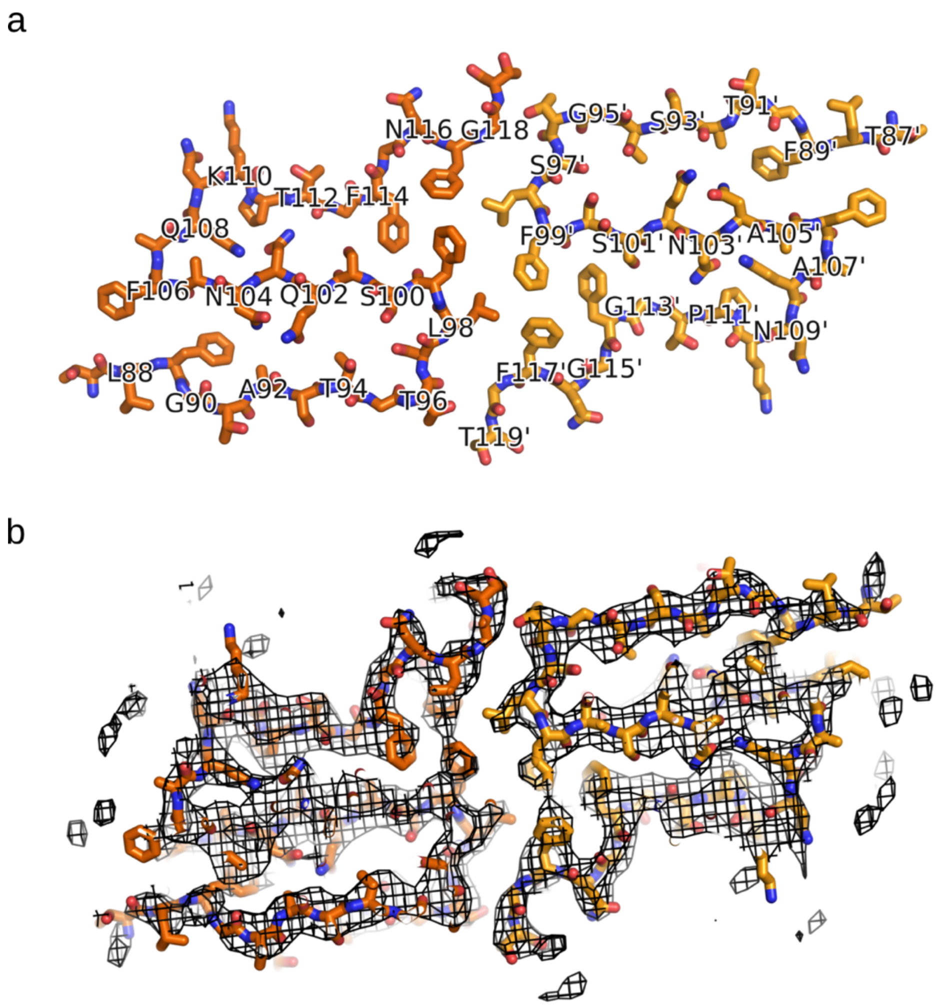

Fig. S11 |

Cryo-EM map and atomic model of the Nup98^{FG85} fibril polymorph 4 (pm4). a, Single cross-section of the Nup98^{FG85} fibril polymorph 4 in stick representation. The protofilaments are colored in different shades of orange. Residue identifiers are shown for every second amino acid. Amino acids from the light-colored protofilaments are labeled with an additional prime or double prime. **b,** Superposition of the atomic model and the final cryo-EM map (black isomesh; contour level of 0.04).

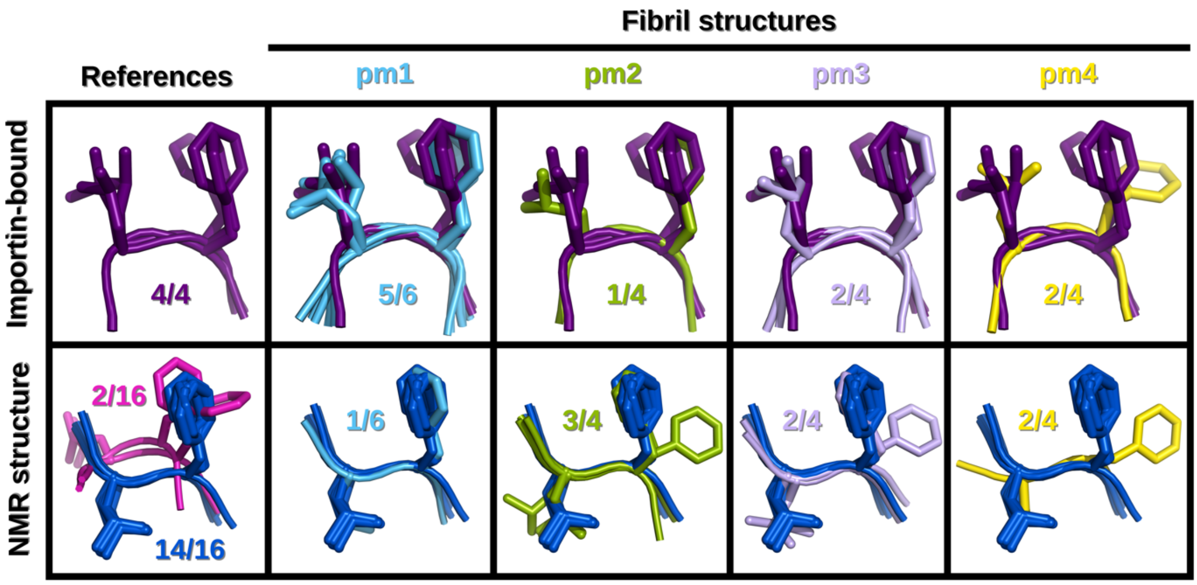

Fig. S12 |

Structures of xLFx motifs of Nup98^{FG85} fibrils. For each polymorph (pm1-pm4; different columns), the structures of their xLFx motifs are compared with the importin-bound GLFG structures (PDB id: 1O6P, purple, top row) and with the TALOS-N calculated structures from NMR data (blue, bottom row). The reference structures are represented alone in the left column. The two NMR-derived structures that have a different conformation are shown in magenta (bottom row; left). The number of structures of each polymorph represented from the total of motifs of that polymorph are displayed next to the structures.

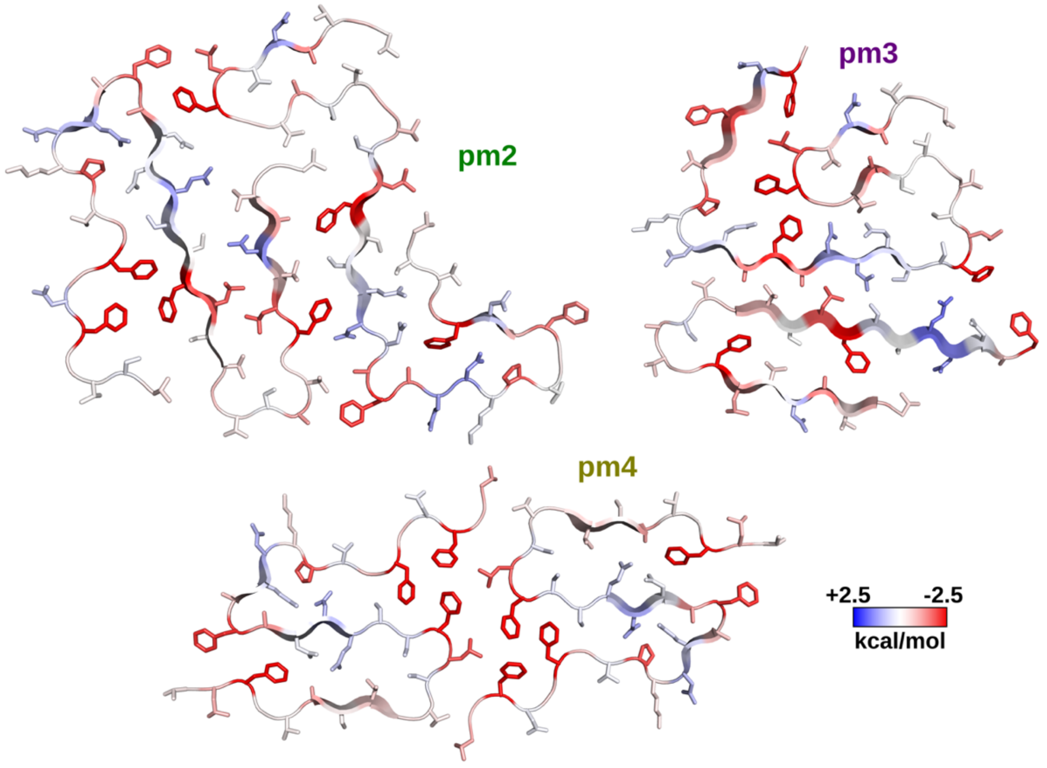

Fig. S13 |

Solvation energy stabilities of the fibril polymorphs 2, 3 and 4 of Nup98^{FG85}. Residue-specific stabilities based on solvation energies mapped onto the structures of the Nup98^{FG85} polymorphs 2, 3 and 4 (pm2-pm4). Red and blue represent high and low stability, respectively.

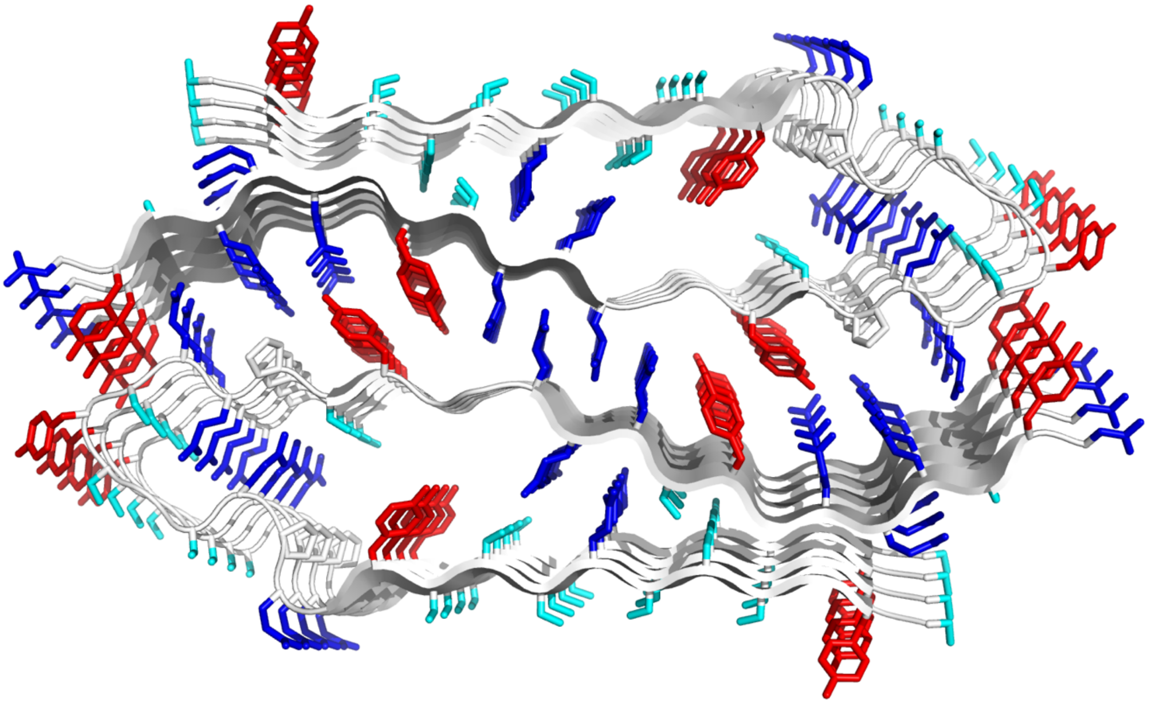

Fig. S14 │

CryoEM structure of FUS-LC fibrils (PDB: 6XFM). Tyrosine side chains are colored in red and polar residues in blue (glutamine and asparagine) and cyan (serine and threonine).

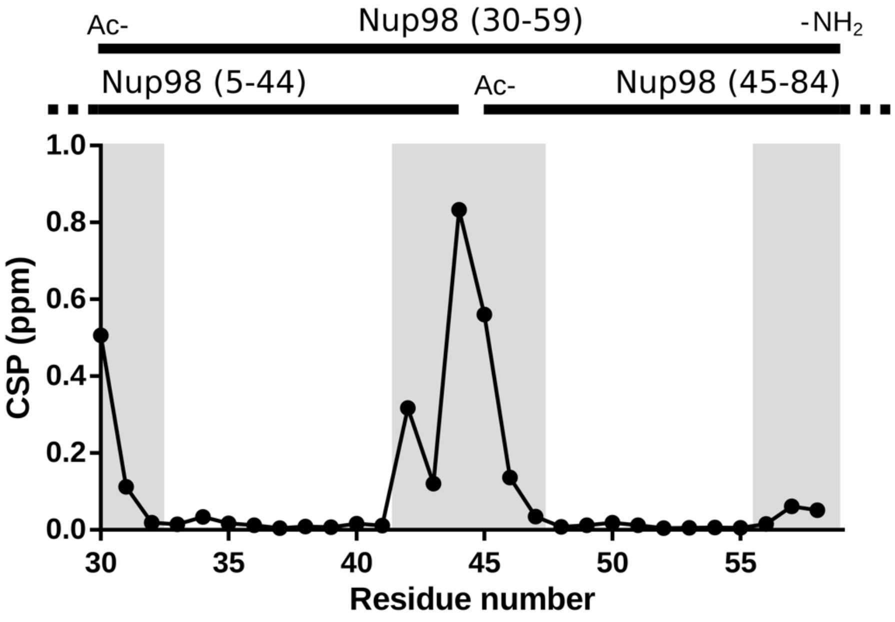

Fig. S15 |

Chemical shift selection from peptide assignments. Normalized and averaged ^{1}H-^{15}N chemical shift differences between overlapped peptides. Differences are restricted to the 2-3 terminal residues, which were excluded from the selected chemical shifts for the full Nup98^{FG} assignment.

Supplementary References

1 Grelich-Mucha, M. *et al.* Autofluorescence of Amyloids Determined by Enantiomeric Composition of Peptides. *J Phys Chem B* **125**, 5502-5510 (2021).

2 Tan, Y. Z. *et al.* Addressing preferred specimen orientation in single-particle cryo-EM through tilting. *Nat Methods* **14**, 793-796 (2017).

3 Afonine, P. V. *et al.* New tools for the analysis and validation of cryo-EM maps and atomic models. *Acta Crystallogr D Struct Biol* **74**, 814-840 (2018).

Table S1 │ Nuclear magnetic resonance chemical shift assignments of the 17 peptides of Nup98^{FG} and the full 1-384 Nup98^{FG}. For each peptide the fragment name followed by the sequence and the list of chemical shifts are shown. Each chemical shift line includes the residue number, residue type, atom name and chemical shift (ppm).

Nup98^{FG1-15}

MFNKSFGTPF

GGGTG

1 M CA 55.148
1 M CB 33.364
1 M HA 3.97
2 F CA 57.943
2 F CB 39.642
2 F HA 4.637
2 F HN 8.284
2 F N 121.816
3 N CA 52.915
3 N CB 39.061
3 N HA 4.603
3 N HN 8.528
3 N N 122.099
4 K CA 56.784
4 K CB 42.058
4 K HA 4.192
4 K HN 8.419
4 K N 122.541
5 S CA 58.398
5 S CB 63.693
5 S HA 4.391
5 S HN 8.378
5 S N 116.589
6 F CA 58.058
6 F CB 39.648
6 F HA 4.615
6 F HN 8.327
6 F N 122.114
7 G CA 45.036
7 G HA2 3.893
7 G HA3 3.947
7 G HN 8.416
7 G N 110.356
8 T CA 59.827
8 T CB 69.759
8 T HA 4.613
8 T HN 8.183
8 T N 116.035
9 P CA 63.355
9 P CB 32.043
9 P HA 4.377
10 F CA 58.201
10 F CB 39.315
10 F HA 4.572
10 F HN 8.526
10 F N 120.757
11 G CA 45.37
11 G HA2 3.829
11 G HA3 3.899
11 G HN 8.441
11 G N 111.84
12 G CA 45.245
12 G HA2 3.942
12 G HA3 3.942
12 G HN 8.046
12 G N 108.172
13 G CA 45.166
13 G HA2 4.043
13 G HA3 4.043
13 G HN 8.451
13 G N 108.678
14 T CA 62.133
14 T CB 69.713
14 T HA 4.361
14 T HN 8.36
14 T N 112.923
15 G CA 44.972
15 G HA2 3.883
15 G HA3 3.926
15 G HN 8.639
15 G N 112.176

Nup98^{FG5-44}

SFGTPFGGGT

GGFGTTSTFG

QNTGFGTTSG

GAFGTSAFGS

1 S CA 58.221
1 S CB 63.751
1 S HA 4.37
1 S HN 8.314
1 S N 119.811
2 F CA 57.899
2 F CB 39.564
2 F HA 4.654
2 F HN 8.469
2 F N 122.366
3 G CA 45.101
3 G HA2 3.886
3 G HA3 3.94
3 G HN 8.464
3 G N 110.737
4 T CA 59.857
4 T CB 69.798
4 T HA 4.615
4 T HN 8.178
4 T N 116.315
5 P CA 63.381
5 P CB 32.036
5 P HA 4.374
6 F CA 58.333
6 F CB 39.34
6 F HA 4.573
6 F HN 8.497
6 F N 120.952
7 G CA 45.303
7 G HA2 3.823
7 G HA3 3.874
7 G HN 8.417
7 G N 112
8 G CA 45.286
8 G HA2 3.925
8 G HA3 3.925
8 G HN 8.01
8 G N 108.431
9 G CA 45.211
9 G HA2 4.035
9 G HA3 4.035
9 G HN 8.411
9 G N 108.93
10 T CA 62.039
10 T CB 69.795
10 T HA 4.376
10 T HN 8.328
10 T N 113.057
11 G CA 45.286
11 G HA2 3.933
11 G HA3 3.933
11 G HN 8.612
11 G N 111.485
12 G CA 45.026
12 G HA2 3.858
12 G HA3 3.858
12 G HN 8.263
12 G N 108.624
13 F CA 58.16
13 F CB 39.504
13 F HA 4.599
13 F HN 8.327
13 F N 120.275
14 G CA 45.336
14 G HA2 3.887
14 G HA3 3.975
14 G HN 8.534
14 G N 111.129
15 T CA 61.844
15 T CB 70.016

15 T HA 4.463
15 T HN 8.189
15 T N 113.534
16 T CA 61.887
16 T CB 69.848
16 T HA 4.414
16 T HN 8.355
16 T N 116.345
17 S CA 58.396
17 S CB 63.882
17 S HA 4.513
17 S HN 8.501
17 S N 118.596
18 T CA 61.956
18 T CB 69.632
18 T HA 4.306
18 T HN 8.278
18 T N 115.958
19 F CA 58.167
19 F CB 39.509
19 F HA 4.565
19 F HN 8.333
19 F N 122.83
20 G CA 45.278
20 G HA2 3.755
20 G HA3 3.887
20 G HN 8.426
20 G N 111.484
21 Q CA 55.852
21 Q CB 29.47
21 Q HA 4.303
21 Q HN 8.244
21 Q N 119.725
22 N CA 53.317
22 N CB 38.68
22 N HA 4.77
22 N HN 8.71
22 N N 120.15
23 T CA 62.095
23 T CB 69.728
23 T HA 4.301
23 T HN 8.255
23 T N 114.055
24 G CA 45.303
24 G HA2 3.825
24 G HA3 3.908
24 G HN 8.46
24 G N 110.789
25 F CA 58.039
25 F CB 39.529
25 F HA 4.602
25 F HN 8.266
25 F N 120.414
26 G CA 45.336
26 G HA2 3.887
26 G HA3 3.975
26 G HN 8.534
26 G N 111.129
27 T CA 61.844
27 T CB 70.016
27 T HA 4.463
27 T HN 8.189
27 T N 113.534
28 T CA 61.904
28 T CB 70.016
28 T HA 4.452
28 T HN 8.397
28 T N 116.026
29 S CA 58.614
29 S CB 63.842
29 S HA 4.478
29 S HN 8.492
29 S N 118.264
30 G CA 45.359
30 G HA2 3.977
30 G HA3 3.977
30 G HN 8.544
30 G N 111.145
31 G CA 45.085
31 G HA2 3.901
31 G HA3 3.901
31 G HN 8.293
31 G N 108.828
32 A CA 52.479
32 A CB 19.138
32 A HA 4.245
32 A HN 8.301
32 A N 123.879
33 F CA 58.005
33 F CB 39.514
33 F HA 4.554
33 F HN 8.411
33 F N 119.678
34 G CA 45.328
34 G HA2 3.875
34 G HA3 3.972
34 G HN 8.325
34 G N 111.017
35 T CA 61.792
35 T CB 70.016
35 T HA 4.414
35 T HN 8.144
35 T N 113.318
36 S CA 58.415
36 S CB 63.866
36 S HA 4.444
36 S HN 8.507
36 S N 118.482
37 A CA 52.6
37 A CB 19.129
37 A HA 4.255
37 A HN 8.438
37 A N 126.057
38 F CA 58.152
38 F CB 39.509
38 F HA 4.57
38 F HN 8.314
38 F N 121.85
39 G CA 45.286
39 G HA2 3.802
39 G HA3 3.96
39 G HN 8.358
39 G N 112.053
40 S CA 59.972
40 S CB 63.866
40 S HA 4.292
40 S HN 7.903
40 S N 121.388

Nup98$^{FG30-59}$
GTTSGGAFGT
SAFGSSNNTG
GLFGNSQTKP

1 G CA 45.361
1 G HA2 3.971
1 G HA3 3.971
1 G HN 8.439
1 G N 114.676
2 T CA 61.826
2 T CB 69.879
2 T HA 4.451
2 T HN 8.347
2 T N 113.591
3 T CA 61.818
3 T CB 69.879
3 T HA 4.428
3 T HN 8.397
3 T N 116.157
4 S CA 58.513
4 S CB 63.653
4 S HA 4.469
4 S HN 8.493
4 S N 118.368
5 G CA 45.415
5 G HA2 3.957
5 G HA3 3.957
5 G HN 8.574
5 G N 111.331
6 G CA 45.144
6 G HA2 3.895
6 G HA3 3.895
6 G HN 8.317
6 G N 108.824
7 A CA 52.543
7 A CB 19.072
7 A HA 4.235
7 A HN 8.314

7 A N 123.938
8 F CA 58.088
8 F CB 39.432
8 F HA 4.534
8 F HN 8.41
8 F N 119.71
9 G CA 45.376
9 G HA2 3.866
9 G HA3 3.96
9 G HN 8.32
9 G N 110.963
10 T CA 61.827
10 T CB 70.042
10 T HA 4.389
10 T HN 8.151
10 T N 113.352
11 S CA 58.462
11 S CB 63.815
11 S HA 4.424
11 S HN 8.506
11 S N 118.367
12 A CA 52.633
12 A CB 19.031
12 A HA 4.233
12 A HN 8.422
12 A N 126.071
13 F CA 58.12
13 F CB 39.432
13 F HA 4.545
13 F HN 8.279
13 F N 119.617
14 G CA 45.289
14 G HA2 3.829
14 G HA3 3.944
14 G HN 8.349
14 G N 111.204
15 S CA 58.449
15 S CB 63.936
15 S HA 4.473
15 S HN 8.277
15 S N 115.805
16 S CA 58.597
16 S CB 63.727
16 S HA 4.453
16 S HN 8.544
16 S N 117.844
17 N CA 53.312
17 N CB 38.711
17 N HA 4.701
17 N HN 8.511
17 N N 120.494
18 N CA 53.304
18 N CB 38.695
18 N HA 4.767
18 N HN 8.491
18 N N 119.483
19 T CA 62.232
19 T CB 69.645
19 T HA 4.333
19 T HN 8.291
19 T N 113.926
20 G CA 45.38
20 G HA2 3.975
20 G HA3 3.975
20 G HN 8.574
20 G N 111.331
21 G CA 45.199
21 G HA2 3.886
21 G HA3 3.886
21 G HN 8.318
21 G N 108.822
22 L CA 55.343
22 L CB 42.226
22 L HA 4.221
22 L HN 8.199
22 L N 121.736
23 F CA 57.733
23 F CB 39.394
23 F HA 4.615
23 F HN 8.409
23 F N 120.087
24 G CA 45.396
24 G HA2 3.856
24 G HA3 3.907
24 G HN 8.32
24 G N 110.278
25 N CA 53.22
25 N CB 39.005
25 N HA 4.759
25 N HN 8.422
25 N N 118.978
26 S CA 58.815
26 S CB 63.648
26 S HA 4.407
26 S HN 8.48
26 S N 116.438
27 Q CA 55.891
27 Q CB 29.445
27 Q HA 4.38
27 Q HN 8.55
27 Q N 122.068
28 T CA 62.164
28 T CB 69.769
28 T HA 4.257
28 T HN 8.273
28 T N 116.337
29 K CA 54.188
29 K CB 32.457
29 K HA 4.594
29 K HN 8.518
29 K N 125.998
30 P CA 63.01
30 P CB 32.341
30 P HA 4.353

Nup98$^{FG45-84}$

SNNTGGLFGN
SQTKPGGLFG
TSSFSQPATS
TSTGFGFGTS

1 S CA 58.555
1 S CB 63.637
1 S HA 4.408
1 S HN 8.489
1 S N 121.8
2 N CA 53.258
2 N CB 38.675
2 N HA 4.741
2 N HN 8.704
2 N N 120.52
3 N CA 53.294
3 N CB 38.624
3 N HA 4.795
3 N HN 8.54
3 N N 119.471
4 T CA 62.214
4 T CB 69.679
4 T HA 4.36
4 T HN 8.302
4 T N 113.928
5 G CA 45.177
5 G HA2 3.965
5 G HA3 3.994
5 G HN 8.59
5 G N 111.367
6 G CA 45.231
6 G HA2 3.911
6 G HA3 3.911
6 G HN 8.341
6 G N 108.759
7 L CA 55.37
7 L CB 42.264
7 L HA 4.24
7 L HN 8.215
7 L N 121.772
8 F CA 57.734
8 F CB 39.417
8 F HA 4.635
8 F HN 8.412
8 F N 120.061
9 G CA 45.376
9 G HA2 3.878
9 G HA3 3.942
9 G HN 8.326
9 G N 110.253
10 N CA 53.245
10 N CB 39.008

10 N HA 4.776
10 N HN 8.431
10 N N 118.985
11 S CA 58.555
11 S CB 63.8
11 S HA 4.413
11 S HN 8.486
11 S N 116.418
12 Q CA 55.97
12 Q CB 29.446
12 Q HA 4.392
12 Q HN 8.556
12 Q N 121.965
13 T CA 62.131
13 T CB 69.781
13 T HA 4.278
13 T HN 8.254
13 T N 115.915
14 K CA 54.241
14 K CB 32.533
14 K HA 4.61
14 K HN 8.471
14 K N 125.719
15 P CA 63.493
15 P CB 32.136
15 P HA 4.415
16 G CA 45.456
16 G HA2 3.959
16 G HA3 3.987
16 G HN 8.673
16 G N 110.149
17 G CA 45.393
17 G HA2 3.894
17 G HA3 3.939
17 G HN 8.344
17 G N 108.732
18 L CA 55.267
18 L CB 42.314
18 L HA 4.265
18 L HN 8.287
18 L N 121.693
19 F CA 57.939
19 F CB 39.492
19 F HA 4.567
19 F HN 8.443
19 F N 120.715
20 G CA 45.456
20 G HA2 3.889
20 G HA3 3.978
20 G HN 8.313
20 G N 110.858
21 T CA 61.841
21 T CB 70.061
21 T HA 4.414
21 T HN 8.178
21 T N 113.337
22 S CA 58.564
22 S CB 63.825
22 S HA 4.497
22 S HN 8.56
22 S N 118.242
23 S CA 58.877
23 S CB 63.699
23 S HA 4.414
23 S HN 8.413
23 S N 117.908
24 F CA 57.856
24 F CB 39.62
24 F HA 4.636
24 F HN 8.262
24 F N 122.216
25 S CA 58.008
25 S CB 63.863
25 S HA 4.394
25 S HN 8.211
25 S N 118.103
26 Q CA 53.667
26 Q CB 28.972
26 Q HA 4.568
26 Q HN 8.417
26 Q N 123.474
27 P CA 63.058
27 P CB 32.207
27 P HA 4.402
28 A CA 52.615
28 A CB 19.161
28 A HA 4.35
28 A HN 8.667
28 A N 125.037
29 T CA 61.675
29 T CB 70.034
29 T HA 4.372
29 T HN 8.294
29 T N 113.448
30 S CA 58.368
30 S CB 63.823
30 S HA 4.53
30 S HN 8.499
30 S N 118.151
31 T CA 61.913
31 T CB 69.786
31 T HA 4.41
31 T HN 8.362
31 T N 115.779
32 S CA 58.472
32 S CB 63.745
32 S HA 4.498
32 S HN 8.446
32 S N 118.424
33 T CA 62.001
33 T CB 69.795
33 T HA 4.333
33 T HN 8.273
33 T N 115.545
34 G CA 45.095
34 G HA2 3.842
34 G HA3 3.941
34 G HN 8.4
34 G N 110.907
35 F CA 58.168
35 F CB 39.569
35 F HA 4.562
35 F HN 8.303
35 F N 120.447
36 G CA 45.08
36 G HA2 3.707
36 G HA3 3.849
36 G HN 8.432
36 G N 111.046
37 F CA 58.1
37 F CB 39.572
37 F HA 4.608
37 F HN 8.242
37 F N 120.416
38 G CA 45.41
38 G HA2 3.883
38 G HA3 4
38 G HN 8.578
38 G N 111.313
39 T CA 61.85
39 T CB 69.866
39 T HA 4.415
39 T HN 8.237
39 T N 113.637
40 S CA 58.37
40 S CB 63.832
40 S HA 4.433
40 S HN 8.458
40 S N 118.793

Nup98$^{FG72\text{-}99}$
ATSTSTGFGF
GTSTGTANTL
FGTASTGT

1 A CA 52.665
1 A CB 19.214
1 A HA 4.328
1 A HN 8.466
1 A N 130.162
2 T CA 61.719
2 T CB 69.979
2 T HA 4.418
2 T HN 8.364
2 T N 113.292
3 S CA 58.406
3 S CB 63.735
3 S HA 4.519

3 S HN 8.5
3 S N 118.18
4 T CA 61.486
4 T CB 69.895
4 T HA 4.412
4 T HN 8.365
4 T N 115.728
5 S CA 58.439
5 S CB 63.699
5 S HA 4.5
5 S HN 8.446
5 S N 118.381
6 T CA 62.041
6 T CB 69.854
6 T HA 4.33
6 T HN 8.275
6 T N 115.545
7 G CA 45.052
7 G HA2 3.841
7 G HA3 3.938
7 G HN 8.396
7 G N 110.885
8 F CA 58.19
8 F CB 39.551
8 F HA 4.561
8 F HN 8.292
8 F N 120.415
9 G CA 45.057
9 G HA2 3.714
9 G HA3 3.852
9 G HN 8.435
9 G N 111.001
10 F CA 58.1
10 F CB 39.557
10 F HA 4.607
10 F HN 8.232
10 F N 120.417
11 G CA 45.365
11 G HA2 3.9
11 G HA3 3.996
11 G HN 8.565
11 G N 111.241
12 T CA 61.836
12 T CB 69.844
12 T HA 4.42
12 T HN 8.172
12 T N 113.524
13 S CA 58.415
13 S CB 63.687
13 S HA 4.56
13 S HN 8.593
13 S N 118.47
14 T CA 62.108
14 T CB 69.844
14 T HA 4.375
14 T HN 8.356
14 T N 115.665
15 G CA 45.391
15 G HA2 4.012
15 G HA3 4.012
15 G HN 8.486
15 G N 111.324
16 T CA 61.932
16 T CB 69.927
16 T HA 4.334
16 T HN 8.192
16 T N 114.013
17 A CA 52.815
17 A CB 19.172
17 A HA 4.301
17 A HN 8.511
17 A N 126.603
18 N CA 53.345
18 N CB 38.768
18 N HA 4.712
18 N HN 8.532
18 N N 118.253
19 T CA 62.097
19 T CB 69.807
19 T HA 4.27
19 T HN 8.177
19 T N 114.057
20 L CA 55.344
20 L CB 42.161
20 L HA 4.283
20 L HN 8.278
20 L N 124.2
21 F CA 57.956
21 F CB 39.558
21 F HA 4.617
21 F HN 8.3
21 F N 120.864
22 G CA 45.352
22 G HA2 3.899
22 G HA3 3.975
22 G HN 8.368
22 G N 110.846
23 T CA 61.898
23 T CB 69.95
23 T HA 4.336
23 T HN 8.174
23 T N 114.755
24 A CA 52.619
24 A CB 19.204
24 A HA 4.369
24 A HN 8.537
24 A N 126.78
25 S CA 58.439
25 S CB 63.773
25 S HA 4.5
25 S HN 8.495
25 S N 115.679
26 T CA 62.06
26 T CB 69.864
26 T HA 4.385
26 T HN 8.316
26 T N 115.631
27 G CA 45.406
27 G HA2 4.037
27 G HA3 4.037
27 G HN 8.528
27 G N 111.323
28 T CA 61.544
28 T CB 69.752
28 T HA 4.321
28 T HN 8.214
28 T N 113.699

Nup98$^{FG95-124}$
ASTGTSLFSS
QNNAFAQNKP
TGFGNFGTST

1 A CA 52.639
1 A CB 19.306
1 A HA 4.305
1 A HN 8.457
1 A N 130.118
2 S CA 58.276
2 S CB 63.701
2 S HA 4.547
2 S HN 8.615
2 S N 115.500
3 T CA 62.017
3 T CB 69.775
3 T HA 4.389
3 T HN 8.346
3 T N 115.646
4 G CA 45.447
4 G HA2 4.039
4 G HA3 4.039
4 G HN 8.555
4 G N 111.257
5 T CA 61.935
5 T CB 69.879
5 T HA 4.377
5 T HN 8.229
5 T N 113.941
6 S CA 58.450
6 S CB 63.749
6 S HA 4.452
6 S HN 8.523
6 S N 118.780
7 L CA 55.562
7 L CB 42.142
7 L HA 4.274
7 L HN 8.417
7 L N 124.461

8 F CA 57.974
8 F CB 39.669
8 F HA 4.621
8 F HN 8.303
8 F N 120.449
9 S CA 58.344
9 S CB 63.819
9 S HA 4.435
9 S HN 8.251
9 S N 117.345
10 S CA 58.883
10 S CB 63.631
10 S HA 4.417
10 S HN 8.494
10 S N 118.378
11 Q CA 56.203
11 Q CB 29.275
11 Q HA 4.306
11 Q HN 8.425
11 Q N 121.699
12 N CA 53.322
12 N CB 38.742
12 N HA 4.664
12 N HN 8.431
12 N N 119.555
13 N CA 53.372
13 N CB 38.774
13 N HA 4.654
13 N HN 8.440
13 N N 119.728
14 A CA 53.181
14 A CB 18.839
14 A HA 4.181
14 A HN 8.314
14 A N 124.125
15 F CA 57.731
15 F CB 39.218
15 F HA 4.572
15 F HN 8.167
15 F N 118.624
16 A CA 52.644
16 A CB 19.173
16 A HA 4.245
16 A HN 8.104
16 A N 124.857
17 Q CA 55.866
17 Q CB 29.354
17 Q HA 4.266
17 Q HN 8.351
17 Q N 119.131
18 N CA 52.965
18 N CB 38.794
18 N HA 4.669
18 N HN 8.492
18 N N 119.672
19 K CA 54.251
19 K CB 32.498
19 K HA 4.576
19 K HN 8.351
19 K N 122.840
20 P CA 63.124
20 P CB 32.164
20 P HA 4.487
21 T CA 62.171
21 T CB 69.908
21 T HA 4.281
21 T HN 8.448
21 T N 114.782
22 G CA 45.111
22 G HA2 3.854
22 G HA3 3.947
22 G HN 8.465
22 G N 111.133
23 F CA 58.048
23 F CB 39.505
23 F HA 4.607
23 F HN 8.303
23 F N 120.270
24 G CA 45.205
24 G HA2 3.729
24 G HA3 3.851
24 G HN 8.458
24 G N 111.023
25 N CA 53.033
25 N CB 38.720
25 N HA 4.675
25 N HN 8.267
25 N N 118.797
26 F CA 58.033
26 F CB 39.374
26 F HA 4.635
26 F HN 8.432
26 F N 120.971
27 G CA 45.381
27 G HA2 3.937
27 G HA3 4.000
27 G HN 8.487
27 G N 110.648
28 T CA 61.752
28 T CB 69.947
28 T HA 4.421
28 T HN 8.187
28 T N 113.586
29 S CA 58.468
29 S CB 63.684
29 S HA 4.559
29 S HN 8.599
29 S N 118.602
30 T CA 61.650
30 T CB 69.645
30 T HA 4.332
30 T HN 8.318
30 T N 116.226

Nup98$^{FG115\text{-}144}$
TGFGNFGTST
SSGGLFGTTN
TTSNPFGSTS

1 T CA 62.117
1 T CB 69.826
1 T HA 4.272
1 T HN 8.349
1 T N 119.179
2 G CA 45.156
2 G HA2 3.836
2 G HA3 3.909
2 G HN 8.529
2 G N 111.069
3 F CA 58.009
3 F CB 39.598
3 F HA 4.628
3 F HN 8.275
3 F N 120.143
4 G CA 45.23
4 G HA2 3.746
4 G HA3 3.852
4 G HN 8.45
4 G N 110.668
5 N CA 53.004
5 N CB 38.732
5 N HA 4.663
5 N HN 8.263
5 N N 118.733
6 F CA 57.989
6 F CB 39.37
6 F HA 4.628
6 F HN 8.407
6 F N 120.883
7 G CA 45.368
7 G HA2 3.928
7 G HA3 3.99
7 G HN 8.46
7 G N 110.566
8 T CA 61.768
8 T CB 69.981
8 T HA 4.424
8 T HN 8.179
8 T N 113.405
9 S CA 58.577
9 S CB 63.873
9 S HA 4.564
9 S HN 8.563
9 S N 118.356
10 T CA 61.918
10 T CB 69.75
10 T HA 4.422
10 T HN 8.385

10 T N 115.744
11 S CA 58.497
11 S CB 63.827
11 S HA 4.506
11 S HN 8.439
11 S N 118.168
12 S CA 58.668
12 S CB 63.822
12 S HA 4.481
12 S HN 8.505
12 S N 118.115
13 G CA 45.407
13 G HA2 3.98
13 G HA3 3.98
13 G HN 8.515
13 G N 110.987
14 G CA 45.175
14 G HA2 3.906
14 G HA3 3.906
14 G HN 8.299
14 G N 108.726
15 L CA 55.275
15 L CB 42.292
15 L HA 4.256
15 L HN 8.213
15 L N 121.712
16 F CA 57.921
16 F CB 39.462
16 F HA 4.626
16 F HN 8.432
16 F N 120.593
17 G CA 45.368
17 G HA2 3.929
17 G HA3 3.976
17 G HN 8.336
17 G N 110.636
18 T CA 61.807
18 T CB 70.018
18 T HA 4.457
18 T HN 8.185
18 T N 113.41
19 T CA 61.952
19 T CB 69.866
19 T HA 4.39
19 T HN 8.352
19 T N 116.282
20 N CA 53.346
20 N CB 38.873
20 N HA 4.806
20 N HN 8.644
20 N N 121.778
21 T CA 61.869
21 T CB 69.738
21 T HA 4.426
21 T HN 8.349
21 T N 114.773
22 T CA 62.082
22 T CB 69.816
22 T HA 4.379
22 T HN 8.312
22 T N 116.228
23 S CA 58.428
23 S CB 63.739
23 S HA 4.428
23 S HN 8.409
23 S N 118.239
24 N CB 38.884
24 N HN 8.528
24 N N 121.452
25 P CA 63.682
25 P CB 31.901
25 P HA 4.334
26 F CA 57.87
26 F CB 39.02
26 F HA 4.634
26 F HN 8.334
26 F N 119.545
27 G CA 45.343
27 G HA2 3.889
27 G HA3 3.971
27 G HN 8.162
27 G N 110.507
28 S CA 58.419
28 S CB 63.893
28 S HA 4.549
28 S HN 8.34
28 S N 115.756
29 T CA 61.918
29 T CB 69.719
29 T HA 4.45
29 T HN 8.47
29 T N 115.966
30 S CA 58.328
30 S CB 63.858
30 S HA 4.427
30 S HN 8.373
30 S N 118.749

Nup98$^{FG137\text{-}170}$
SNPFGSTSGS
LFGPSSFTAA
PTGTTIKFNP
PTGT

1 S CA 58.42
1 S CB 63.833
1 S HA 4.387
1 S HN 8.438
1 S N 121.421
2 N CB 38.87
2 N HN 8.681
2 N N 120.971
3 P CA 63.678
3 P CB 31.895
3 P HA 4.331
4 F CA 57.86
4 F CB 39.032
4 F HA 4.634
4 F HN 8.34
4 F N 119.158
5 G CA 45.383
5 G HA2 3.898
5 G HA3 3.969
5 G HN 8.16
5 G N 110.079
6 S CA 58.408
6 S CB 63.958
6 S HA 4.547
6 S HN 8.343
6 S N 115.401
7 T CA 61.837
7 T CB 69.742
7 T HA 4.453
7 T HN 8.459
7 T N 117.826
8 S CA 58.808
8 S CB 63.738
8 S HA 4.448
8 S HN 8.46
8 S N 115.486
9 G CA 45.35
9 G HA2 3.982
9 G HA3 3.982
9 G HN 8.548
9 G N 110.973
10 S CA 58.368
10 S CB 63.842
10 S HA 4.415
10 S HN 8.262
10 S N 115.374
11 L CA 55.446
11 L CB 42.166
11 L HA 4.262
11 L HN 8.37
11 L N 123.868
12 F CA 57.377
12 F CB 39.886
12 F HA 4.697
12 F HN 8.237
12 F N 119.508
13 G CA 44.646
13 G HA2 4.059
13 G HA3 4.093
13 G HN 8.228
13 G N 110.081
14 P CA 63.501
14 P CB 32.168
14 P HA 4.427

15 S CA 58.515
15 S CB 63.765
15 S HA 4.467
15 S HN 8.613
15 S N 115.643
16 S CA 58.568
16 S CB 63.825
16 S HA 4.403
16 S HN 8.341
16 S N 117.49
17 F CA 57.851
17 F CB 39.742
17 F HA 4.685
17 F HN 8.284
17 F N 121.853
18 T CA 61.627
18 T CB 70.004
18 T HA 4.242
18 T HN 8.124
18 T N 117.146
19 A CA 52.14
19 A CB 19.317
19 A HA 4.25
19 A HN 8.372
19 A N 127.251
20 A CA 50.481
20 A CB 17.929
20 A HA 4.546
20 A HN 8.44
20 A N 125.13
21 P CA 63.064
21 P CB 32.108
21 P HA 4.497
22 T CA 62.047
22 T CB 69.962
22 T HA 4.341
22 T HN 8.456
22 T N 114.504
23 G CA 45.337
23 G HA2 4.037
23 G HA3 4.037
23 G HN 8.592
23 G N 111.172
24 T CA 61.932
24 T CB 69.786
24 T HA 4.423
24 T HN 8.239
24 T N 113.872
25 T CA 62.138
25 T CB 69.848
25 T HA 4.348
25 T HN 8.389
25 T N 117.666
26 I CA 61.104
26 I CB 38.562
26 I HA 4.1
26 I HN 8.365
26 I N 124.575
27 K CA 55.966
27 K CB 33.244
27 K HA 4.285
27 K HN 8.482
27 K N 126.4
28 F CA 57.419
28 F CB 39.921
28 F HA 4.615
28 F HN 8.461
28 F N 122.747
29 N CB 39.034
29 N HN 8.53
29 N N 123.627
30 P CA 61.488
30 P CB 30.894
30 P HA 4.544
31 P CA 63.047
31 P CB 32.093
31 P HA 4.5
32 T CA 62.102
32 T CB 69.908
32 T HA 4.317
32 T HN 8.422
32 T N 114.473
33 G CA 45.339
33 G HA2 4.049
33 G HA3 4.049
33 G HN 8.606
33 G N 111.324
34 T CA 61.635
34 T CB 69.899
34 T HA 4.332
34 T HN 8.235
34 T N 113.388

Nup98$^{FG165-204}$

NPPTGTDTMV
KAGVSTNIST
KHQCITAMKE
YESKSLEELR

1 N CA 51.494
1 N CB 38.354
1 N HA 4.97
1 N HN 8.572
1 N N 125.768
2 P CA 63.19
2 P CB 32.061
2 P HA 4.479
3 P CA 63.013
3 P CB 32.105
3 P HA 4.534
4 T CA 62.101
4 T CB 70.027
4 T HA 4.358
4 T HN 8.48
4 T N 114.75
5 G CA 45.398
5 G HA2 4.062
5 G HA3 4.062
5 G HN 8.614
5 G N 111.492
6 T CA 61.885
6 T CB 69.651
6 T HA 4.361
6 T HN 8.277
6 T N 113.71
7 D CA 54.586
7 D CB 40.986
7 D HA 4.678
7 D HN 8.6
7 D N 122.964
8 T CA 62.305
8 T CB 69.542
8 T HA 4.284
8 T HN 8.218
8 T N 114.606
9 M CA 55.765
9 M CB 32.646
9 M HA 4.475
9 M HN 8.43
9 M N 122.962
10 V CA 62.669
10 V CB 32.635
10 V HA 4.042
10 V HN 8.262
10 V N 120.494
11 K CA 56.116
11 K CB 33.119
11 K HA 4.312
11 K HN 8.557
11 K N 126.5
12 A CA 52.666
12 A CB 19.208
12 A HA 4.292
12 A HN 8.526
12 A N 126.354
13 G CA 45.181
13 G HA2 3.953
13 G HA3 4.001
13 G HN 8.538
13 G N 108.833
14 V CA 62.243
14 V CB 32.92
14 V HA 4.191
14 V HN 8.127
14 V N 119.671
15 S CA 58.335
15 S CB 63.62
15 S HA 4.563

15 S HN 8.676
15 S N 120.24
16 T CA 61.963
16 T CB 69.733
16 T HA 4.36
16 T HN 8.384
16 T N 116.293
17 N CA 53.364
17 N CB 38.76
17 N HA 4.743
17 N HN 8.559
17 N N 121.577
18 I CA 61.369
18 I CB 38.678
18 I HA 4.211
18 I HN 8.29
18 I N 121.675
19 S CA 58.49
19 S CB 63.753
19 S HA 4.529
19 S HN 8.593
19 S N 120.226
20 T CA 62.098
20 T CB 69.684
20 T HA 4.322
20 T HN 8.351
20 T N 116.733
21 K CA 55.921
21 K CB 32.899
21 K HA 4.264
21 K HN 8.336
21 K N 123.575
22 H CA 55.612
22 H CB 29.378
22 H HA 4.661
22 H HN 8.625
22 H N 120.294
23 Q CA 55.929
23 Q CB 29.649
23 Q HA 4.334
23 Q HN 8.594
23 Q N 122.562
24 C CA 58.641
24 C CB 27.892
24 C HA 4.52
24 C HN 8.684
24 C N 122.012
25 I CA 61.67
25 I CB 38.633
25 I HA 4.244
25 I HN 8.574
25 I N 122.123
26 T CA 62.248
26 T CB 69.684
26 T HA 4.271
26 T HN 8.321
26 T N 119.07
27 A CA 52.691
27 A CB 19.147
27 A HA 4.321
27 A HN 8.418
27 A N 126.824
28 M CA 55.776
28 M CB 32.824
28 M HA 4.425
28 M HN 8.406
28 M N 120.194
29 K CA 56.585
29 K CB 32.939
29 K HA 4.25
29 K HN 8.47
29 K N 123.149
30 E CA 57.065
30 E CB 30.106
30 E HA 4.2
30 E HN 8.574
30 E N 124.499
31 Y CA 58.38
31 Y CB 38.737
31 Y HA 4.506
31 Y HN 8.263
31 Y N 122.832
32 E CA 56.646
32 E CB 30.492
32 E HA 4.248
32 E HN 8.302
32 E N 122.819
33 S CA 58.823
33 S CB 63.761
33 S HA 4.382
33 S HN 8.431
33 S N 117.409
34 K CA 56.634
34 K CB 33.175
34 K HA 4.364
34 K HN 8.456
34 K N 123.725
35 S CA 58.605
35 S CB 63.882
35 S HA 4.397
35 S HN 8.536
35 S N 117.303
36 L CA 56.47
36 L CB 41.868
36 L HA 4.245
36 L HN 8.455
36 L N 123.524
37 E CA 57.964
37 E CB 29.794
37 E HA 4.109
37 E HN 8.393
37 E N 120.151
38 E CA 57.448
38 E CB 30.036
38 E HA 4.168
38 E HN 8.277
38 E N 121.241
39 L CA 55.884
39 L CB 42.081
39 L HA 4.27
39 L HN 8.228
39 L N 122.156
40 R CA 56.541
40 R CB 30.795
40 R HA 4.245
40 R HN 8.171
40 R N 121.262

Nup98$^{FG189-218}$

ITAMKEYESK
SLEELRLEDY
QANRKGPQNQ

1 I CA 61.675
1 I CB 38.753
1 I HA 4.174
1 I HN 8.368
1 I N 126.243
2 T CA 62.022
2 T CB 69.828
2 T HA 4.311
2 T HN 8.356
2 T N 118.699
3 A CA 52.615
3 A CB 19.182
3 A HA 4.315
3 A HN 8.435
3 A N 126.768
4 M CA 55.674
4 M CB 32.876
4 M HA 4.425
4 M HN 8.441
4 M N 120.134
5 K CA 56.545
5 K CB 32.966
5 K HA 4.253
5 K HN 8.481
5 K N 123.148
6 E CA 56.861
6 E CB 30.298
6 E HA 4.191
6 E HN 8.598
6 E N 122.013
7 Y CA 58.289
7 Y CB 38.774
7 Y HA 4.504
7 Y HN 8.269
7 Y N 120.244

8 E CA 56.545
8 E CB 30.537
8 E HA 4.253
8 E HN 8.307
8 E N 122.585
9 S CA 58.661
9 S CB 63.716
9 S HA 4.39
9 S HN 8.422
9 S N 117.207
10 K CA 56.645
10 K CB 33.609
10 K HA 4.388
10 K HN 8.413
10 K N 123.581
11 S CA 58.244
11 S CB 64.52
11 S HA 4.411
11 S HN 8.7
11 S N 117.719
12 L CA 56.992
12 L CB 41.796
12 L HA 4.193
12 L HN 8.555
12 L N 122.592
13 E CA 58.259
13 E CB 29.806
13 E HA 4.14
13 E HN 8.452
13 E N 119.631
14 E CA 58.13
14 E CB 30.099
14 E HA 4.105
14 E HN 8.169
14 E N 120.808
15 L CA 56.658
15 L CB 41.991
15 L HA 4.172
15 L HN 8.276
15 L N 121.726
16 R CA 56.545
16 R CB 30.434
16 R HA 4.253
16 R HN 8.316
16 R N 121.188
17 L CA 56.64
17 L CB 42.005
17 L HA 4.246
17 L HN 8.185
17 L N 122.032
18 E CA 58.14
18 E CB 30.462
18 E HA 4.146
18 E HN 8.364
18 E N 119.793
19 D CA 55.562
19 D CB 40.969
19 D HA 4.534
19 D HN 8.201
19 D N 120.394
20 Y CA 59.677
20 Y CB 38.32
20 Y HA 4.388
20 Y HN 8.181
20 Y N 120.768
21 Q CA 56.664
21 Q CB 28.997
21 Q HA 4.097
21 Q HN 8.257
21 Q N 119.957
22 A CA 53.565
22 A CB 18.833
22 A HA 4.161
22 A HN 8.112
22 A N 122.814
23 N CA 53.48
23 N CB 38.713
23 N HA 4.648
23 N HN 8.208
23 N N 115.603
24 R CA 56.664
24 R CB 30.516
24 R HA 4.262
24 R HN 8.061
24 R N 120.543
25 K CA 56.48
25 K CB 33.24
25 K HA 4.378
25 K HN 8.374
25 K N 121.821
26 G CA 44.62
26 G HA2 4.084
26 G HA3 4.124
26 G HN 8.338
26 G N 109.731
27 P CA 63.439
27 P CB 32.151
27 P HA 4.418
28 Q CA 55.761
28 Q CB 29.361
28 Q HA 4.32
28 Q HN 8.784
28 Q N 120.279
29 N CA 53.39
29 N CB 38.693
29 N HA 4.681
29 N HN 8.572
29 N N 119.479
30 Q CA 55.78
30 Q CB 29.475
30 Q HA 4.283
30 Q HN 8.543
30 Q N 121.165

Nup98$^{FG205-244}$

LEDYQANRKG
PQNQVGAGTT
TGLFGSSPAT
SSATGLFSSS

1 L CA 55.59
1 L CB 42.487
1 L HA 4.272
1 L HN 8.4
1 L N 127.946
2 E CA 56.754
2 E CB 30.089
2 E HA 4.214
2 E HN 8.614
2 E N 121.221
3 D CA 54.346
3 D CB 40.937
3 D HA 4.547
3 D HN 8.391
3 D N 121.739
4 Y CA 58.914
4 Y CB 38.261
4 Y HA 4.442
4 Y HN 8.276
4 Y N 121.696
5 Q CA 56.11
5 Q CB 29.164
5 Q HA 4.151
5 Q HN 8.246
5 Q N 121.406
6 A CA 53.349
6 A CB 18.919
6 A HA 4.144
6 A HN 8.149
6 A N 123.957
7 N CA 53.365
7 N CB 38.52
7 N HA 4.643
7 N HN 8.343
7 N N 116.32
8 R CA 56.363
8 R CB 30.607
8 R HA 4.287
8 R HN 8.153
8 R N 121.223
9 K CA 56.187
9 K CB 33.253
9 K HA 4.375
9 K HN 8.428
9 K N 122.446
10 G CA 44.607
10 G HA2 4.071
10 G HA3 4.122

10 G HN 8.361
10 G N 110.195
11 P CA 63.351
11 P CB 32.134
11 P HA 4.418
12 Q CA 55.783
12 Q CB 29.488
12 Q HA 4.312
12 Q HN 8.773
12 Q N 120.764
13 N CA 53.384
13 N CB 38.819
13 N HA 4.669
13 N HN 8.541
13 N N 119.658
14 Q CA 55.808
14 Q CB 29.514
14 Q HA 4.361
14 Q HN 8.478
14 Q N 120.94
15 V CA 62.79
15 V CB 32.645
15 V HA 4.088
15 V HN 8.403
15 V N 122.421
16 G CA 45.215
16 G HA2 3.955
16 G HA3 3.955
16 G HN 8.679
16 G N 113.414
17 A CA 52.749
17 A CB 19.351
17 A HA 4.327
17 A HN 8.387
17 A N 124.283
18 G CA 45.344
18 G HA2 4.002
18 G HA3 4.002
18 G HN 8.612
18 G N 108.576
19 T CA 61.885
19 T CB 69.919
19 T HA 4.461
19 T HN 8.202
19 T N 113.741
20 T CA 61.903
20 T CB 69.799
20 T HA 4.485
20 T HN 8.452
20 T N 116.472
21 T CA 62.108
21 T CB 69.868
21 T HA 4.361
21 T HN 8.287
21 T N 116.318
22 G CA 45.307
22 G HA2 3.927
22 G HA3 3.927
22 G HN 8.486
22 G N 111.344
23 L CA 55.21
23 L CB 42.356
23 L HA 4.272
23 L HN 8.177
23 L N 121.662
24 F CA 57.996
24 F CB 39.37
24 F HA 4.611
24 F HN 8.506
24 F N 121.104
25 G CA 45.267
25 G HA2 3.878
25 G HA3 3.955
25 G HN 8.394
25 G N 111.067
26 S CA 58.276
26 S CB 64.066
26 S HA 4.503
26 S HN 8.264
26 S N 115.698
27 S CA 56.597
27 S CB 63.326
27 S HA 4.804
27 S HN 8.516
27 S N 119.092
28 P CA 63.351
28 P CB 32.134
28 P HA 4.422
29 A CA 52.744
29 A CB 19.148
29 A HA 4.341
29 A HN 8.556
29 A N 124.408
30 T CA 61.743
30 T CB 69.913
30 T HA 4.373
30 T HN 8.235
30 T N 113.246
31 S CA 58.298
31 S CB 63.852
31 S HA 4.525
31 S HN 8.423
31 S N 118.123
32 S CA 58.463
32 S CB 63.883
32 S HA 4.475
32 S HN 8.536
32 S N 118.304
33 A CA 52.915
33 A CB 19.148
33 A HA 4.402
33 A HN 8.514
33 A N 126.448
34 T CA 62.038
34 T CB 69.919
34 T HA 4.336
34 T HN 8.193
34 T N 112.482
35 G HN 8.398
35 G N 111.081
36 L CA 55.204
36 L CB 42.362
36 L HA 4.276
36 L HN 8.131
36 L N 121.686
37 F CA 57.682
37 F CB 39.535
37 F HA 4.66
37 F HN 8.429
37 F N 121.002
38 S CA 58.065
38 S CB 63.935
38 S HA 4.457
38 S HN 8.268
38 S N 117.626
39 S CA 58.581
39 S CB 63.745
39 S HA 4.484
39 S HN 8.583
39 S N 118.709
40 S CA 58.401
40 S CB 63.852
40 S HA 4.438
40 S HN 8.406
40 S N 118.697

Nup98$^{FG225\text{-}264}$
TGLFGSSPAT
SSATGLFSSS
TTNSGFAYGQ
NKTAFGTSTT

1 T CA 62.26
1 T CB 69.825
1 T HA 4.292
1 T HN 8.392
1 T N 119.482
2 G CA 45.362
2 G HA2 3.913
2 G HA3 3.913
2 G HN 8.634
2 G N 111.534
3 L CA 55.172
3 L CB 42.251
3 L HA 4.272
3 L HN 8.137
3 L N 121.574
4 F CA 58.024

4 F CB 39.239
4 F HA 4.594
4 F HN 8.5
4 F N 120.992
5 G CA 45.29
5 G HA2 3.873
5 G HA3 3.955
5 G HN 8.39
5 G N 111.073
6 S CA 58.272
6 S CB 64.056
6 S HA 4.497
6 S HN 8.247
6 S N 115.685
7 S CA 56.597
7 S CB 63.324
7 S HA 4.801
7 S HN 8.512
7 S N 119.068
8 P CA 63.372
8 P CB 32.122
8 P HA 4.416
9 A CA 52.744
9 A CB 19.134
9 A HA 4.331
9 A HN 8.554
9 A N 124.374
10 T CA 61.73
10 T CB 69.907
10 T HA 4.37
10 T HN 8.229
10 T N 113.213
11 S CA 58.312
11 S CB 63.938
11 S HA 4.518
11 S HN 8.415
11 S N 118.103
12 S CA 58.477
12 S CB 63.815
12 S HA 4.472
12 S HN 8.53
12 S N 118.35
13 A CA 52.919
13 A CB 19.156
13 A HA 4.399
13 A HN 8.507
13 A N 126.431
14 T CA 62.054
14 T CB 69.894
14 T HA 4.331
14 T HN 8.188
14 T N 112.501
15 G CA 45.335
15 G HA2 3.925
15 G HA3 3.925
15 G HN 8.39
15 G N 111.073
16 L CA 55.193
16 L CB 42.348
16 L HA 4.273
16 L HN 8.125
16 L N 121.643
17 F CA 57.668
17 F CB 39.592
17 F HA 4.66
17 F HN 8.415
17 F N 120.972
18 S CA 58.118
18 S CB 63.964
18 S HA 4.455
18 S HN 8.275
18 S N 117.617
19 S CA 58.513
19 S CB 63.811
19 S HA 4.493
19 S HN 8.53
19 S N 118.35
20 S CA 58.64
20 S CB 63.802
20 S HA 4.543
20 S HN 8.495
20 S N 117.919
21 T CA 62.09
21 T CB 69.688
21 T HA 4.437
21 T HN 8.328
21 T N 115.812
22 T CA 62.154
22 T CB 69.783
22 T HA 4.343
22 T HN 8.229
22 T N 116.229
23 N CA 53.323
23 N CB 38.8
23 N HA 4.74
23 N HN 8.571
23 N N 121.524
24 S CA 58.893
24 S CB 63.732
24 S HA 4.378
24 S HN 8.441
24 S N 116.645
25 G CA 45.279
25 G HA2 3.853
25 G HA3 3.916
25 G HN 8.48
25 G N 110.585
26 F CA 57.875
26 F CB 39.729
26 F HA 4.541
26 F HN 8.115
26 F N 120.352
27 A CA 52.263
27 A CB 19.159
27 A HA 4.255
27 A HN 8.295
27 A N 125.682
28 Y CA 58.556
28 Y CB 38.605
28 Y HA 4.442
28 Y HN 8.161
28 Y N 119.881
29 G CA 45.385
29 G HA2 3.795
29 G HA3 3.916
29 G HN 8.413
29 G N 110.958
30 Q CA 55.907
30 Q CB 29.41
30 Q HA 4.285
30 Q HN 8.237
30 Q N 119.688
31 N CA 53.36
31 N CB 38.688
31 N HA 4.638
31 N HN 8.597
31 N N 119.68
32 K CA 56.565
32 K CB 32.997
32 K HA 4.323
32 K HN 8.427
32 K N 122.052
33 T CA 61.897
33 T CB 69.991
33 T HA 4.276
33 T HN 8.249
33 T N 115.227
34 A CA 52.481
34 A CB 19.162
34 A HA 4.256
34 A HN 8.378
34 A N 126.335
35 F CA 58.053
35 F CB 39.596
35 F HA 4.593
35 F HN 8.321
35 F N 119.96
36 G CA 45.299
36 G HA2 3.895
36 G HA3 3.969
36 G HN 8.39
36 G N 111.073
37 T CA 61.81
37 T CB 69.748
37 T HA 4.416
37 T HN 8.161
37 T N 113.706
38 S CA 58.361

38 S CB 63.815
38 S HA 4.581
38 S HN 8.584
38 S N 118.524
39 T CA 62.007
39 T CB 69.625
39 T HA 4.451
39 T HN 8.484
39 T N 116.418
40 T CA 61.696
40 T CB 70.002
40 T HA 4.331
40 T HN 8.2
40 T N 116.238

Nup98$^{FG256-275}$
KTAFGTSTTG
FGTNPGGLFG

1 K CA 56.637
1 K CB 33.214
1 K HA 4.326
1 K HN 8.476
1 K N 126.971
2 T CA 61.548
2 T CB 70.089
2 T HA 4.317
2 T HN 8.336
2 T N 115.335
3 A CA 52.417
3 A CB 19.212
3 A HA 4.284
3 A HN 8.445
3 A N 126.272
4 F CA 58.057
4 F CB 39.628
4 F HA 4.603
4 F HN 8.37
4 F N 119.791
5 G CA 45.303
5 G HA2 3.876
5 G HA3 3.977
5 G HN 8.412
5 G N 110.841
6 T CA 61.808
6 T CB 70.038
6 T HA 4.403
6 T HN 8.161
6 T N 113.311
7 S CA 58.42
7 S CB 63.81
7 S HA 4.565
7 S HN 8.582
7 S N 118.071
8 T CA 62.014
8 T CB 69.598
8 T HA 4.434
8 T HN 8.467
8 T N 115.878
9 T CA 62.092
9 T CB 69.835
9 T HA 4.339
9 T HN 8.215
9 T N 115.414
10 G CA 45.162
10 G HA2 3.835
10 G HA3 3.926
10 G HN 8.429
10 G N 110.844
11 F CA 58.104
11 F CB 39.616
11 F HA 4.618
11 F HN 8.321
11 F N 120.087
12 G CA 45.304
12 G HA2 3.875
12 G HA3 3.976
12 G HN 8.543
12 G N 110.816
13 T CA 61.827
13 T CB 69.876
13 T HA 4.319
13 T HN 8.144
13 T N 112.978
14 N CB 38.743
14 N HN 8.694
14 N N 121.566
15 P CA 63.96
15 P CB 31.923
15 P HA 4.402
16 G CA 45.251
16 G HA2 3.938
16 G HA3 3.938
16 G HN 8.614
16 G N 108.995
17 G CA 45.251
17 G HA2 3.912
17 G HA3 3.912
17 G HN 8.221
17 G N 108.298
18 L CA 55.296
18 L CB 42.295
18 L HA 4.252
18 L HN 8.203
18 L N 121.339
19 F CA 58.009
19 F CB 39.193
19 F HA 4.592
19 F HN 8.531
19 F N 120.836
20 G CA 44.954
20 G HA2 3.761
20 G HA3 3.857
20 G HN 8.371
20 G N 112.107

Nup98$^{FG265-304}$
GFGTNPGGLF
GQQNQQTTSL
FSKPFGQATT
TQNTGFSFGN

1 G CA 45.109
1 G HA2 3.809
1 G HA3 3.851
1 G HN 8.321
1 G N 114.231
2 F CA 58.075
2 F CB 39.467
2 F HA 4.614
2 F HN 8.38
2 F N 120.048
3 G CA 45.301
3 G HA2 3.869
3 G HA3 3.987
3 G HN 8.595
3 G N 111.077
4 T CA 61.822
4 T CB 69.783
4 T HA 4.324
4 T HN 8.132
4 T N 113.182
5 N CB 38.75
5 N HA 4.982
5 N HN 8.685
5 N N 121.805
6 P CA 63.947
6 P CB 31.93
6 P HA 4.401
7 G CA 45.273
7 G HA2 3.94
7 G HA3 3.94
7 G HN 8.608
7 G N 109.209
8 G CA 45.332
8 G HA2 3.908
8 G HA3 3.908
8 G HN 8.216
8 G N 108.621
9 L CA 55.342
9 L CB 42.239

9 L HA 4.255
9 L HN 8.2
9 L N 121.602
10 F CA 58.065
10 F CB 39.455
10 F HA 4.598
10 F HN 8.434
10 F N 120.515
11 G CA 45.338
11 G HA2 3.837
11 G HA3 3.919
11 G HN 8.361
11 G N 110.542
12 Q CA 56.088
12 Q CB 29.341
12 Q HA 4.305
12 Q HN 8.317
12 Q N 119.905
13 Q CA 56.088
13 Q CB 29.285
13 Q HA 4.303
13 Q HN 8.642
13 Q N 121.414
14 N CA 53.469
14 N CB 38.704
14 N HA 4.672
14 N HN 8.591
14 N N 119.7
15 Q CA 56.088
15 Q CB 29.341
15 Q HA 4.304
15 Q HN 8.511
15 Q N 120.79
16 Q CA 56.086
16 Q CB 29.366
16 Q HA 4.38
16 Q HN 8.594
16 Q N 121.529
17 T CA 62.055
17 T CB 69.766
17 T HA 4.403
17 T HN 8.394
17 T N 115.84
18 T CA 62.031
18 T CB 69.766
18 T HA 4.364
18 T HN 8.286
18 T N 116.664
19 S CA 58.405
19 S CB 63.755
19 S HA 4.436
19 S HN 8.478
19 S N 118.608
20 L CA 55.342
20 L CB 42.172
20 L HA 4.254
20 L HN 8.356
20 L N 122.579
21 F CA 57.535
21 F CB 39.541
21 F HA 4.652
21 F HN 8.233
21 F N 120.14
22 S CA 58.105
22 S CB 63.869
22 S HA 4.401
22 S HN 8.167
22 S N 117.626
23 K CA 54.384
23 K CB 32.579
23 K HA 4.56
23 K HN 8.368
23 K N 119.714
24 P CA 63.169
24 P CB 32.052
24 P HA 4.382
25 F CA 58.361
25 F CB 39.528
25 F HA 4.553
25 F HN 8.356
25 F N 122.579
26 G CA 45.228
26 G HA2 3.792
26 G HA3 3.866
26 G HN 8.398
26 G N 110.266
27 Q CA 55.664
27 Q CB 29.716
27 Q HA 4.316
27 Q HN 8.233
27 Q N 120.14
28 A CA 52.637
28 A CB 19.235
28 A HA 4.378
28 A HN 8.58
28 A N 126.059
29 T CA 61.83
29 T CB 69.766
29 T HA 4.414
29 T HN 8.383
29 T N 114.079
30 T CA 61.712
30 T CB 69.89
30 T HA 4.462
30 T HN 8.337
30 T N 116.343
31 T CA 62.022
31 T CB 69.783
31 T HA 4.329
31 T HN 8.324
31 T N 116.718
32 Q CA 55.877
32 Q CB 29.499
32 Q HA 4.333
32 Q HN 8.549
32 Q N 123.004
33 N CA 53.232
33 N CB 38.745
33 N HA 4.754
33 N HN 8.663
33 N N 120.671
34 T CA 62.073
34 T CB 69.766
34 T HA 4.312
34 T HN 8.296
34 T N 114.386
35 G CA 45.215
35 G HA2 3.86
35 G HA3 3.933
35 G HN 8.507
35 G N 110.971
36 F CA 57.877
36 F CB 39.78
36 F HA 4.585
36 F HN 8.2
36 F N 120.426
37 S CA 57.918
37 S CB 63.792
37 S HA 4.382
37 S HN 8.26
37 S N 117.91
38 F CA 58.184
38 F CB 39.505
38 F HA 4.557
38 F HN 8.52
38 F N 121.15
39 G CA 45.366
39 G HA2 3.817
39 G HA3 3.936
39 G HN 8.438
39 G N 110.598
40 N CA 52.935
40 N CB 39.042
40 N HA 4.697
40 N HN 8.366
40 N N 124.422

Nup98$^{FG298\text{-}327}$

TGFSFGNTST
IGQPSTNTMG
LFGVTQASQP

1 T CA 62.157
1 T CB 69.826
1 T HA 4.28
1 T HN 8.363
1 T N 122.542
2 G CA 45.225

2 G HA2 3.845
2 G HA3 3.921
2 G HN 8.577
2 G N 111.174
3 F CA 57.794
3 F CB 39.799
3 F HA 4.603
3 F HN 8.236
3 F N 120.444
4 S CA 58.068
4 S CB 63.815
4 S HA 4.394
4 S HN 8.291
4 S N 117.862
5 F CA 58.271
5 F CB 39.509
5 F HA 4.558
5 F HN 8.37
5 F N 119.239
6 G CA 45.301
6 G HA2 3.849
6 G HA3 3.945
6 G HN 8.453
6 G N 110.5
7 N CA 53.305
7 N CB 38.93
7 N HA 4.799
7 N HN 8.409
7 N N 119.051
8 T CA 61.945
8 T CB 69.566
8 T HA 4.37
8 T HN 8.349
8 T N 114.632
9 S CA 58.518
9 S CB 63.805
9 S HA 4.524
9 S HN 8.492
9 S N 118.335
10 T CA 61.946
10 T CB 69.652
10 T HA 4.371
10 T HN 8.381
10 T N 116.638
11 I CA 61.62
11 I CB 38.518
11 I HA 4.142
11 I HN 8.229
11 I N 123.612
12 G CA 45.079
12 G HA2 3.926
12 G HA3 3.926
12 G HN 8.66
12 G N 113.595
13 Q CA 53.38
13 Q CB 28.889
13 Q HA 4.647
13 Q HN 8.278
13 Q N 120.864
14 P CA 63.274
14 P CB 32.261
14 P HA 4.466
15 S CA 58.335
15 S CB 63.739
15 S HA 4.504
15 S HN 8.679
15 S N 116.69
16 T CA 61.889
16 T CB 69.73
16 T HA 4.368
16 T HN 8.364
16 T N 115.765
17 N CA 53.249
17 N CB 38.767
17 N HA 4.802
17 N HN 8.574
17 N N 121.26
18 T CA 62.228
18 T CB 69.512
18 T HA 4.317
18 T HN 8.34
18 T N 114.835
19 M CA 55.893
19 M CB 32.467
19 M HA 4.487
19 M HN 8.452
19 M N 122.341
20 G CA 45.327
20 G HA2 3.899
20 G HA3 3.899
20 G HN 8.434
20 G N 110.051
21 L CA 55.136
21 L CB 42.288
21 L HA 4.265
21 L HN 8.136
21 L N 121.51
22 F CA 57.699
22 F CB 39.446
22 F HA 4.608
22 F HN 8.421
22 F N 120.451
23 G CA 45.31
23 G HA2 3.87
23 G HA3 3.919
23 G HN 8.367
23 G N 110.597
24 V CA 62.542
24 V CB 32.854
24 V HA 4.189
24 V HN 8.067
24 V N 119.731
25 T CA 62.182
25 T CB 69.847
25 T HA 4.332
25 T HN 8.465
25 T N 119.289
26 Q CA 55.769
26 Q CB 29.591
26 Q HA 4.317
26 Q HN 8.604
26 Q N 123.831
27 A CA 52.622
27 A CB 19.219
27 A HA 4.314
27 A HN 8.55
27 A N 125.953
28 S CA 58.388
28 S CB 63.769
28 S HA 4.413
28 S HN 8.434
28 S N 115.671
29 Q CA 53.641
29 Q CB 28.951
29 Q HA 4.653
29 Q HN 8.49
29 Q N 122.947
30 P CA 63.166
30 P CB 32.344
30 P HA 4.373

Nup98$^{FG322\text{-}351}$
TQASQPGGLF
GTATNTSTGT
AFGTGTGLFG

1 T CA 62.211
1 T CB 69.828
1 T HA 4.283
1 T HN 8.359
1 T N 119.794
2 Q CA 55.926
2 Q CB 29.415
2 Q HA 4.326
2 Q HN 8.66
2 Q N 123.075
3 A CA 52.732
3 A CB 19.198
3 A HA 4.305
3 A HN 8.522
3 A N 125.338
4 S CA 58.376
4 S CB 63.789
4 S HA 4.413
4 S HN 8.395
4 S N 115.022
5 Q CA 53.624
5 Q CB 28.983

5 Q HA 4.664
5 Q HN 8.433
5 Q N 122.612
6 P CA 63.666
6 P CB 32.073
6 P HA 4.427
7 G CA 45.349
7 G HA2 3.98
7 G HA3 3.98
7 G HN 8.707
7 G N 109.768
8 G CA 45.289
8 G HA2 3.924
8 G HA3 3.924
8 G HN 8.336
8 G N 108.349
9 L CA 55.303
9 L CB 42.31
9 L HA 4.254
9 L HN 8.258
9 L N 121.354
10 F CA 57.834
10 F CB 39.505
10 F HA 4.637
10 F HN 8.429
10 F N 120.168
11 G CA 45.319
11 G HA2 3.968
11 G HA3 3.968
11 G HN 8.337
11 G N 110.37
12 T CA 61.894
12 T CB 70.007
12 T HA 4.344
12 T HN 8.169
12 T N 113.589
13 A CA 52.723
13 A CB 19.25
13 A HA 4.403
13 A HN 8.54
13 A N 126.495
14 T CA 61.912
14 T CB 69.9
14 T HA 4.316
14 T HN 8.3
14 T N 113.323
15 N CA 53.306
15 N CB 38.809
15 N HA 4.799
15 N HN 8.585
15 N N 121.173
16 T CA 62.009
16 T CB 69.74
16 T HA 4.382
16 T HN 8.331
16 T N 114.283
17 S CA 58.654
17 S CB 63.743
17 S HA 4.537
17 S HN 8.529
17 S N 118.132
18 T CA 62.098
18 T CB 69.74
18 T HA 4.378
18 T HN 8.314
18 T N 115.135
19 G CA 45.423
19 G HA2 4.025
19 G HA3 4.025
19 G HN 8.483
19 G N 111.002
20 T CA 61.806
20 T CB 69.981
20 T HA 4.312
20 T HN 8.178
20 T N 113.673
21 A CA 52.54
21 A CB 19.095
21 A HA 4.285
21 A HN 8.455
21 A N 126.24
22 F CA 58.08
22 F CB 39.623
22 F HA 4.582
22 F HN 8.357
22 F N 119.988
23 G CA 45.291
23 G HA2 3.886
23 G HA3 3.977
23 G HN 8.391
23 G N 112.173
24 T CA 62.021
24 T CB 69.886
24 T HA 4.369
24 T HN 8.235
24 T N 112.968
25 G CA 45.443
25 G HA2 4.025
25 G HA3 4.055
25 G HN 8.655
25 G N 111.318
26 T CA 61.991
26 T CB 69.884
26 T HA 4.365
26 T HN 8.241
26 T N 112.729
27 G CA 45.305
27 G HA2 3.896
27 G HA3 3.926
27 G HN 8.567
27 G N 110.987
28 L CA 55.204
28 L CB 42.315
28 L HA 4.26
28 L HN 8.163
28 L N 121.396
29 F CA 57.967
29 F CB 39.228
29 F HA 4.59
29 F HN 8.523
29 F N 120.937
30 G CA 44.937
30 G HA2 3.76
30 G HA3 3.846
30 G HN 8.394
30 G N 110.736

Nup98$^{\text{FG345-384}}$
TGTGLFGQTN
TGFGAVGSTL
FGNNKLTTFG
SSTTSAPSFG

1 T CA 61.697
1 T CB 69.727
1 T HA 4.306
1 T HN 8.416
1 T N 112.6
2 G CA 45.445
2 G HA2 4.027
2 G HA3 4.027
2 G HN 8.706
2 G N 111.817
3 T CA 61.866
3 T CB 69.793
3 T HA 4.374
3 T HN 8.239
3 T N 113.034
4 G CA 45.361
4 G HA2 3.921
4 G HA3 3.921
4 G HN 8.583
4 G N 111.272
5 L CA 55.219
5 L CB 42.272
5 L HA 4.265
5 L HN 8.162
5 L N 121.705
6 F CA 58.059
6 F CB 39.3
6 F HA 4.594
6 F HN 8.44
6 F N 120.727
7 G CA 45.235
7 G HA2 3.822
7 G HA3 3.905
7 G HN 8.395
7 G N 110.747

8 Q CA 55.797
8 Q CB 29.541
8 Q HA 4.404
8 Q HN 8.278
8 Q N 119.926
9 T CA 62.018
9 T CB 69.756
9 T HA 4.354
9 T HN 8.406
9 T N 115.547
10 N CA 53.291
10 N CB 38.773
10 N HA 4.799
10 N HN 8.671
10 N N 121.472
11 T CA 62.044
11 T CB 69.722
11 T HA 4.3
11 T HN 8.282
11 T N 114.007
12 G CA 45.29
12 G HA2 3.84
12 G HA3 3.918
12 G HN 8.452
12 G N 110.892
13 F CA 58.099
13 F CB 39.573
13 F HA 4.593
13 F HN 8.284
13 F N 120.453
14 G CA 45.204
14 G HA2 3.813
14 G HA3 3.878
14 G HN 8.495
14 G N 111.149
15 A CA 52.369
15 A CB 18.115
15 A HA 4.336
15 A HN 8.194
15 A N 124.039
16 V CA 62.848
16 V CB 32.608
16 V HA 4.053
16 V HN 8.362
16 V N 120.395
17 G CA 45.33
17 G HA2 3.956
17 G HA3 4.003
17 G HN 8.65
17 G N 113.17
18 S CA 58.462
18 S CB 63.915
18 S HA 4.486
18 S HN 8.307
18 S N 115.813
19 T CA 62.116
19 T CB 69.724
19 T HA 4.337
19 T HN 8.368
19 T N 116.423
20 L CA 55.31
20 L CB 42.225
20 L HA 4.272
20 L HN 8.233
20 L N 124.146
21 F CA 58.142
21 F CB 39.589
21 F HA 4.618
21 F HN 8.281
21 F N 120.491
22 G CA 45.301
22 G HA2 3.844
22 G HA3 3.914
22 G HN 8.377
22 G N 110.454
23 N CA 53.236
23 N CB 38.773
23 N HA 4.693
23 N HN 8.412
23 N N 119.779
24 N CA 53.502
24 N CB 38.72
24 N HA 4.658
24 N HN 8.516
24 N N 119.393
25 K CA 56.558
25 K CB 32.823
25 K HA 4.276
25 K HN 8.353
25 K N 121.427
26 L CA 55.271
26 L CB 42.334
26 L HA 4.419
26 L HN 8.335
26 L N 122.84
27 T CA 61.941
27 T CB 69.786
27 T HA 4.372
27 T HN 8.261
27 T N 115.228
28 T CA 62.321
28 T CB 69.78
28 T HA 4.306
28 T HN 8.136
28 T N 116.314
29 F CA 58.096
29 F CB 39.641
29 F HA 4.618
29 F HN 8.433
29 F N 123.032
30 G CA 45.268
30 G HA2 3.858
30 G HA3 3.962
30 G HN 8.47
30 G N 111.523
31 S CA 58.351
31 S CB 63.97
31 S HA 4.511
31 S HN 8.315
31 S N 115.874
32 S CA 58.538
32 S CB 63.815
32 S HA 4.568
32 S HN 8.612
32 S N 118.147
33 T CA 62.034
33 T CB 69.635
33 T HA 4.449
33 T HN 8.393
33 T N 116.036
34 T CA 61.856
34 T CB 69.768
34 T HA 4.395
34 T HN 8.265
34 T N 116.425
35 S CA 58.215
35 S CB 63.922
35 S HA 4.452
35 S HN 8.403
35 S N 118.851
36 A CA 50.67
36 A CB 19.292
36 A HA 4.591
36 A HN 8.444
36 A N 127.553
37 P CA 63.111
37 P CB 32.108
37 P HA 4.403
38 S CA 58.212
38 S CB 63.811
38 S HA 4.376
38 S HN 8.499
38 S N 116.329
39 F CA 57.804
39 F CB 39.574
39 F HA 4.614
39 F HN 8.475
39 F N 122.728
40 G CA 44.937
40 G HA2 3.771
40 G HA3 3.842
40 G HN 8.477
40 G N 112.584

Nup98$^{FG1-384}$

MFNKSFGTPF
GGGTGGFGTT

STFGQNTGFG
TTSGGAFGTS
AFGSSNNTGG
LFGNSQTKPG
GLFGTSSFSQ
PATSTSTGFG
FGTSTGTANT
LFGTASTGTS
LFSSQNNAFA
QNKPTGFGNF
GTSTSSGGLF
GTTNTTSNPF
GSTSGSLFGP
SSFTAAPTGT
TIKFNPPTGT
DTMVKAGVST
NISTKHQCIT
AMKEYESKSL
EELRLEDYQA
NRKGPQNQVG
AGTTTGLFGS
SPATSSATGL
FSSSTTNSGF
AYGQNKTAFG
TSTTGFGTNP
GGLFGQQNQQ
TTSLFSKPFG
QATTTQNTGF
SFGNTSTIGQ
PSTNTMGLFG
VTQASQPGGL
FGTATNTSTG
TAFGTGTGLF
GQTNTGFGAV
GSTLFGNNKL
TTFGSSTTSA
PSFG

1 M CA 55.148
1 M CB 33.364
1 M HA 3.97
2 F CA 57.943
2 F CB 39.642
2 F HA 4.637
2 F HN 8.284
2 F N 121.816
3 N CA 52.915
3 N CB 39.061
3 N HA 4.603
3 N HN 8.528
3 N N 122.099
4 K CA 56.784
4 K CB 42.058
4 K HA 4.192
4 K HN 8.419
4 K N 122.541
5 S CA 58.398
5 S CB 63.693
5 S HA 4.391
5 S HN 8.378
5 S N 116.589
6 F CA 58.058
6 F CB 39.648
6 F HA 4.615
6 F HN 8.327
6 F N 122.114
7 G CA 45.036
7 G HA2 3.893
7 G HA3 3.947
7 G HN 8.416
7 G N 110.356
8 T CA 59.827
8 T CB 69.759
8 T HA 4.613
8 T HN 8.183
8 T N 116.035
9 P CA 63.355
9 P CB 32.043
9 P HA 4.377
10 F CA 58.333
10 F CB 39.34
10 F HA 4.573
10 F HN 8.497
10 F N 120.952
11 G CA 45.303
11 G HA2 3.823
11 G HA3 3.874
11 G HN 8.417
11 G N 112
12 G CA 45.286
12 G HA2 3.925
12 G HA3 3.925
12 G HN 8.01
12 G N 108.431
13 G CA 45.211
13 G HA2 4.035
13 G HA3 4.035
13 G HN 8.411
13 G N 108.93
14 T CA 62.039
14 T CB 69.795
14 T HA 4.376
14 T HN 8.328
14 T N 113.057
15 G CA 45.286
15 G HA2 3.933
15 G HA3 3.933
15 G HN 8.612
15 G N 111.485
16 G CA 45.026
16 G HA2 3.858
16 G HA3 3.858
16 G HN 8.263
16 G N 108.624
17 F CA 58.16
17 F CB 39.504
17 F HA 4.599
17 F HN 8.327
17 F N 120.275
18 G CA 45.336
18 G HA2 3.887
18 G HA3 3.975
18 G HN 8.534
18 G N 111.129
19 T CA 61.844
19 T CB 70.016
19 T HA 4.463
19 T HN 8.189
19 T N 113.534
20 T CA 61.887
20 T CB 69.848
20 T HA 4.414
20 T HN 8.355
20 T N 116.345
21 S CA 58.396
21 S CB 63.882
21 S HA 4.513
21 S HN 8.501
21 S N 118.596
22 T CA 61.956
22 T CB 69.632
22 T HA 4.306
22 T HN 8.278
22 T N 115.958
23 F CA 58.167
23 F CB 39.509
23 F HA 4.565
23 F HN 8.333
23 F N 122.83
24 G CA 45.278
24 G HA2 3.755
24 G HA3 3.887
24 G HN 8.426
24 G N 111.484
25 Q CA 55.852
25 Q CB 29.47
25 Q HA 4.303
25 Q HN 8.244
25 Q N 119.725
26 N CA 53.317
26 N CB 38.68
26 N HA 4.77
26 N HN 8.71
26 N N 120.15
27 T CA 62.095
27 T CB 69.728
27 T HA 4.301
27 T HN 8.255

27 T N 114.055
28 G CA 45.303
28 G HA2 3.825
28 G HA3 3.908
28 G HN 8.46
28 G N 110.789
29 F CA 58.039
29 F CB 39.529
29 F HA 4.602
29 F HN 8.266
29 F N 120.414
30 G CA 45.336
30 G HA2 3.887
30 G HA3 3.975
30 G HN 8.534
30 G N 111.129
31 T CA 61.844
31 T CB 70.016
31 T HA 4.463
31 T HN 8.189
31 T N 113.534
32 T CA 61.904
32 T CB 70.016
32 T HA 4.452
32 T HN 8.397
32 T N 116.026
33 S CA 58.614
33 S CB 63.842
33 S HA 4.478
33 S HN 8.492
33 S N 118.264
34 G CA 45.359
34 G HA2 3.977
34 G HA3 3.977
34 G HN 8.544
34 G N 111.145
35 G CA 45.085
35 G HA2 3.901
35 G HA3 3.901
35 G HN 8.293
35 G N 108.828
36 A CA 52.479
36 A CB 19.138
36 A HA 4.245
36 A HN 8.301
36 A N 123.879
37 F CA 58.005
37 F CB 39.514
37 F HA 4.554
37 F HN 8.411
37 F N 119.678
38 G CA 45.328
38 G HA2 3.875
38 G HA3 3.972
38 G HN 8.325
38 G N 111.017
39 T CA 61.792
39 T CB 70.016
39 T HA 4.414
39 T HN 8.144
39 T N 113.318
40 S CA 58.462
40 S CB 63.815
40 S HA 4.424
40 S HN 8.506
40 S N 118.367
41 A CA 52.633
41 A CB 19.031
41 A HA 4.233
41 A HN 8.422
41 A N 126.071
42 F CA 58.12
42 F CB 39.432
42 F HA 4.545
42 F HN 8.279
42 F N 119.617
43 G CA 45.289
43 G HA2 3.829
43 G HA3 3.944
43 G HN 8.349
43 G N 111.204
44 S CA 58.449
44 S CB 63.936
44 S HA 4.473
44 S HN 8.277
44 S N 115.805
45 S CA 58.597
45 S CB 63.727
45 S HA 4.453
45 S HN 8.544
45 S N 117.844
46 N CA 53.312
46 N CB 38.711
46 N HA 4.701
46 N HN 8.511
46 N N 120.494
47 N CA 53.304
47 N CB 38.695
47 N HA 4.767
47 N HN 8.491
47 N N 119.483
48 T CA 62.232
48 T CB 69.645
48 T HA 4.333
48 T HN 8.291
48 T N 113.926
49 G CA 45.38
49 G HA2 3.975
49 G HA3 3.975
49 G HN 8.574
49 G N 111.331
50 G CA 45.199
50 G HA2 3.886
50 G HA3 3.886
50 G HN 8.318
50 G N 108.822
51 L CA 55.343
51 L CB 42.226
51 L HA 4.221
51 L HN 8.199
51 L N 121.736
52 F CA 57.733
52 F CB 39.394
52 F HA 4.615
52 F HN 8.409
52 F N 120.087
53 G CA 45.396
53 G HA2 3.856
53 G HA3 3.907
53 G HN 8.32
53 G N 110.278
54 N CA 53.22
54 N CB 39.005
54 N HA 4.759
54 N HN 8.422
54 N N 118.978
55 S CA 58.815
55 S CB 63.648
55 S HA 4.407
55 S HN 8.48
55 S N 116.438
56 Q CA 55.97
56 Q CB 29.446
56 Q HA 4.392
56 Q HN 8.556
56 Q N 121.965
57 T CA 62.131
57 T CB 69.781
57 T HA 4.278
57 T HN 8.254
57 T N 115.915
58 K CA 54.241
58 K CB 32.533
58 K HA 4.61
58 K HN 8.471
58 K N 125.719
59 P CA 63.493
59 P CB 32.136
59 P HA 4.415
60 G CA 45.456
60 G HA2 3.959
60 G HA3 3.987
60 G HN 8.673
60 G N 110.149
61 G CA 45.393
61 G HA2 3.894
61 G HA3 3.939
61 G HN 8.344

61 G N 108.732
62 L CA 55.267
62 L CB 42.314
62 L HA 4.265
62 L HN 8.287
62 L N 121.693
63 F CA 57.939
63 F CB 39.492
63 F HA 4.567
63 F HN 8.443
63 F N 120.715
64 G CA 45.456
64 G HA2 3.889
64 G HA3 3.978
64 G HN 8.313
64 G N 110.858
65 T CA 61.841
65 T CB 70.061
65 T HA 4.414
65 T HN 8.178
65 T N 113.337
66 S CA 58.564
66 S CB 63.825
66 S HA 4.497
66 S HN 8.56
66 S N 118.242
67 S CA 58.877
67 S CB 63.699
67 S HA 4.414
67 S HN 8.413
67 S N 117.908
68 F CA 57.856
68 F CB 39.62
68 F HA 4.636
68 F HN 8.262
68 F N 122.216
69 S CA 58.008
69 S CB 63.863
69 S HA 4.394
69 S HN 8.211
69 S N 118.103
70 Q CA 53.667
70 Q CB 28.972
70 Q HA 4.568
70 Q HN 8.417
70 Q N 123.474
71 P CA 63.058
71 P CB 32.207
71 P HA 4.402
72 A CA 52.615
72 A CB 19.161
72 A HA 4.35
72 A HN 8.667
72 A N 125.037
73 T CA 61.675
73 T CB 70.034
73 T HA 4.372
73 T HN 8.294
73 T N 113.448
74 S CA 58.368
74 S CB 63.823
74 S HA 4.53
74 S HN 8.499
74 S N 118.151
75 T CA 61.913
75 T CB 69.786
75 T HA 4.41
75 T HN 8.362
75 T N 115.779
76 S CA 58.472
76 S CB 63.745
76 S HA 4.498
76 S HN 8.446
76 S N 118.424
77 T CA 62.041
77 T CB 69.854
77 T HA 4.33
77 T HN 8.275
77 T N 115.545
78 G CA 45.052
78 G HA2 3.841
78 G HA3 3.938
78 G HN 8.396
78 G N 110.885
79 F CA 58.19
79 F CB 39.551
79 F HA 4.561
79 F HN 8.292
79 F N 120.415
80 G CA 45.057
80 G HA2 3.714
80 G HA3 3.852
80 G HN 8.435
80 G N 111.001
81 F CA 58.1
81 F CB 39.557
81 F HA 4.607
81 F HN 8.232
81 F N 120.417
82 G CA 45.365
82 G HA2 3.9
82 G HA3 3.996
82 G HN 8.565
82 G N 111.241
83 T CA 61.836
83 T CB 69.844
83 T HA 4.42
83 T HN 8.172
83 T N 113.524
84 S CA 58.415
84 S CB 63.687
84 S HA 4.56
84 S HN 8.593
84 S N 118.47
85 T CA 62.108
85 T CB 69.844
85 T HA 4.375
85 T HN 8.356
85 T N 115.665
86 G CA 45.391
86 G HA2 4.012
86 G HA3 4.012
86 G HN 8.486
86 G N 111.324
87 T CA 61.932
87 T CB 69.927
87 T HA 4.334
87 T HN 8.192
87 T N 114.013
88 A CA 52.815
88 A CB 19.172
88 A HA 4.301
88 A HN 8.511
88 A N 126.603
89 N CA 53.345
89 N CB 38.768
89 N HA 4.712
89 N HN 8.532
89 N N 118.253
90 T CA 62.097
90 T CB 69.807
90 T HA 4.27
90 T HN 8.177
90 T N 114.057
91 L CA 55.344
91 L CB 42.161
91 L HA 4.283
91 L HN 8.278
91 L N 124.2
92 F CA 57.956
92 F CB 39.558
92 F HA 4.617
92 F HN 8.3
92 F N 120.864
93 G CA 45.352
93 G HA2 3.899
93 G HA3 3.975
93 G HN 8.368
93 G N 110.846
94 T CA 61.898
94 T CB 69.95
94 T HA 4.336
94 T HN 8.174
94 T N 114.755
95 A CA 52.619
95 A CB 19.204
95 A HA 4.369
95 A HN 8.537

95 A N 126.78
96 S CA 58.439
96 S CB 63.773
96 S HA 4.5
96 S HN 8.495
96 S N 115.679
97 T CA 62.017
97 T CB 69.775
97 T HA 4.389
97 T HN 8.346
97 T N 115.646
98 G CA 45.447
98 G HA2 4.039
98 G HA3 4.039
98 G HN 8.555
98 G N 111.257
99 T CA 61.935
99 T CB 69.879
99 T HA 4.377
99 T HN 8.229
99 T N 113.941
100 S CA 58.45
100 S CB 63.749
100 S HA 4.452
100 S HN 8.523
100 S N 118.78
101 L CA 55.562
101 L CB 42.142
101 L HA 4.274
101 L HN 8.417
101 L N 124.461
102 F CA 57.974
102 F CB 39.669
102 F HA 4.621
102 F HN 8.303
102 F N 120.449
103 S CA 58.344
103 S CB 63.819
103 S HA 4.435
103 S HN 8.251
103 S N 117.345
104 S CA 58.883
104 S CB 63.631
104 S HA 4.417
104 S HN 8.494
104 S N 118.378
105 Q CA 56.203
105 Q CB 29.275
105 Q HA 4.306
105 Q HN 8.425
105 Q N 121.699
106 N CA 53.322
106 N CB 38.742
106 N HA 4.664
106 N HN 8.431
106 N N 119.555
107 N CA 53.372
107 N CB 38.774
107 N HA 4.654
107 N HN 8.44
107 N N 119.728
108 A CA 53.181
108 A CB 18.839
108 A HA 4.181
108 A HN 8.314
108 A N 124.125
109 F CA 57.731
109 F CB 39.218
109 F HA 4.572
109 F HN 8.167
109 F N 118.624
110 A CA 52.644
110 A CB 19.173
110 A HA 4.245
110 A HN 8.104
110 A N 124.857
111 Q CA 55.866
111 Q CB 29.354
111 Q HA 4.266
111 Q HN 8.351
111 Q N 119.131
112 N CA 52.965
112 N CB 38.794
112 N HA 4.669
112 N HN 8.492
112 N N 119.672
113 K CA 54.251
113 K CB 32.498
113 K HA 4.576
113 K HN 8.351
113 K N 122.84
114 P CA 63.124
114 P CB 32.164
114 P HA 4.487
115 T CA 62.171
115 T CB 69.908
115 T HA 4.281
115 T HN 8.448
115 T N 114.782
116 G CA 45.111
116 G HA2 3.854
116 G HA3 3.947
116 G HN 8.465
116 G N 111.133
117 F CA 58.048
117 F CB 39.505
117 F HA 4.607
117 F HN 8.303
117 F N 120.27
118 G CA 45.205
118 G HA2 3.729
118 G HA3 3.851
118 G HN 8.458
118 G N 111.023
119 N CA 53.033
119 N CB 38.72
119 N HA 4.675
119 N HN 8.267
119 N N 118.797
120 F CA 58.033
120 F CB 39.374
120 F HA 4.635
120 F HN 8.432
120 F N 120.971
121 G CA 45.368
121 G HA2 3.928
121 G HA3 3.99
121 G HN 8.46
121 G N 110.566
122 T CA 61.768
122 T CB 69.981
122 T HA 4.424
122 T HN 8.179
122 T N 113.405
123 S CA 58.577
123 S CB 63.873
123 S HA 4.564
123 S HN 8.563
123 S N 118.356
124 T CA 61.918
124 T CB 69.75
124 T HA 4.422
124 T HN 8.385
124 T N 115.744
125 S CA 58.497
125 S CB 63.827
125 S HA 4.506
125 S HN 8.439
125 S N 118.168
126 S CA 58.668
126 S CB 63.822
126 S HA 4.481
126 S HN 8.505
126 S N 118.115
127 G CA 45.407
127 G HA2 3.98
127 G HA3 3.98
127 G HN 8.515
127 G N 110.987
128 G CA 45.175
128 G HA2 3.906
128 G HA3 3.906
128 G HN 8.299
128 G N 108.726
129 L CA 55.275
129 L CB 42.292
129 L HA 4.256
129 L HN 8.213

129 L N 121.712
130 F CA 57.921
130 F CB 39.462
130 F HA 4.626
130 F HN 8.432
130 F N 120.593
131 G CA 45.368
131 G HA2 3.929
131 G HA3 3.976
131 G HN 8.336
131 G N 110.636
132 T CA 61.807
132 T CB 70.018
132 T HA 4.457
132 T HN 8.185
132 T N 113.41
133 T CA 61.952
133 T CB 69.866
133 T HA 4.39
133 T HN 8.352
133 T N 116.282
134 N CA 53.346
134 N CB 38.873
134 N HA 4.806
134 N HN 8.644
134 N N 121.778
135 T CA 61.869
135 T CB 69.738
135 T HA 4.426
135 T HN 8.349
135 T N 114.773
136 T CA 62.082
136 T CB 69.816
136 T HA 4.379
136 T HN 8.312
136 T N 116.228
137 S CA 58.428
137 S CB 63.739
137 S HA 4.428
137 S HN 8.409
137 S N 118.239
138 N CB 38.884
138 N HA 4.971
138 N HN 8.528
138 N N 121.452
139 P CA 63.682
139 P CB 31.901
139 P HA 4.334
140 F CA 57.87
140 F CB 39.02
140 F HA 4.634
140 F HN 8.334
140 F N 119.545
141 G CA 45.383
141 G HA2 3.898
141 G HA3 3.969
141 G HN 8.16
141 G N 110.079
142 S CA 58.408
142 S CB 63.958
142 S HA 4.547
142 S HN 8.343
142 S N 115.401
143 T CA 61.837
143 T CB 69.742
143 T HA 4.453
143 T HN 8.459
143 T N 117.826
144 S CA 58.808
144 S CB 63.738
144 S HA 4.448
144 S HN 8.46
144 S N 115.486
145 G CA 45.35
145 G HA2 3.982
145 G HA3 3.982
145 G HN 8.548
145 G N 110.973
146 S CA 58.368
146 S CB 63.842
146 S HA 4.415
146 S HN 8.262
146 S N 115.374
147 L CA 55.446
147 L CB 42.166
147 L HA 4.262
147 L HN 8.37
147 L N 123.868
148 F CA 57.377
148 F CB 39.886
148 F HA 4.697
148 F HN 8.237
148 F N 119.508
149 G CA 44.646
149 G HA2 4.059
149 G HA3 4.093
149 G HN 8.228
149 G N 110.081
150 P CA 63.501
150 P CB 32.168
150 P HA 4.427
151 S CA 58.515
151 S CB 63.765
151 S HA 4.467
151 S HN 8.613
151 S N 115.643
152 S CA 58.568
152 S CB 63.825
152 S HA 4.403
152 S HN 8.341
152 S N 117.49
153 F CA 57.851
153 F CB 39.742
153 F HA 4.685
153 F HN 8.284
153 F N 121.853
154 T CA 61.627
154 T CB 70.004
154 T HA 4.242
154 T HN 8.124
154 T N 117.146
155 A CA 52.14
155 A CB 19.317
155 A HA 4.25
155 A HN 8.372
155 A N 127.251
156 A CA 50.481
156 A CB 17.929
156 A HA 4.546
156 A HN 8.44
156 A N 125.13
157 P CA 63.064
157 P CB 32.108
157 P HA 4.497
158 T CA 62.047
158 T CB 69.962
158 T HA 4.341
158 T HN 8.456
158 T N 114.504
159 G CA 45.337
159 G HA2 4.037
159 G HA3 4.037
159 G HN 8.592
159 G N 111.172
160 T CA 61.932
160 T CB 69.786
160 T HA 4.423
160 T HN 8.239
160 T N 113.872
161 T CA 62.138
161 T CB 69.848
161 T HA 4.348
161 T HN 8.389
161 T N 117.666
162 I CA 61.104
162 I CB 38.562
162 I HA 4.1
162 I HN 8.365
162 I N 124.575
163 K CA 55.966
163 K CB 33.244
163 K HA 4.285
163 K HN 8.482
163 K N 126.4
164 F CA 57.419
164 F CB 39.921
164 F HA 4.615
164 F HN 8.461

164 F N 122.747
165 N CB 39.034
165 N HA 4.948
165 N HN 8.53
165 N N 123.627
166 P CA 61.488
166 P CB 30.894
166 P HA 4.544
167 P CA 63.047
167 P CB 32.093
167 P HA 4.5
168 T CA 62.101
168 T CB 70.027
168 T HA 4.358
168 T HN 8.48
168 T N 114.75
169 G CA 45.398
169 G HA2 4.062
169 G HA3 4.062
169 G HN 8.614
169 G N 111.492
170 T CA 61.885
170 T CB 69.651
170 T HA 4.361
170 T HN 8.277
170 T N 113.71
171 D CA 54.586
171 D CB 40.986
171 D HA 4.678
171 D HN 8.6
171 D N 122.964
172 T CA 62.305
172 T CB 69.542
172 T HA 4.284
172 T HN 8.218
172 T N 114.606
173 M CA 55.765
173 M CB 32.646
173 M HA 4.475
173 M HN 8.43
173 M N 122.962
174 V CA 62.669
174 V CB 32.635
174 V HA 4.042
174 V HN 8.262
174 V N 120.494
175 K CA 56.116
175 K CB 33.119
175 K HA 4.312
175 K HN 8.557
175 K N 126.5
176 A CA 52.666
176 A CB 19.208
176 A HA 4.292
176 A HN 8.526
176 A N 126.354
177 G CA 45.181
177 G HA2 3.953
177 G HA3 4.001
177 G HN 8.538
177 G N 108.833
178 V CA 62.243
178 V CB 32.92
178 V HA 4.191
178 V HN 8.127
178 V N 119.671
179 S CA 58.335
179 S CB 63.62
179 S HA 4.563
179 S HN 8.676
179 S N 120.24
180 T CA 61.963
180 T CB 69.733
180 T HA 4.36
180 T HN 8.384
180 T N 116.293
181 N CA 53.364
181 N CB 38.76
181 N HA 4.743
181 N HN 8.559
181 N N 121.577
182 I CA 61.369
182 I CB 38.678
182 I HA 4.211
182 I HN 8.29
182 I N 121.675
183 S CA 58.49
183 S CB 63.753
183 S HA 4.529
183 S HN 8.593
183 S N 120.226
184 T CA 62.098
184 T CB 69.684
184 T HA 4.322
184 T HN 8.351
184 T N 116.733
185 K CA 55.921
185 K CB 32.899
185 K HA 4.264
185 K HN 8.336
185 K N 123.575
186 H CA 55.612
186 H CB 29.378
186 H HA 4.661
186 H HN 8.625
186 H N 120.294
187 Q CA 55.929
187 Q CB 29.649
187 Q HA 4.334
187 Q HN 8.594
187 Q N 122.562
188 C CA 58.641
188 C CB 27.892
188 C HA 4.52
188 C HN 8.684
188 C N 122.012
189 I CA 61.67
189 I CB 38.633
189 I HA 4.244
189 I HN 8.574
189 I N 122.123
190 T CA 62.248
190 T CB 69.684
190 T HA 4.271
190 T HN 8.321
190 T N 119.07
191 A CA 52.691
191 A CB 19.147
191 A HA 4.321
191 A HN 8.418
191 A N 126.824
192 M CA 55.776
192 M CB 32.824
192 M HA 4.425
192 M HN 8.406
192 M N 120.194
193 K CA 56.585
193 K CB 32.939
193 K HA 4.25
193 K HN 8.47
193 K N 123.149
194 E CA 57.065
194 E CB 30.106
194 E HA 4.2
194 E HN 8.574
194 E N 124.499
195 Y CA 58.38
195 Y CB 38.737
195 Y HA 4.506
195 Y HN 8.263
195 Y N 122.832
196 E CA 56.646
196 E CB 30.492
196 E HA 4.248
196 E HN 8.302
196 E N 122.819
197 S CA 58.823
197 S CB 63.761
197 S HA 4.382
197 S HN 8.431
197 S N 117.409
198 K CA 56.634
198 K CB 33.175
198 K HA 4.364
198 K HN 8.456
198 K N 123.725
199 S CA 58.605
199 S CB 63.882

199 S HA 4.397
199 S HN 8.536
199 S N 117.303
200 L CA 56.992
200 L CB 41.796
200 L HA 4.193
200 L HN 8.555
200 L N 122.592
201 E CA 58.259
201 E CB 29.806
201 E HA 4.14
201 E HN 8.452
201 E N 119.631
202 E CA 58.13
202 E CB 30.099
202 E HA 4.105
202 E HN 8.169
202 E N 120.808
203 L CA 56.658
203 L CB 41.991
203 L HA 4.172
203 L HN 8.276
203 L N 121.726
204 R CA 56.545
204 R CB 30.434
204 R HA 4.253
204 R HN 8.316
204 R N 121.188
205 L CA 56.64
205 L CB 42.005
205 L HA 4.246
205 L HN 8.185
205 L N 122.032
206 E CA 58.14
206 E CB 30.462
206 E HA 4.146
206 E HN 8.364
206 E N 119.793
207 D CA 55.562
207 D CB 40.969
207 D HA 4.534
207 D HN 8.201
207 D N 120.394
208 Y CA 59.677
208 Y CB 38.32
208 Y HA 4.388
208 Y HN 8.181
208 Y N 120.768
209 Q CA 56.664
209 Q CB 28.997
209 Q HA 4.097
209 Q HN 8.257
209 Q N 119.957
210 A CA 53.565
210 A CB 18.833
210 A HA 4.161
210 A HN 8.112
210 A N 122.814
211 N CA 53.48
211 N CB 38.713
211 N HA 4.648
211 N HN 8.208
211 N N 115.603
212 R CA 56.664
212 R CB 30.516
212 R HA 4.262
212 R HN 8.061
212 R N 120.543
213 K CA 56.48
213 K CB 33.24
213 K HA 4.378
213 K HN 8.374
213 K N 121.821
214 G CA 44.62
214 G HA2 4.084
214 G HA3 4.124
214 G HN 8.338
214 G N 109.731
215 P CA 63.439
215 P CB 32.151
215 P HA 4.418
216 Q CA 55.783
216 Q CB 29.488
216 Q HA 4.312
216 Q HN 8.773
216 Q N 120.764
217 N CA 53.384
217 N CB 38.819
217 N HA 4.669
217 N HN 8.541
217 N N 119.658
218 Q CA 55.808
218 Q CB 29.514
218 Q HA 4.361
218 Q HN 8.478
218 Q N 120.94
219 V CA 62.79
219 V CB 32.645
219 V HA 4.088
219 V HN 8.403
219 V N 122.421
220 G CA 45.215
220 G HA2 3.955
220 G HA3 3.955
220 G HN 8.679
220 G N 113.414
221 A CA 52.749
221 A CB 19.351
221 A HA 4.327
221 A HN 8.387
221 A N 124.283
222 G CA 45.344
222 G HA2 4.002
222 G HA3 4.002
222 G HN 8.612
222 G N 108.576
223 T CA 61.885
223 T CB 69.919
223 T HA 4.461
223 T HN 8.202
223 T N 113.741
224 T CA 61.903
224 T CB 69.799
224 T HA 4.485
224 T HN 8.452
224 T N 116.472
225 T CA 62.108
225 T CB 69.868
225 T HA 4.361
225 T HN 8.287
225 T N 116.318
226 G CA 45.307
226 G HA2 3.927
226 G HA3 3.927
226 G HN 8.486
226 G N 111.344
227 L CA 55.21
227 L CB 42.356
227 L HA 4.272
227 L HN 8.177
227 L N 121.662
228 F CA 57.996
228 F CB 39.37
228 F HA 4.611
228 F HN 8.506
228 F N 121.104
229 G CA 45.267
229 G HA2 3.878
229 G HA3 3.955
229 G HN 8.394
229 G N 111.067
230 S CA 58.276
230 S CB 64.066
230 S HA 4.503
230 S HN 8.264
230 S N 115.698
231 S CA 56.597
231 S CB 63.326
231 S HA 4.804
231 S HN 8.516
231 S N 119.092
232 P CA 63.351
232 P CB 32.134
232 P HA 4.422
233 A CA 52.744
233 A CB 19.148
233 A HA 4.341
233 A HN 8.556

233 A N 124.408
234 T CA 61.743
234 T CB 69.913
234 T HA 4.373
234 T HN 8.235
234 T N 113.246
235 S CA 58.298
235 S CB 63.852
235 S HA 4.525
235 S HN 8.423
235 S N 118.123
236 S CA 58.477
236 S CB 63.815
236 S HA 4.472
236 S HN 8.53
236 S N 118.35
237 A CA 52.919
237 A CB 19.156
237 A HA 4.399
237 A HN 8.507
237 A N 126.431
238 T CA 62.054
238 T CB 69.894
238 T HA 4.331
238 T HN 8.188
238 T N 112.501
239 G CA 45.335
239 G HA2 3.925
239 G HA3 3.925
239 G HN 8.39
239 G N 111.073
240 L CA 55.193
240 L CB 42.348
240 L HA 4.273
240 L HN 8.125
240 L N 121.643
241 F CA 57.668
241 F CB 39.592
241 F HA 4.66
241 F HN 8.415
241 F N 120.972
242 S CA 58.118
242 S CB 63.964
242 S HA 4.455
242 S HN 8.275
242 S N 117.617
243 S CA 58.513
243 S CB 63.811
243 S HA 4.493
243 S HN 8.53
243 S N 118.35
244 S CA 58.64
244 S CB 63.802
244 S HA 4.543
244 S HN 8.495
244 S N 117.919
245 T CA 62.09
245 T CB 69.688
245 T HA 4.437
245 T HN 8.328
245 T N 115.812
246 T CA 62.154
246 T CB 69.783
246 T HA 4.343
246 T HN 8.229
246 T N 116.229
247 N CA 53.323
247 N CB 38.8
247 N HA 4.74
247 N HN 8.571
247 N N 121.524
248 S CA 58.893
248 S CB 63.732
248 S HA 4.378
248 S HN 8.441
248 S N 116.645
249 G CA 45.279
249 G HA2 3.853
249 G HA3 3.916
249 G HN 8.48
249 G N 110.585
250 F CA 57.875
250 F CB 39.729
250 F HA 4.541
250 F HN 8.115
250 F N 120.352
251 A CA 52.263
251 A CB 19.159
251 A HA 4.255
251 A HN 8.295
251 A N 125.682
252 Y CA 58.556
252 Y CB 38.605
252 Y HA 4.442
252 Y HN 8.161
252 Y N 119.881
253 G CA 45.385
253 G HA2 3.795
253 G HA3 3.916
253 G HN 8.413
253 G N 110.958
254 Q CA 55.907
254 Q CB 29.41
254 Q HA 4.285
254 Q HN 8.237
254 Q N 119.688
255 N CA 53.36
255 N CB 38.688
255 N HA 4.638
255 N HN 8.597
255 N N 119.68
256 K CA 56.565
256 K CB 32.997
256 K HA 4.323
256 K HN 8.427
256 K N 122.052
257 T CA 61.897
257 T CB 69.991
257 T HA 4.276
257 T HN 8.249
257 T N 115.227
258 A CA 52.481
258 A CB 19.162
258 A HA 4.256
258 A HN 8.378
258 A N 126.335
259 F CA 58.053
259 F CB 39.596
259 F HA 4.593
259 F HN 8.321
259 F N 119.96
260 G CA 45.299
260 G HA2 3.895
260 G HA3 3.969
260 G HN 8.39
260 G N 111.073
261 T CA 61.808
261 T CB 70.038
261 T HA 4.403
261 T HN 8.161
261 T N 113.311
262 S CA 58.42
262 S CB 63.81
262 S HA 4.565
262 S HN 8.582
262 S N 118.071
263 T CA 62.014
263 T CB 69.598
263 T HA 4.434
263 T HN 8.467
263 T N 115.878
264 T CA 62.092
264 T CB 69.835
264 T HA 4.339
264 T HN 8.215
264 T N 115.414
265 G CA 45.162
265 G HA2 3.835
265 G HA3 3.926
265 G HN 8.429
265 G N 110.844
266 F CA 58.104
266 F CB 39.616
266 F HA 4.618
266 F HN 8.321
266 F N 120.087
267 G CA 45.304
267 G HA2 3.875

267 G HA3 3.976
267 G HN 8.543
267 G N 110.816
268 T CA 61.827
268 T CB 69.876
268 T HA 4.319
268 T HN 8.144
268 T N 112.978
269 N CB 38.743
269 N HA 4.985
269 N HN 8.694
269 N N 121.566
270 P CA 63.96
270 P CB 31.923
270 P HA 4.402
271 G CA 45.273
271 G HA2 3.94
271 G HA3 3.94
271 G HN 8.608
271 G N 109.209
272 G CA 45.332
272 G HA2 3.908
272 G HA3 3.908
272 G HN 8.216
272 G N 108.621
273 L CA 55.342
273 L CB 42.239
273 L HA 4.255
273 L HN 8.2
273 L N 121.602
274 F CA 58.065
274 F CB 39.455
274 F HA 4.598
274 F HN 8.434
274 F N 120.515
275 G CA 45.338
275 G HA2 3.837
275 G HA3 3.919
275 G HN 8.361
275 G N 110.542
276 Q CA 56.088
276 Q CB 29.341
276 Q HA 4.305
276 Q HN 8.317
276 Q N 119.905
277 Q CA 56.088
277 Q CB 29.285
277 Q HA 4.303
277 Q HN 8.642
277 Q N 121.414
278 N CA 53.469
278 N CB 38.704
278 N HA 4.672
278 N HN 8.591
278 N N 119.7
279 Q CA 56.088
279 Q CB 29.341
279 Q HA 4.304
279 Q HN 8.511
279 Q N 120.79
280 Q CA 56.086
280 Q CB 29.366
280 Q HA 4.38
280 Q HN 8.594
280 Q N 121.529
281 T CA 62.055
281 T CB 69.766
281 T HA 4.403
281 T HN 8.394
281 T N 115.84
282 T CA 62.031
282 T CB 69.766
282 T HA 4.364
282 T HN 8.286
282 T N 116.664
283 S CA 58.405
283 S CB 63.755
283 S HA 4.436
283 S HN 8.478
283 S N 118.608
284 L CA 55.342
284 L CB 42.172
284 L HA 4.254
284 L HN 8.356
284 L N 122.579
285 F CA 57.535
285 F CB 39.541
285 F HA 4.652
285 F HN 8.233
285 F N 120.14
286 S CA 58.105
286 S CB 63.869
286 S HA 4.401
286 S HN 8.167
286 S N 117.626
287 K CA 54.384
287 K CB 32.579
287 K HA 4.56
287 K HN 8.368
287 K N 119.714
288 P CA 63.169
288 P CB 32.052
288 P HA 4.382
289 F CA 58.361
289 F CB 39.528
289 F HA 4.553
289 F HN 8.356
289 F N 122.579
290 G CA 45.228
290 G HA2 3.792
290 G HA3 3.866
290 G HN 8.398
290 G N 110.266
291 Q CA 55.664
291 Q CB 29.716
291 Q HA 4.316
291 Q HN 8.233
291 Q N 120.14
292 A CA 52.637
292 A CB 19.235
292 A HA 4.378
292 A HN 8.58
292 A N 126.059
293 T CA 61.83
293 T CB 69.766
293 T HA 4.414
293 T HN 8.383
293 T N 114.079
294 T CA 61.712
294 T CB 69.89
294 T HA 4.462
294 T HN 8.337
294 T N 116.343
295 T CA 62.022
295 T CB 69.783
295 T HA 4.329
295 T HN 8.324
295 T N 116.718
296 Q CA 55.877
296 Q CB 29.499
296 Q HA 4.333
296 Q HN 8.549
296 Q N 123.004
297 N CA 53.232
297 N CB 38.745
297 N HA 4.754
297 N HN 8.663
297 N N 120.671
298 T CA 62.073
298 T CB 69.766
298 T HA 4.312
298 T HN 8.296
298 T N 114.386
299 G CA 45.215
299 G HA2 3.86
299 G HA3 3.933
299 G HN 8.507
299 G N 110.971
300 F CA 57.877
300 F CB 39.78
300 F HA 4.585
300 F HN 8.2
300 F N 120.426
301 S CA 57.918
301 S CB 63.792
301 S HA 4.382
301 S HN 8.26
301 S N 117.91

302 F CA 58.271
302 F CB 39.509
302 F HA 4.558
302 F HN 8.37
302 F N 119.239
303 G CA 45.301
303 G HA2 3.849
303 G HA3 3.945
303 G HN 8.453
303 G N 110.5
304 N CA 53.305
304 N CB 38.93
304 N HA 4.799
304 N HN 8.409
304 N N 119.051
305 T CA 61.945
305 T CB 69.566
305 T HA 4.37
305 T HN 8.349
305 T N 114.632
306 S CA 58.518
306 S CB 63.805
306 S HA 4.524
306 S HN 8.492
306 S N 118.335
307 T CA 61.946
307 T CB 69.652
307 T HA 4.371
307 T HN 8.381
307 T N 116.638
308 I CA 61.62
308 I CB 38.518
308 I HA 4.142
308 I HN 8.229
308 I N 123.612
309 G CA 45.079
309 G HA2 3.926
309 G HA3 3.926
309 G HN 8.66
309 G N 113.595
310 Q CA 53.38
310 Q CB 28.889
310 Q HA 4.647
310 Q HN 8.278
310 Q N 120.864
311 P CA 63.274
311 P CB 32.261
311 P HA 4.466
312 S CA 58.335
312 S CB 63.739
312 S HA 4.504
312 S HN 8.679
312 S N 116.69
313 T CA 61.889
313 T CB 69.73
313 T HA 4.368
313 T HN 8.364
313 T N 115.765
314 N CA 53.249
314 N CB 38.767
314 N HA 4.802
314 N HN 8.574
314 N N 121.26
315 T CA 62.228
315 T CB 69.512
315 T HA 4.317
315 T HN 8.34
315 T N 114.835
316 M CA 55.893
316 M CB 32.467
316 M HA 4.487
316 M HN 8.452
316 M N 122.341
317 G CA 45.327
317 G HA2 3.899
317 G HA3 3.899
317 G HN 8.434
317 G N 110.051
318 L CA 55.136
318 L CB 42.288
318 L HA 4.265
318 L HN 8.136
318 L N 121.51
319 F CA 57.699
319 F CB 39.446
319 F HA 4.608
319 F HN 8.421
319 F N 120.451
320 G CA 45.31
320 G HA2 3.87
320 G HA3 3.919
320 G HN 8.367
320 G N 110.597
321 V CA 62.542
321 V CB 32.854
321 V HA 4.189
321 V HN 8.067
321 V N 119.731
322 T CA 62.182
322 T CB 69.847
322 T HA 4.332
322 T HN 8.465
322 T N 119.289
323 Q CA 55.769
323 Q CB 29.591
323 Q HA 4.317
323 Q HN 8.604
323 Q N 123.831
324 A CA 52.622
324 A CB 19.219
324 A HA 4.314
324 A HN 8.55
324 A N 125.953
325 S CA 58.376
325 S CB 63.789
325 S HA 4.413
325 S HN 8.395
325 S N 115.022
326 Q CA 53.624
326 Q CB 28.983
326 Q HA 4.664
326 Q HN 8.433
326 Q N 122.612
327 P CA 63.666
327 P CB 32.073
327 P HA 4.427
328 G CA 45.349
328 G HA2 3.98
328 G HA3 3.98
328 G HN 8.707
328 G N 109.768
329 G CA 45.289
329 G HA2 3.924
329 G HA3 3.924
329 G HN 8.336
329 G N 108.349
330 L CA 55.303
330 L CB 42.31
330 L HA 4.254
330 L HN 8.258
330 L N 121.354
331 F CA 57.834
331 F CB 39.505
331 F HA 4.637
331 F HN 8.429
331 F N 120.168
332 G CA 45.319
332 G HA2 3.968
332 G HA3 3.968
332 G HN 8.337
332 G N 110.37
333 T CA 61.894
333 T CB 70.007
333 T HA 4.344
333 T HN 8.169
333 T N 113.589
334 A CA 52.723
334 A CB 19.25
334 A HA 4.403
334 A HN 8.54
334 A N 126.495
335 T CA 61.912
335 T CB 69.9
335 T HA 4.316
335 T HN 8.3
335 T N 113.323
336 N CA 53.306
336 N CB 38.809

336 N HA 4.799
336 N HN 8.585
336 N N 121.173
337 T CA 62.009
337 T CB 69.74
337 T HA 4.382
337 T HN 8.331
337 T N 114.283
338 S CA 58.654
338 S CB 63.743
338 S HA 4.537
338 S HN 8.529
338 S N 118.132
339 T CA 62.098
339 T CB 69.74
339 T HA 4.378
339 T HN 8.314
339 T N 115.135
340 G CA 45.423
340 G HA2 4.025
340 G HA3 4.025
340 G HN 8.483
340 G N 111.002
341 T CA 61.806
341 T CB 69.981
341 T HA 4.312
341 T HN 8.178
341 T N 113.673
342 A CA 52.54
342 A CB 19.095
342 A HA 4.285
342 A HN 8.455
342 A N 126.24
343 F CA 58.08
343 F CB 39.623
343 F HA 4.582
343 F HN 8.357
343 F N 119.988
344 G CA 45.291
344 G HA2 3.886
344 G HA3 3.977
344 G HN 8.391
344 G N 112.173
345 T CA 62.021
345 T CB 69.886
345 T HA 4.369
345 T HN 8.235
345 T N 112.968
346 G CA 45.443
346 G HA2 4.025
346 G HA3 4.055
346 G HN 8.655
346 G N 111.318
347 T CA 61.991
347 T CB 69.884
347 T HA 4.365
347 T HN 8.241
347 T N 112.729
348 G CA 45.305
348 G HA2 3.896
348 G HA3 3.926
348 G HN 8.567
348 G N 110.987
349 L CA 55.219
349 L CB 42.272
349 L HA 4.265
349 L HN 8.162
349 L N 121.705
350 F CA 58.059
350 F CB 39.3
350 F HA 4.594
350 F HN 8.44
350 F N 120.727
351 G CA 45.235
351 G HA2 3.822
351 G HA3 3.905
351 G HN 8.395
351 G N 110.747
352 Q CA 55.797
352 Q CB 29.541
352 Q HA 4.404
352 Q HN 8.278
352 Q N 119.926
353 T CA 62.018
353 T CB 69.756
353 T HA 4.354
353 T HN 8.406
353 T N 115.547
354 N CA 53.291
354 N CB 38.773
354 N HA 4.799
354 N HN 8.671
354 N N 121.472
355 T CA 62.044
355 T CB 69.722
355 T HA 4.3
355 T HN 8.282
355 T N 114.007
356 G CA 45.29
356 G HA2 3.84
356 G HA3 3.918
356 G HN 8.452
356 G N 110.892
357 F CA 58.099
357 F CB 39.573
357 F HA 4.593
357 F HN 8.284
357 F N 120.453
358 G CA 45.204
358 G HA2 3.813
358 G HA3 3.878
358 G HN 8.495
358 G N 111.149
359 A CA 52.369
359 A CB 18.115
359 A HA 4.336
359 A HN 8.194
359 A N 124.039
360 V CA 62.848
360 V CB 32.608
360 V HA 4.053
360 V HN 8.362
360 V N 120.395
361 G CA 45.33
361 G HA2 3.956
361 G HA3 4.003
361 G HN 8.65
361 G N 113.17
362 S CA 58.462
362 S CB 63.915
362 S HA 4.486
362 S HN 8.307
362 S N 115.813
363 T CA 62.116
363 T CB 69.724
363 T HA 4.337
363 T HN 8.368
363 T N 116.423
364 L CA 55.31
364 L CB 42.225
364 L HA 4.272
364 L HN 8.233
364 L N 124.146
365 F CA 58.142
365 F CB 39.589
365 F HA 4.618
365 F HN 8.281
365 F N 120.491
366 G CA 45.301
366 G HA2 3.844
366 G HA3 3.914
366 G HN 8.377
366 G N 110.454
367 N CA 53.236
367 N CB 38.773
367 N HA 4.693
367 N HN 8.412
367 N N 119.779
368 N CA 53.502
368 N CB 38.72
368 N HA 4.658
368 N HN 8.516
368 N N 119.393
369 K CA 56.558
369 K CB 32.823
369 K HA 4.276
369 K HN 8.353
369 K N 121.427

370 L CA 55.271
370 L CB 42.334
370 L HA 4.419
370 L HN 8.335
370 L N 122.84
371 T CA 61.941
371 T CB 69.786
371 T HA 4.372
371 T HN 8.261
371 T N 115.228
372 T CA 62.321
372 T CB 69.78
372 T HA 4.306
372 T HN 8.136
372 T N 116.314
373 F CA 58.096
373 F CB 39.641
373 F HA 4.618
373 F HN 8.433
373 F N 123.032
374 G CA 45.268
374 G HA2 3.858
374 G HA3 3.962
374 G HN 8.47
374 G N 111.523
375 S CA 58.351
375 S CB 63.97
375 S HA 4.511
375 S HN 8.315
375 S N 115.874
376 S CA 58.538
376 S CB 63.815
376 S HA 4.568
376 S HN 8.612
376 S N 118.147
377 T CA 62.034
377 T CB 69.635
377 T HA 4.449
377 T HN 8.393
377 T N 116.036
378 T CA 61.856
378 T CB 69.768
378 T HA 4.395
378 T HN 8.265
378 T N 116.425
379 S CA 58.215
379 S CB 63.922
379 S HA 4.452
379 S HN 8.403
379 S N 118.851
380 A CA 50.67
380 A CB 19.292
380 A HA 4.591
380 A HN 8.444
380 A N 127.553
381 P CA 63.111
381 P CB 32.108
381 P HA 4.403
382 S CA 58.212
382 S CB 63.811
382 S HA 4.376
382 S HN 8.499
382 S N 116.329
383 F CA 57.804
383 F CB 39.574
383 F HA 4.614
383 F HN 8.475
383 F N 122.728
384 G CA 44.937
384 G HA2 3.771
384 G HA3 3.842
384 G HN 8.477
384 G N 112.584

Table S2 | Cryo-EM structure determination statistics.

**Data collection**				
Microscope	Titan Krios G2			
Voltage [keV]	300			
Detector	K3			
Magnification	81,000			
Pixel size [Å]	1.05			
Defocus range [µm]	-0.7 to -2.0			
Exposure time [s/frame]	2.557			
Number of frames	40			
Total dose [e^-/Å^2]	40.94 (1.02 e^-/Å^2/frame)			
**Reconstruction**				
Picked segments	356,482			
Box width [pixels]	250			
Inter-box distance [pixels]	13			
Polymorph type	pm1	pm2	pm3	pm4
PDB-ID	7Q64	7Q65	7Q66	7Q67
EMDB-ID	13851	13852	13853	13854
Final segments [no.]	71,697	11,085	28,360	12,674
Final resolution [Å][a]	2.76	3.32	2.79	3.37
Sharpening B-factor [Å^2]	-92.90	-90.48	-80.93	-111.67
Symmetry imposed	C1	C1	C1	C1
Helical rise [Å]	4.65	4.68	4.67	2.37
Helical twist [°]	-3.09	-2.33	-3.29	178.49

[a] The resolution was estimated from the value of the FSC curve for two independently refined half-maps at 0.143.

Table S3 | Model building statistics.

	pm1	pm2	pm3	pm4
**PDB-ID**	7Q64	7Q65	7Q66	7Q67
**Model composition**				
Chains	30	22	22	11
Non-hydrogen atoms	7,809	6,204	4,807	2,618
Protein residues	1,100	880	682	363
**RMS deviations**				
Bond lengths [Å]	0.01	0.02	0.02	0.02
Bond angles [°]	1.34	1.67	1.55	2.21
**Validation**				
Model resolution (Å)	3.02	3.27	2.88	3.71
MolProbity score	2.34	2.40	2.26	2.96
Clashscore	7.37	3.45	13.59	12.16
**Ramachandran plot**				
Outliers [%]	0.00	0.00	0.00	0.00
Allowed [%]	8.29	10.53	0.00	16.13
Favored [%]	91.71	89.47	100.00	83.87
