# Supplementary material for: Molecular interactions of FG nucleoporin repeats at high resolution
Source: Nat Chem. 2022 Sep 22;14(11):1278–85. doi: 10.1038/s41557-022-01035-7 (PMC9630130; doi:10.1038/s41557-022-01035-7)
Supplement: Supplementary file 2 — Reporting Summary [file 41557_2022_1035_MOESM2_ESM.pdf]

nature research

Corresponding author(s): Markus Zweckstetter

Last updated by author(s): Jul 26, 2022

Reporting Summary

Nature Research wishes to improve the reproducibility of the work that we publish. This form provides structure for consistency and transparency in reporting. For further information on Nature Research policies, see our Editorial Policies and the Editorial Policy Checklist.

Statistics

For all statistical analyses, confirm that the following items are present in the figure legend, table legend, main text, or Methods section.

n/a	Confirmed	
☒	☐	The exact sample size (*n*) for each experimental group/condition, given as a discrete number and unit of measurement
☐	☒	A statement on whether measurements were taken from distinct samples or whether the same sample was measured repeatedly
☒	☐	The statistical test(s) used AND whether they are one- or two-sided<br>*Only common tests should be described solely by name; describe more complex techniques in the Methods section.*
☒	☐	A description of all covariates tested
☒	☐	A description of any assumptions or corrections, such as tests of normality and adjustment for multiple comparisons
☐	☒	A full description of the statistical parameters including central tendency (e.g. means) or other basic estimates (e.g. regression coefficient) AND variation (e.g. standard deviation) or associated estimates of uncertainty (e.g. confidence intervals)
☒	☐	For null hypothesis testing, the test statistic (e.g. *F*, *t*, *r*) with confidence intervals, effect sizes, degrees of freedom and *P* value noted<br>*Give P values as exact values whenever suitable.*
☒	☐	For Bayesian analysis, information on the choice of priors and Markov chain Monte Carlo settings
☒	☐	For hierarchical and complex designs, identification of the appropriate level for tests and full reporting of outcomes
☒	☐	Estimates of effect sizes (e.g. Cohen's *d*, Pearson's *r*), indicating how they were calculated

Our web collection on statistics for biologists contains articles on many of the points above.

Software and code

Policy information about availability of computer code

Data collection	NMR data were acquired using Topspin versions 3.5pl7 or 4.0.8. DLS data were acquired with DYNAMICS v7.10.0.23. CD data were acquired with Pro-Data Chirascan Spectrometer Control Panel Application v4.7.0.194. Optical microscope pictures are acquired with Leica Application Suite X. cryo-EM data were acquired using SerialEM Version 3.9.
Data analysis	NMR data were processed with Topspin 3.6.1 and analyzed using Sparky 3.115. Secondary structure and phi/psi angles were calculated with TALOS-N. PyMOL v1.8.4.0 was used to build the TALOS-N models and represent macromolecular structures. Optical microscope micrographs and FRAP experiments were analyzed with ImageJ v1.52a. DLS data were analyzed with DYNAMICS v7.10.0.23. Nup98FG85 fibrils were reconstructed using RELION-3.1 and the micrographs CTF corrected with CTFFIND4. The atomic models of Nup98FG85 fibrils were built in Coot 0.8.9.1. Refinement in real space was conducted using PHENIX 1.19.2 and Coot 0.8.9.1. The resulting models were validated with MolProbity 4.5. Stability calculations based on solvation energy were performed with the software accessiblesurfacearea_v07.2d. Graphpad Prism 7 was used for plotting data.

For manuscripts utilizing custom algorithms or software that are central to the research but not yet described in published literature, software must be made available to editors and reviewers. We strongly encourage code deposition in a community repository (e.g. GitHub). See the Nature Research guidelines for submitting code & software for further information.

Data

Policy information about availability of data

All manuscripts must include a data availability statement. This statement should provide the following information, where applicable:

- Accession codes, unique identifiers, or web links for publicly available datasets
- A list of figures that have associated raw data
- A description of any restrictions on data availability

Nuclear magnetic resonance chemical shifts are included in the extended data. Cryo-EM maps have been deposited in the Electron Microscopy Data bank (EMDB) under the accession numbers EMD-13851 (pm1), EMD-13852 (pm2), EMD-13853 (pm3), and EMD-13854 (pm4). The corresponding atomic models have been deposited in the Protein Data Bank (PDB) under the accession numbers: 7Q64 (pm1), 7Q65 (pm2), 7Q66 (pm3), and 7Q67 (pm4). All PDB codes cited (6XYO, 6IC3, 6SHS, 6NZN, 6VPS, 6SDZ, 6ZRF, 6XFM, 6MST, 6GK3, 7KWZ, 5O3O, 6TUB, 2RNM, 7BX7, 6WQK, 6UUR and 5V7Z) are publicly available in the PDB.

Field-specific reporting

Please select the one below that is the best fit for your research. If you are not sure, read the appropriate sections before making your selection.

☒ Life sciences ☐ Behavioural & social sciences ☐ Ecological, evolutionary & environmental sciences

For a reference copy of the document with all sections, see nature.com/documents/nr-reporting-summary-flat.pdf

Life sciences study design

All studies must disclose on these points even when the disclosure is negative.

Sample size	Sample size does not apply for macromolecular structural data.
Data exclusions	No data have been excluded while reporting this study.
Replication	NMR data was acquired with two batches of the Nup98FG protein (natural abundance and 15N, 13C labeled) getting same results. CD and DLS data were acquired twice with same results. EM and cryoEM pictures of the Nup98FG85 fibrils were acquired with 5 samples getting always similar fibrils; the best one was used for the acquisition. Light microscopy images were acquired with two samples getting similar pictures.
Randomization	Randomization was not relevant to this study, because we are not working with any particular population (there is no population to randomly select from) so our experiments can be repeated.
Blinding	Blinding was not relevant to this study, because decision-making has no impact on the experiment and there is no risk of bias.

Reporting for specific materials, systems and methods

We require information from authors about some types of materials, experimental systems and methods used in many studies. Here, indicate whether each material, system or method listed is relevant to your study. If you are not sure if a list item applies to your research, read the appropriate section before selecting a response.

Materials & experimental systems

n/a	Involved in the study	
☒	☐	Antibodies
☒	☐	Eukaryotic cell lines
☒	☐	Palaeontology and archaeology
☒	☐	Animals and other organisms
☒	☐	Human research participants
☒	☐	Clinical data
☒	☐	Dual use research of concern

Methods

n/a	Involved in the study	
☒	☐	ChIP-seq
☒	☐	Flow cytometry
☒	☐	MRI-based neuroimaging
